# High quality implementation of 4Rs + MTP increases classroom emotional support and reduces absenteeism

**DOI:** 10.3389/fpsyg.2023.1065749

**Published:** 2023-04-27

**Authors:** John A. Gómez, Joshua L. Brown, Jason T. Downer

**Affiliations:** ^1^Applied Developmental Psychology, Department of Psychology, Fordham University, Bronx, NY, United States; ^2^Center for Advanced Study of Teaching and Learning, School of Education and Human Development, University of Virginia, Charlottesville, VA, United States

**Keywords:** quality of implementation, school program, social and emotional learning, classroom interactions, compliance propensity

## Abstract

School-based social and emotional learning (SEL) programs are associated with improvements in children’s SEL and academic outcomes, and the quality of classroom interactions. The magnitude of these effects increases at high levels of program implementation quality. This study aimed to (1) identify teachers’ profiles of quality of implementation, (2) explore teachers and classroom characteristics contributing to their propensity to comply with high quality of implementation, and (3) examine the relations between school assignment to an SEL program, quality of classroom interactions, and child SEL and academic outcomes at different levels of teachers’ compliance propensity. This study drew upon data from a cluster-randomized controlled trial evaluating the efficacy of 4Rs + MTP, a literacy-based SEL program, on third and fourth grade teachers (*n* = 330) and their students (*n* = 5,081) across 60 New York City public elementary schools. Latent profile analysis indicated that measures of teacher responsiveness and amount of exposure to implementation supports contributed to the differentiation of profiles of high and low quality of implementation. Random forest analysis showed that more experienced teachers with low levels of professional burnout had high propensity to comply with high quality of implementation. Multilevel moderated mediation analysis indicated that 4Rs + MTP teachers with high compliance propensity were associated with higher classroom emotional support and lower children’s school absences than their counterparts in the control group. These findings may inform debates in policy research about the importance of providing the supports teachers need to implement SEL school programs with high quality.

## 1. Introduction

### 1.1. School-based social and emotional learning interventions and child development

School-based social and emotional learning (SEL) interventions encompass a series of intentional program efforts designed to promote children’s learning and application of social, emotional and character skills required to succeed in school, workplace settings, relationships, and citizenship (Jones et al., 2019; Weissberg, 2019). Efficacy studies of such programs show that, relative to children in control conditions, children participating in these programs show improvements in their social and emotional skills, positive attitudes toward self and others, positive social behavior, fewer conduct problems, lower emotional distress, and have higher academic achievement scores (Durlak et al., 2011; Grant et al., 2017; Taylor et al., 2017).

SEL interventions in schools typically have the objective of both teaching students specific social and emotional skills and creating caring and supportive classroom interactions where such skills flourish (Brown et al., 2010; Brackett et al., 2012). According to the Teaching through Interactions Framework (Hamre and Pianta, 2007; Hamre et al., 2013), quality of classroom interactions is organized into three domains: Emotional Support, Classroom Organization, and Instructional Support. High quality interactions within each of these domains are hypothesized to promote students’ learning and social development (Hamre and Pianta, 2007; Pianta and Hamre, 2009). A randomized controlled trial of one school-based SEL program, Reading, Writing, Respect and Resolution (4Rs), showed positive effects on quality of third-grade classroom interactions as measured by independent observers (Brown et al., 2010). Moreover, children in schools implementing 4Rs showed lower hostile attribution biases and fewer depressive symptoms at the end of the first year compared to children in control schools (Jones et al., 2010), benefits that persisted and expanded to other outcome domains including teacher-reported attention skills, and aggressive and socially competent behaviors, following a second year of program implementation (Jones et al., 2011). These findings suggest that SEL programs improve teachers’ support during classroom interactions, which may in turn lead to other benefits for children experiencing these higher quality classroom interactions. A developmental systems approach to the evaluation of school-based SEL programs affords comprehensive interpretations of changes in children’s social and emotional functioning within contexts that provide rich and nurturing interactions (Roeser et al., 2000; Hamre and Pianta, 2005). However, no research to date has tested the quality of classroom interactions as a mediator of the effect of school-based SEL programs on children’s developmental outcomes.

### 1.2. Quality of implementation

Research in program evaluation suggests that the positive effects of school-based interventions on children’s outcomes depends largely on the quality of program implementation (Dane and Schneider, 1998; Fixsen et al., 2009; Lendrum and Humphrey, 2012; Durlak, 2016). For instance, in a meta-analytic study, Durlak et al. (2011) found that children in better-implemented SEL programs compared to poorly implemented programs showed greater gains in academic achievement and larger reductions in conduct problems and emotional distress. Similarly, in an elementary school mental health program, Dix et al. (2012) found that the difference between children in high- and low-implementation schools represented a difference in academic performance favoring children in high implementation schools equivalent to 6 months of schooling.

**Figure 1:**
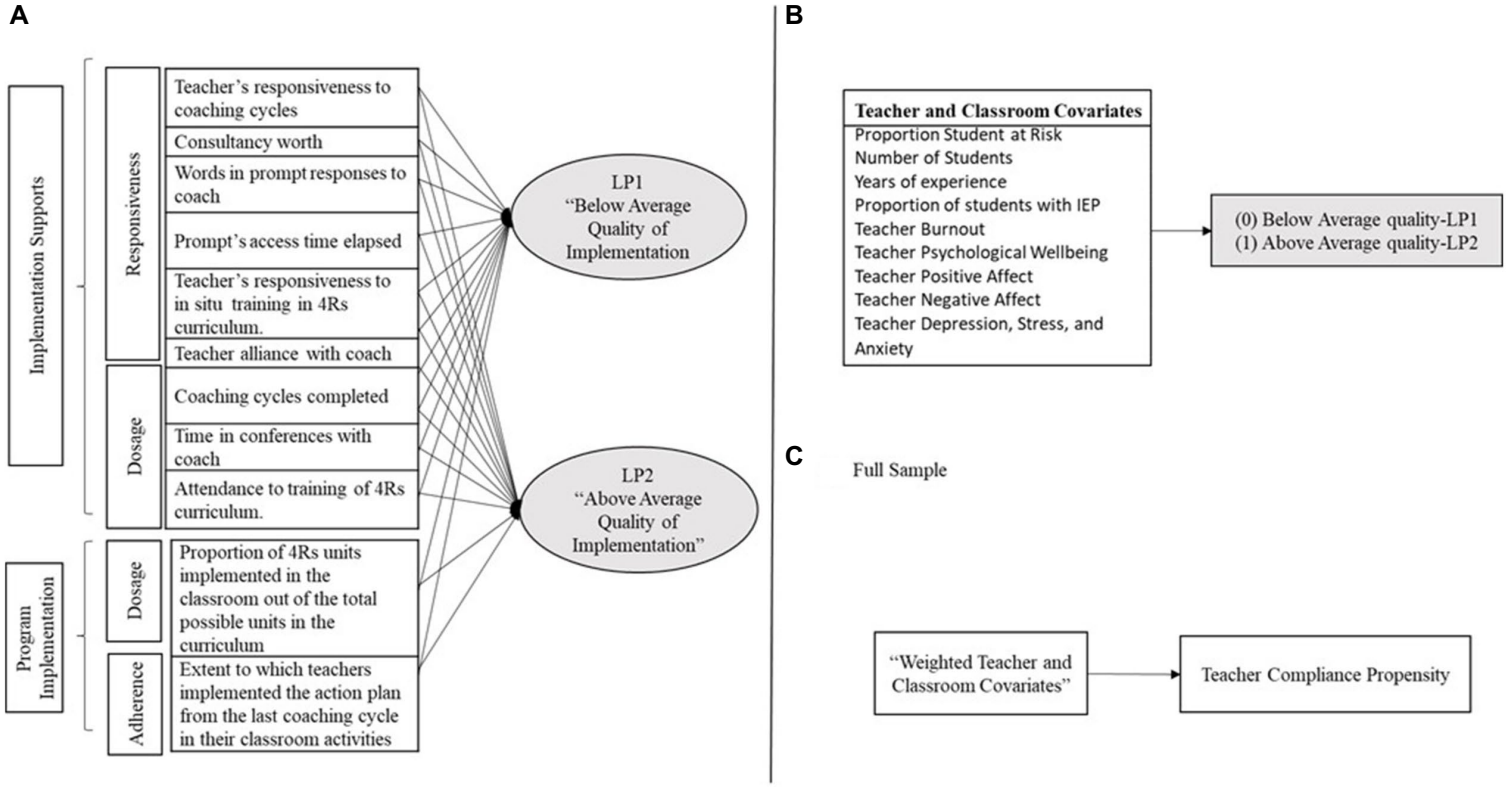
**(A–C)** Models guiding the estimation of teacher compliance propensity.

One of the limitations of most implementation studies is an over-emphasis on fidelity of implementation of program activities, that is, the extent to which the program was implemented as planned (Dane and Schneider, 1998). In this regard, measures such as the amount of program activities participants implemented (dosage) and participant enactment of program protocols (adherence) are considered to determine how well the program was implemented (Fixsen et al., 2009). Less attention has been paid to other factors associated with participants’ responsiveness to program implementation and quality of delivery of program activities (Durlak and DuPre, 2008; Lendrum et al., 2016). In addition, implementation studies have overlook the importance of factors associated with the supports teachers need for effective program delivery (Domitrovich et al., 2010), such as on-going coaching, training, and modeling from program experts (Hadden and Pianta, 2006; Kraft et al., 2018). Over the past two decades, the operationalization of fidelity of implementation has been extended to incorporate the construct of quality of implementation, including various measures of the amount and quality of both program implementation and implementation supports (Domitrovich et al., 2010; Dix et al., 2012). Figure 1A illustrates the model we use to distinguish program implementation from implementation supports in this study.

Previous research on school-based SEL interventions has found associations between several measures of program implementation and implementation supports and child and teacher outcomes. For instance, high quality of program delivery by teachers and children’s responsiveness to the intervention has been associated with reductions in children’s aggressive behavior and improvements in academic outcomes (Domitrovich et al., 2010; Humphrey et al., 2016). High program dosage and adherence have also been associated with improvements in children’s social competence (Rosenblatt and Elias, 2008). Similarly, teachers’ adherence to SEL program practices has been linked to gains in teachers’ quality of interactions with children in their classrooms (Abry et al., 2013). Teachers’ access to on-going coaching has also been linked to improvements in teacher emotional and instructional support in their classroom interactions with children (Pianta et al., 2008).

Program implementation and implementation supports are also associated. For instance, teachers attending high numbers of coaching sessions are also more likely to implement a higher number of program activities than teachers with few coaching sessions (Pas et al., 2015), and the strength of the teacher-coach working alliance has been associated with higher teacher adherence to program protocols (Wehby et al., 2012). Given this dynamic interaction between program implementation and implementation supports, it is pertinent to examine these two factors simultaneously in models evaluating the effect of quality of implementation as a whole on targeted program outcomes (Durlak and DuPre, 2008; Domitrovich et al., 2010). However, including several interrelated measures of implementation and implementation supports in a single model increases the risk of multicollinearity and the probability of Type I error. In this scenario, a comprehensive methodological approach that integrates measures of program implementation and implementation supports into a global index of quality of implementation may be a reasonable alternative to investigate the effects of implementation quality on program outcomes (Dix et al., 2010).

One example of a comprehensive measure of implementation is provided by Dix et al. (2012) in the study of the effects of a two-year SEL program (KidsMatter) on the academic outcomes of children from 100 elementary schools. Guided by the framework proposed by Domitrovich et al. (2008), an overall index of school implementation was created to sort schools into high and low implementation groups. The index was created using Latent Class Analysis (LCA) with a total of 37 items measuring adherence, dosage, and quality of delivery of both program implementation and implementation supports. However, only 13 items were found to differentiate adequately between high and low implementing schools; these particular items corresponded to adherence and quality of delivery of implementation support, as well as dosage of program implementation (Slee et al., 2009). This index successfully predicted better academic outcomes (in Literature and Mathematics) for children in high, compared to low, implementing schools. The difference between children from low and high implementing schools was robust to school level SES and was equivalent to a difference in academic performance of up to 6 months of schooling (Dix et al., 2012).

Research testing the effects of quality of implementation for core program components on program outcomes, or using multidimensional measures of implementation (e.g., dosage, adherence, responsiveness, quality of delivery), often restrict their study to the treated sample. Thus, the generalization of results is restricted to individuals sharing characteristics with one subsample of the study. Information about the quality of implementation is supposed to yield better insights into program related causes of the treatment effects; however, access to limited information about implementation and only in the treated sample is not sufficient for making better causal inferences about the treatment effects in the population.

### 1.3. Implementation and program effects

Recently, some studies are using a method to estimate the causal effect of complying with implementation of SEL interventions on child and teacher outcomes (Berg et al., 2017; Panayiotou et al., 2019), called Compliers Average Causal Effect (CACE). CACE is a method developed originally by Imbens and Rubin (1997) to identify differences in medical program outcomes between those who received the treatment and those who did not in both treatment and control groups. This approach requires clear cut-offs regarding participants who will be considered compliers, in order to make effective comparisons between participants in the treatment condition who did not comply with treatment and participants in the control condition who did not receive treatment (Follmann, 2000). In studies of SEL programs using CACE, compliers are often defined with a single measure of dosage (e.g., those who implemented one standard deviation above the median number of program activities; Panayiotou et al., 2019). In education research, this approach is limited. In addition to the term “complier” applied to an active and autonomous agent such as teachers implementing a program in their own field, it assumes those teachers who implemented less than the cut-off score (e.g., 1/3 of the curricular activities) and their children who received less program dosage are comparable with those in the control group who never implemented/received the intervention (Sagarin et al., 2014). However, research in SEL implementation has found that even when program dosage is low, the quality of delivery may make a difference in terms of program outcomes (Durlak and DuPre, 2008; Humphrey et al., 2018). Therefore, using a single measure of dosage to estimate CACE is similar to an arbitrary decision.

Other methods have been proposed that do not require equating noncompliers in a treatment group with participants in a control group (Sagarin et al., 2014). A propensity score approach has been proposed by Follmann (2000) to estimate the probability of compliance using covariates. This method allows for the estimation of, for example, teacher compliance propensity using teacher and classroom characteristics (covariates) known to predict high quality implementation among teachers (Downer et al., 2009b; Domitrovich et al., 2019). For instance, teachers in classrooms with high proportions of at-risk students and high emotional exhaustion (burnout) have shown lower implementation of SEL activities in the classroom (Musci et al., 2019). Therefore, using baseline covariates as predictors of compliance in the treatment group, it is possible to predict compliance propensity in the control group with similar baseline covariates. Translating this approach to a school-based SEL intervention context, compliance propensity could be estimated using baseline covariates as predictors of high and low quality of implementation in the treatment group and, using similar covariates, also predict compliance propensity in the control group. Since the propensity approach does not require equating noncompliers in the treatment group with select participants in the control group, teacher compliance propensity can be estimated using several indicators of high quality of implementation, in addition to dosage.

The magnitude of treatment effects at different levels of teacher compliance propensity may provide valuable information about how the high quality of implementation in SEL program may influence program effects on teachers and children. Particularly, this high quality of implementation may increase the effects of SEL programs in improving classroom interactions where children thrive with opportunities to learn and develop social and emotional skills needed to succeed in life. However, there is no research to date using compliance propensity in evaluations of SEL program implementation.

### 1.4. The current study

**Figure 2:**
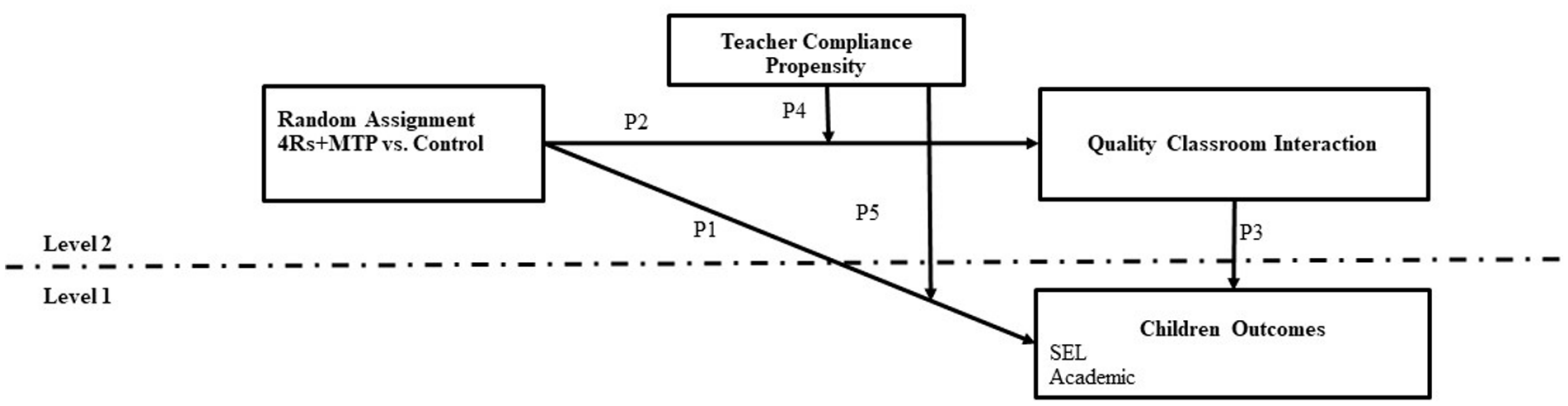
Multilevel moderated mediation model. Level 1 refers to variables at the child level, and level 2 refers to variables at the teacher and classroom level. P1–P5 refer to paths representing the relationships between variables.

This study aims to understand the effects of an SEL program on classroom interactions and child SEL and academic outcomes as moderated by teachers’ compliance propensity. Furthermore, this study examines the role of classroom quality of interactions as a mediator of the relationship between an SEL program and child outcomes at different levels of teachers’ compliance propensity (see Figure 2). This study drew upon data collected as part of a cluster-randomized controlled trial evaluating the efficacy of a literacy-based SEL program (4Rs + MTP) implemented and tested in two consecutive cohorts of students within 60 New York City (NYC) public elementary schools during the 2015–2016 and 2016–2017 school years. The 4Rs + MTP program integrates two distinct and complementary evidence-based interventions: Reading Writing, Respect and Resolution (4Rs), a universal, school-based program integrating social and emotional competencies into the language arts curriculum for grades K-5 (Jones et al., 2011), and MyTeachingPartner (MTP), a coaching model that is based on providing teachers with personalized feedback and on-demand support of curriculum implementation, through web-based and *in situ* teacher-coach interaction (Pianta et al., 2008). The 4Rs program uses an ecological developmental approach (Bronfenbrenner and Morris, 1998) which posits that children develop negotiation strategies in interpersonal interactions within specific contexts. Accordingly, the 4Rs program includes social-cognitive processes associated with aggressive behaviors (e.g., hostile attribution bias), and classroom quality of interactions as proximal outcomes in their theory of change (Aber et al., 1998, 2011). Activities in the 4Rs program involve the selection of high-quality children’s literature that invites children, with the guidance of their teachers, to learn how to handle anger and use skills like listening, cooperation, assertiveness, and negotiation during interpersonal conflicts in classroom (Aber et al., 2011). The MyTeachingPartner (MTP) coaching approach draws on attachment theory as instantiated within classroom interactions (Hamre et al., 2013), positing that the quality of teacher-student interactions is pivotal for student learning, with a particular focus on the support teachers provide to create caring and trusting relationships with their students. The integration of the 4Rs program and the MTP coaching approach extends the focus of 4Rs in promoting positive interpersonal relationships in classrooms, including activities to promote high quality child-teacher relationships along with the activities to promote positive interpersonal negotiation strategies with peers. The MTP coaching approach also includes ongoing coaching to support teachers’ implementation of the 4Rs program curriculum. In sum, the 4Rs + MTP program aims to provide a systematic and comprehensive approach to in-person training on quality of classroom interactions and 8 cycles of video-based and web-mediated coaching focused on teachers’ implementation of the 4Rs curricular units in the classroom (e.g., lessons, book talk) that target the development of social and emotional competencies within the language arts. The program is designed to promote teachers’ psychological well-being, and high-quality classroom interactions that foster student learning, and the development of students’ social and emotional competencies and academic functioning. Preliminary findings based on the intent-to-treat analyses from the efficacy trial of the 4Rs + MTP program demonstrate positive intervention effects after 1 year of 4Rs + MTP implementation on teacher anxiety and stress, observation-based ratings of emotionally supportive interactions in classrooms, and children’s social competence, aggressive behavior and conduct problems as reported by teachers controlling for baseline scores on each outcome (Brown et al., 2019).

The specific aims of this study were twofold. The first aim was to estimate teacher compliance propensity. Since there is no research to date estimating propensities of quality of implementation, the research questions for this objective are exploratory in nature, although partially informed by previous research on the quality of program implementation. Here we explore which components of program implementation and implementation supports would discriminate between profiles of quality of implementation for teachers in the treatment group (see Figure 1A). Previous work has found that both measures of implementation and implementation support successfully allow the classification of schools into high and low quality of implementation classes (Dix et al., 2010). We then explore teacher and classroom covariates that predict teacher compliance propensity for teachers in the treatment group (see Figure 1B) and the full sample (treatment and control groups; see Figure 1C). Previous research has found that teacher age, job burnout, and baseline quality of classroom interactions, and classroom characteristics such as percentage of behaviorally at risk children, are associated with quality of implementation (Downer et al., 2009b; Berg et al., 2017; Domitrovich et al., 2019). The second aim of this study was to examine teacher compliance propensity as a moderator of the relationship between school random assignment to 4Rs + MTP and (a) quality of classroom interaction (see Figure 2, paths P2 and P4), and (b) children’s SEL and academic outcomes (see Figure 2, paths P1 and P5). And (c) children’s SEL and academic outcomes as mediated by the quality of classroom interaction (see Figure 2, paths P2, P4, and P3)?

It was expected that teachers randomly assigned to 4Rs + MTP would have higher quality classroom interactions than teachers in the control group, when examined at an above average level of compliance propensity. Previous findings show that teachers who receive consultation and on-going support from coaches, compared with no personalized support, were able to provide better support to their students in the classroom (Pianta et al., 2008; Early et al., 2017). It was also expected that teachers with above average compliance propensity in schools randomly assigned to 4Rs + MTP would have children with higher social–emotional and academic outcomes than children of teachers in the control group. The literature on quality of implementation indicates that, in contrast to poorly implemented programs, well-implemented programs are associated with increases in children’s prosocial behavior (Rimm-Kaufman et al., 2007) and academic achievement (Dix et al., 2012), and reductions in conduct problems and emotional distress (Durlak and DuPre, 2008; Durlak et al., 2011) as well as unexcused absences from school (Neace and Munoz, 2012). Lastly, it was expected that teachers with above average compliance propensity in schools randomly assigned to 4Rs + MTP would have children with higher social–emotional and academic outcomes as mediated by higher quality of classroom interactions than children of teachers in schools randomly assigned to the control group. Previous research has found that supportive and nurturing interactions in the classroom are associated with better child behavioral (Portnow et al., 2018; Rucinski et al., 2018) and cognitive outcomes (Curby et al., 2009; Reyes et al., 2012). These findings along with evidence regarding positive effects of SEL interventions and quality of implementation on classroom quality and child outcomes suggest that children may benefit from SEL interventions through the effects on the quality of classroom interactions when interventions are well implemented.

## 2. Materials and methods

### 2.1. Participants

Data for this study were collected across two cohorts (2015–2016; 2016–2017) as part of the randomized controlled trial (RCT) of the 4Rs + MTP program. Across both cohorts, the study sample is comprised of 5,081 third- and fourth-grade children (treatment *n* = 2,326; control *n* = 2,755) taught by 334 teachers (treatment *n* = 151; control *n* = 183) from 60 urban, high needs elementary schools (treatment *n* = 31, control *n* = 29). Teachers in the treatment group were each assigned one of seven dedicated 4Rs + MTP coaches. There was a similar proportion of teachers in third (45.5%) and fourth (44%) grades, and most were female (90.9%). The ethnic/racial composition of teachers were White (38.9%), Hispanic/Latina (27.8%) and Black or African American (21.9%). On average, each classroom had approximately 22 children, and on average, 15 children per classroom (*SD* = 5.12) participated in the study. Table 1 shows teacher and classroom demographic characteristics.

**Table 1:** Teacher and classroom demographic characteristics (*n* = 334).

			Treatment		Control		Total	
			*n*	*%*	*n*	*%*	*n*	*%*
Gender	Female		136	90.1	164	90	300	90.9
	Male		14	9.2	16	9	30	9.1
	Missing		1	0.7	3	2	4	1.2
Race	White		46	30.5	73	40	119	38.9
	Hispanic or Latina		40	26.5	45	25	85	27.8
	Black-African American		41	27.1	26	14	67	21.9
	Multi racial		7	4.6	15	8	22	7.2
	Asian		4	2.6	6	3	10	3.3
	Other		2	1.3	0	0	2	0.6
	Hispanic and black		1	0.6	0	0	1	0.3
	Missing		10	6.6	18	10	28	8.38
Teaching certificate	Regular or standard state		123	81	133	73	256	78.8
	Provisional or other type		3	1.9	11	6	14	4.3
	Probationary certificate		19	12.6	24	13	43	13.2
	Temporary certificate		3	1.9	2	1	5	1.5
	Other certificate		1	0.6	6	3	7	2.1
	Missing		2	1.3	7	4	9	2.69
Highest degree	Bachelor’s degree		8	5.0	10	5	18	5.5
	Master’s degree		138	91	164	90	302	92.3
	Specialists degree		2	1.0	2	1	4	1.2
	Doctorate degree		2	1.0	0	0	2	0.6
	Other degree		0	0.0	1	1	1	0.3
	Missing		1	1.0	6	3	7	2.1
Random assignment			151	100.0	183	100	334	100.0
Grade	3rd grade		67	44.0	80	44	147	45.5
	4th grade		64	42.0	78	43	142	44.0
	Mixed		17	11.0	17	9	34	10.5
	Missing		3	2.0	8	4	11	3.3
Classroom type	General education		90	60	102	56	192	59.8
	ICT/CTT/inclusion		37	25	46	25	83	25.9
	Self-contained special Ed		21	14	25	14	46	14.3
	Missing		3	2	10	5	13	3.9
Language program	None/English		132	87	147	80	279	86.4
	Dual language		5	3	10	5	15	4.6
	Bilingual		9	6	4	2	13	4.0
	ENL/ESL		2	1	14	8	16	5.0
	Missing		3	2	8	4	11	3.3
	**Tx**		**Ct**		**Total**			
	***M***	***SD***	***M***	***SD***	***M***	***SD***	**Min**	**Max**
Years of teaching experience	11.99	8.60	9.54	6.40	10.70	7.60	1	40
Years at current school	8.03	7.23	6.39	5.61	7.10	6.40	1	31
Class size	21.55	5.77	23.11	5.97	22.40	5.90	6	33
Proportion of girls	0.47	0.14	0.47	0.12	0.48	0.13	0	1
Proportion of IEP	0.13	0.23	0.18	0.25	0.16	0.23	0	1
Proportion of LEP	0.29	0.33	0.29	0.33	0.29	0.33	0	29

ICT, Integrated Co-Teaching; CTT, Collaborative Team Teaching; IEP, Individualized Education Plan; LEP, Limited English proficiency.

Children were 51.6% female with an average age of 8.8 years old (range: 5–12 years old), 65% Latine, 22% Black, 6% White, 5% Asian, and 2% other. Table 2 presents demographic characteristics of children in the sample.

**Table 2:** Child demographic characteristics.

		Treatment		Control		Total	
		*n*	*%*	*n*	*%*	*n*	*%*
School random assignment		2,326	46	2,755	54	5,081	100.0
**Missing**							
Gender	Female	1,216	52.27	1,404	50.96	2,620	51.56
	Male	1,089	46.83	1,336	48.50	2,425	47.73
Race	Latine	1,501	64.53	1,816	65.92	3,317	65.28
	Black	613	26.35	514	18.66	1,127	22.18
	White	113	4.86	197	7.15	310	6.10
	Asian	50	2.15	182	6.61	232	4.57
	Multiracial	18	0.77	19	0.69	37	0.73
	Native American	10	0.43	12	0.44	22	0.43
Missing		21	0.90	15	0.54	36	0.71
		*M*	*SD*	*M*	*SD*	*M*	*SD*
Age		8.78	0.79	8.81	0.80	8.80	0.8
		Min	Max	Min	Max	Min	Max
		6	12	5	12	5	12

### 2.2. Procedures

This randomized controlled study of the 4Rs + MTP program was carried out in two phases for each of two consecutive cohorts of participating schools. The first phase included school recruitment, school random assignment to treatment and control conditions, and consenting of teachers and children. The second phase included data collection and program implementation which took place during one school year. A timeline of activities during these two phases of the study is provided in Table 3.

**Table 3:** Study timeline.

School year	2014–2015		2015–2016				2016–2017			
	SP	SU	F	W	SP	SU	F	W	SP	SU
**Cohort 1**										
School recruitment	×									
Teacher consent		×								
Random assignment		×								
Student consent			×							
4Rs + MTP implementation and professional development			×	×	×					
Wave 1 data collection (G3&4)				×						
Wave 2 data collection (G3&4)					×					
**Cohort 2**										
School recruitment					×					
Teacher consent					×	×				
Random assignment					×					
Student consent							×			
4Rs + MTP implementation and professional development							×	×	×	
Wave 1 data collection (G3&4)							×	×		
Wave 2 data collection (G3&4)									×	

G3, Grade 3 teachers, students, and classrooms; G4, grade 4 teachers, students, and classrooms. SP, Spring; SU, Summer; F, Fall; W, Winter.

Before the school random assignment process, third and fourth-grade teachers from each participating school were sent *via* email consent forms explaining the purpose of 4Rs + MTP and training and implementation procedures for the treatment group, and emphasizing that participation in the study was voluntary. Across cohorts there were a total of 444 eligible 3rd and 4th grade teachers out of which 336 consented to participate (76% overall, 76% in treatment group and 75% in control group). Trained research team members visited classrooms of all participating teachers and provided students with a brief, age-appropriate explanation of the study and the procedures for data collection. Students received consent forms in English and Spanish to take home to their caregivers/guardians. Students who returned a consent form signed by their parent/guardian indicating either consent or denial, were given a new grade-appropriate children’s book. From the 7,708 eligible students across cohorts, 5,081 (66%) were consented (treatment *N* = 2,326; control *N* = 2,755). All study procedures were approved by the local Department of Education.

Participating teachers and children completed baseline measures during Fall/Winter (wave 1) and end of year measures during Spring (wave 2). At each wave, teachers completed a battery of assessment for each of the consented children in their classrooms, as well as their own self-assessments, including demographic information about themselves and their classrooms. Children completed self-assessments *via* classroom administration of paper and pencil surveys, and trained observers visited each classroom to observe and rate the quality of classroom interactions and teaching practices.

#### 2.2.1. Implementation of the 4Rs + MTP program

The 4Rs + MTP program implementation includes professional development for teachers in support of their effective delivery of the 4Rs curriculum in their classrooms. Seven experienced former educators served as 4Rs + MTP coaches and provided six in-person training sessions followed by eight cycles of one-on-one coaching to teachers in 4Rs + MTP schools, emphasizing content and strategies aimed to improve the quality of interactions in the classroom *via* the Teaching through Interactions framework (Hamre et al., 2013). Below, procedures for each component of 4Rs + MTP program are described.

##### 2.2.1.1. The 4Rs program

The 4Rs component of the program consists of a curriculum divided into seven units, each focused on promoting skills to understand and handle feelings, listening to others, establish nurturing relationships through cooperation, negotiation, and building community. In total, 4Rs offers 66 in-class activities for third grade and 70 for fourth-grade classrooms. 4Rs includes a professional development component for teachers, consisting of six in person training sessions, each 6 h long, aimed to equip teachers with the knowledge and techniques needed to implement the 4Rs curriculum effectively in classrooms. Teachers also received *in situ* support from coaches to model, co-teach, and provide feedback on teacher implementation of 4Rs curricular activities.

##### 2.2.1.2. MyTeachingPartner coaching

The MyTeachingPartner (MTP) component of the program provided teachers with support and professional development focused on one-to-one video-based coaching and access to video exemplars through a web-based interactive platform. 4Rs + MTP included eight video-based teacher-coach cycles, each focused on coaches (a) observing video recordings from each of their teachers’ classrooms to identify effective teacher-child interactions during 4Rs + MTP implementation; (b) providing each of their teachers with written prompts to focus teachers’ attention and generate teacher reflection on their interactions with students during the program implementation; and (c) establishing a supportive and reliable coach-teacher alliance where teachers felt comfortable asking questions and reflecting on their challenges without feeling judged or evaluated (Hadden and Pianta, 2006). Each cycle lasted approximately 4 to 5 weeks and was repeated eight times during the year of 4Rs + MTP implementation.

Data were collected on teachers’ implementation of program activities in the classroom and the implementation supports they received through training and coaching. Teachers in the treatment group used the online MTP web platform to keep weekly logs of their implementation of 4Rs + MTP lessons and units. Additionally, the MTP web platform registered information about teachers’ logins and visits to the platform’s webpages and the duration of these visits.

### 2.3. Measures

#### 2.3.1. Classroom interactions and children’s SEL and academic outcomes

##### 2.3.1.1. Student academic competence and school attendance

Student academic competence was measured using the New York State standardized English Language Arts (ELA) and Math achievement tests. The ELA population-scale score mean was 599.79 (*SD* = 20.22), and 599.77 (*SD* = 20.17) for third and fourth grades, respectively. Math population-scale score means for third and fourth grade in 2018 was 599.48 (*SD* = 20.19), and 599.38 (*SD* = 20.23).

Children’s school attendance was measured using direct reports of class attendance from the local Department of Education (DOE) records at the end of the year prior to the start of the study and again at the end of the main year of the study in each cohort. The DOE attendance data provides information on children’s number of days absent and number of days present during the school year. Baseline measures of attendance correspond to the total days present in the year prior to intervention delivery which was 2014–2015 for cohort 1 and 2015–2016 for cohort 2, whereas the end of the year measures of attendance corresponds to data from 2015 to 2016 for cohort 1 and 2016–2017 for cohort 2, the end of the main year of the study in each cohort.

##### 2.3.1.2. Anxious and depressive symptoms (child report)

Children’s self-reported depressive and anxiety symptoms were measured through the depression subscale and anxiety subscale of the Behavioral Assessment System for Children (BASC; Kamphaus and Reynolds, 1998). The depression subscale is comprised of 13 true/false statements, such as “Nothing ever goes right for me.” Children’s anxious symptoms were measured using the self-report 13-item anxiety subscale of BASC. An example item includes, “I get so nervous I cannot breathe.” Scale reliabilities ranged from α = 0.85 in fall/winter to α = 0.85 in spring.

##### 2.3.1.3. Aggressive behavior (child report)

Children reported on their own aggressive behaviors using the Aggression Scale (Orpinas and Frankowski, 2001). This scale is comprised of six items that ask children to report how many times they have engaged in specific aggressive behaviors over the past couple of weeks (0 = Never; 1 = Once or twice; 2 = A few times; and 3 = Many times). Examples of items are “I teased a kid at school” and “I pushed, shoved, or hit a kid at school.” Scale reliabilities ranged from α = 0.82 in fall/winter to α = 0.79 in spring.

##### 2.3.1.4. Aggressive behavior (teacher report)

Children’s aggressive behaviors were assessed using teachers’ reports on the aggression subscale of the Behavioral Assessment System for Children (BASC-AGG; Kamphaus and Reynolds, 1998). Teachers responded 14 questions regarding the frequency of a particular child behavior over the past 30 days on a 4-point scale (1 = Never; 2 = Sometimes; 3 = Often; 4 = Almost always). Example items included “Argues when denied own way” or “Is a sore loser.” Cronbach’s alphas for this study ranged from α = 0.92 in fall/winter to α = 0.91 in spring.

##### 2.3.1.5. Student social competence (teacher report)

To assess student social competence, an average of 19-items from the teacher-reported Social Competence Scale (Conduct Problems Prevention Research Group, 1990) was used. Teachers rated 19 items regarding child behavior over the past 30 days on a 4-point scale (1 = Never; 2 = Sometimes; 3 = Often; 4 = Almost always). Sample items included “Expresses needs and feelings appropriately” and “Cooperates with peers without prompting.” Internal consistency was high across the fall/winter and spring waves (α = 0.96 and α =0.98, respectively).

##### 2.3.1.6. Hostile attribution bias (child report)

Children’s self-reported hostile attribution bias (HAB) was measured using a 6-item adaptation of the Home Interview (Dahlberg et al., 1998) developed initially by Dodge et al. (1986). In this version, children are presented with six visual and verbal representations (vignettes) of ambiguous but provocative social scenarios. Following the presentation of each vignette, children were presented with four possible causal attributions regarding the intent of the provocateur and were asked to select one causal attribution. Two attributions refer to the provocateur’s intent as benign or accidental = 0 (e.g., The ball slipped and hit you), and two responses describe the provocateur’s intent as hostile or purposeful = 1 (e.g., the student was being mean). Responses were coded as either 1 (hostile) or 0 (benign), and then averaged across items, with higher scores indicating greater hostile attribution bias. This measure had adequate internal consistency across both assessment waves (α’s = 0.74 to 0.78).

##### 2.3.1.7. Aggressive interpersonal negotiation strategies (child report)

Following the assessment of their attributions of intent with scenarios from the Home Interview described above (Dahlberg et al., 1998), children were asked what they would do next in each of the six scenarios and then were asked to select one from among four possible response strategies. Responses were coded as either 1 (aggressive; e.g., Break something that belongs to that child) or 0 (non-aggressive; e.g., Not play with the child again) and then averaged across items. The *Aggressive Interpersonal Negotiation Strategies* score is created averaging children’s responses across items, with higher scores indicating greater tendencies to react aggressively. Internal consistencies ranged from α = 0.86–0.87.

##### 2.3.1.8. Quality of classroom interactions (observer rated)

The quality of classroom interactions was measured using the Classroom Assessment Scoring System-Upper Elementary Version (CLASS-UE; Pianta et al., 2012). CLASS-UE is an observational measure to evaluate three domains of teacher-student interactions: emotional support, classroom organization, and instructional support.

Emotional support refers to a teacher’s skills and strategies in providing safe and supportive environments, where students feel secure and become more self-reliant in their explorations during problem-solving situations, feel positively related to others, and autonomous (Roeser et al., 2000; Hamre and Pianta, 2005). The domain of emotional support consists of three dimensions: positive climate, teacher sensitivity, and regard for student perspectives. Classroom organization refers to a teacher’s competence in providing structured, organized, and sequenced practices that help develop children’s self-regulatory skills (Blair, 2002). The domain of classroom organization consists of three dimensions: behavior management, productivity, and negative climate (reverse-coded). Instructional support refers to the pedagogical strategies a teacher uses to help children develop a sense of curiosity for learning, think about their learning and thinking processes (Baird, 1986), and in general, promote cognitive and language development in the classroom (Hamre et al., 2013). The instructional support domain consists of five dimensions: instructional learning formats, content understanding, analysis and inquiry, quality of feedback, and instructional dialog. Each dimension of CLASS is rated on a scale of 1 (low) to 7 (high).

A live classroom observation was conducted in each participating teacher’s classroom. A team of 18 classroom observers who were trained to reliability and certified on the CLASS-UE conducted the observations, and a single observer rated each of the 11 dimensions for each classroom. Domain scores were then calculated by taking the average of the dimension scores within each domain.

As we have reported elsewhere (Doyle et al., 2022), interrater reliability (IRR) was calculated using the 50 observations (16%) that were double-coded at observation 1 and the 39 observations (12%) that were double-coded at observation 2. IRR was calculated using a one-way random intraclass correlation (ICC), which captures rater consistency across two measured constructs (Shrout and Fleiss, 1979). The ICC is a conservative measure of interrater reliability, as it includes both the variability within and across observers. ICCs can range from −1 to +1, with values less than 0.5 indicating poor reliability, values between 0.50 and 0.75 indicating moderate reliability, values between 0.75 and 0.90 indicating good reliability, and values greater than 0.90 indicating excellent reliability (Koo and Li, 2016). In the current study, ICCs were 0.62 and 0.74 for Emotional Support, 0.45 and 0.88 for Classroom Organization, and 0.59 and 0.72 for Instructional Support at observation 1 and observation 2, respectively.

#### 2.3.2. 4Rs + MTP program implementation and supports

All measures of program implementation and implementation supports are presented in Table 4. Correlation analysis of the 11 measures of implementation quality shows that each measure of implementation or implementation supports is significantly correlated with at least one other measure at or above *r* = +/−0.2. In addition, none of these correlation coefficients are high (<0.6), which suggest that measures of the same construct (dosage, adherence, and responsiveness) explain some but not all variation among these constructs (see Supplementary Table 1).

**Table 4:** Descriptive statistics of implementation variables.

	*n*	*n* Missing	Missing%	Mean	*SD*	*Min*	*Max*	*ICC Coach*
Teacher’s responsiveness to cycles	144	7	4.6	3.3	0.4	2.1	4	0.11
Consultancy worth	144	7	4.6	3.5	0.4	1.9	4	0.06
Words in prompt responses	145	6	4.0	64.6	30	20.9	210	0.09
Prompt access time elapsed	145	6	4.0	7.1	7.4	0.0	61	0.27
Teacher’s responsiveness to training	147	4	2.6	4.6	0.4	3.2	5	0.10
Teacher alliance	147	4	2.6	3.4	0.6	0.9	4	0.26
Couching cycles completed	145	6	4.0	7.4	1.3	1.0	8	0.25
Time in conferences	145	6	4.0	30.9	8.3	16.2	54	0.62
Attendance to training	146	5	3.3	5.4	0.9	2.0	6	0.05
Amount of program activities implemented in classroom	142	9	6.0	28.9	13.4	3.0	87	0.22
Adherence to program activities	144	7	4.6	2.4	0.4	1.0	3.5	0.31

ICC Coach, Intraclass correlation of teachers nested within coaches.

##### 2.3.2.1. Program implementation

Dosage or exposure to the seven units of the 4Rs curriculum was measured through teachers’ report of the number of units they implemented in their classroom. Exposure to a curricular unit was defined as the implementation of an entire unit consisting of at least three activities (one read aloud of the target book, one lesson activity, and one additional activity). Units with less than three activities implemented were considered incomplete. Overall, 72% of teachers completed unit 1; 80%, unit 2; 73%, unit 3; 62%, unit 4; 41%, unit 5; 31% unit 6; and 18%, unit 7. The proportion of units implemented in the classroom out of the seven total possible units in the curriculum was considered an indicator of exposure to program units (see Supplementary Tables 2, 3).

Adherence to program implementation was measured through coaches’ reports of teachers’ adherence to program activities. Coaches watched videos of their teachers’ implementation of activities in the classroom and evaluated teachers’ implementation of the action plan agreed on during the coach-teacher conference by rating a one item-sentence: “There was evidence in this video that the teacher implemented the action plan from the last cycle.” This item was rated using a 1 to 4 scale where 1 = no; 2 = Parts of the plan; 3 = Yes, the whole plan; 4 = Not Applicable. For cohort 1, coaches rated this item at the end of each of the eight cycles, whereas for cohort 2, coaches rated teachers’ videos only following cycles two and six. To be consistent with the measure of teachers’ adherence across cohorts, only ratings for cycle two (*M* = 2.29, *SD* = 0.51) and cycle six (*M* = 2.45, *SD* = 0.53) were considered in both cohorts and then aggregated to create one average score of Adherence to program implementation.

##### 2.3.2.2. Implementation supports

Teacher’s responsiveness to coaching cycles was assessed with a 4-item measure. Specifically, following coaching cycles two and six, coaches rated teachers on a 4-point scale (1 = *Strongly Disagree; 2 = Disagree*; 3 = *Agree; and* 4 = *Strongly Agree*) for the following items: “The teacher’s responses to questions were in-depth and detailed” and “Based on the teacher’s responses, she/he appears engaged in the prompt process.” Cronbach’s alphas for this scale were 0.83 for cycle two, 0.81 for cycle six, and 0.82 for the aggregate of cycles two and six. A composite score with the aggregates of the two cycles was used to represent a teacher’s responsiveness to cycles.

Teachers’ ratings of the worth of their coach’s consultation (consultancy worth) were collected during cycles two and six using a 9-item scale that assessed their satisfaction with various components of the coaching process, including web resources, coaching consultation, and productivity. Using a 4-point response range (1 = *Strongly Disagree; 2 = Disagree*; 3 = *Agree;* and 4 = *Strongly Agree*), teachers rated items such as “This meeting helped me identify specific strategies that I can use in my classroom” and “These prompts focused on issues that were relevant to my practice.” Internal consistency for cycles two and six were high (α = 0.90 and 0.91, respectively). A single score of consultancy worth was created with the average of the nine items aggregated across cycles. The decision to measure teacher’s responsiveness to coaching cycles and consultation worth in only two of the eight cycles was driven by efforts to minimize the burden teachers might experience during the evaluation of implementation supports.

The number of words teachers used in their responses to coach prompts was used as a proxy for the time and effort teachers invested in their own professional development through the 4Rs + MTP program (Downer et al., 2009b). The total “words in the prompt responses” were summed within cycles and then averaged across the cycles completed for each teacher. In addition, the time elapsed between the moment the coach posted the prompt and the moment the teacher accessed it was used as a proxy for a teacher’s interest and engagement with the coaching process. This ‘prompt access time elapsed’ was averaged across cycles completed for each teacher.

Teachers’ responsiveness to in-person training sessions was evaluated using teachers’ ratings of 20 items across four domains: trainer’s knowledge, training learning environment, organization and materials, and learning outcomes of the training session. Pearson correlations among domains ranged between 0.72 and 0.94 across the 6 days. Cronbach’s alphas for the 20 items ranged between 0.86 and 0.98 across the six training days. Given the high correlation between domains and reliability of the 20 items in general, items were averaged within and then across the six training sessions to create an aggregate score reflecting teachers’ responsiveness to training.

Teacher-coach working alliance was measured at the end of the coaching cycles using coach ratings of 34 items from the Measure of Coach and Teacher Alliance–Coach Report (Bradshaw et al., 2009). This scale measures five domains: Working Relationship, Coaching Process, Investment, Benefits of Coaching, and Barriers to Coaching. Scores were averaged across domains to create a global teacher-coach working alliance score with an overall Cronbach’s alpha of 0.96.

Four measures of dosage or exposure to implementation supports were considered: (1) number of coaching cycles completed by teacher, (2) time teacher spent in conferences with their coach, (3) teacher attendance at the training, and (4) time teacher spent visiting the 4Rs + MTP intervention website. The number of coaching cycles completed by teacher (‘Coaching cycles completed’) during the intervention was determined by teachers responding to their coaches’ prompts. Coaches and teachers also reported on their contacts using the web platform, noting the total time spent during each conference. Reports of time spent in the conference were averaged across completed cycles (*M*_coaches_ = 28.54 min, *SD* = 7.77; *M*_teachers_ = 34.15 min, *SD* = 12.26) and then one score with averages of coach and teacher reports was created as an indicator of “teachers” time spent in conference’ with coaches. A measure of teacher attendance in training was computed as the number of days a teacher attended in-person sessions of training out of the 6 days total days of training that were provided. Finally, to evaluate teacher exposure to teaching resources available through the 4Rs + MTP intervention website, the web platform captured information about the amount of time spent by teachers visiting the library page with text and video examples of high-quality teacher practices and visiting their confidential consultancy page where teachers could watch their teaching practices as edited by their coach. From this web usage data, the total amount of “time spent visiting the website” was calculated as an indicator of web-based exposure to the intervention. A cut-off maximum of 15 mins per page visit was used to correct for the times teachers ended their web session but forgot to logout.

#### 2.3.3. Covariates

##### 2.3.3.1. Professional burnout

Teacher burnout was assessed using the emotional exhaustion and personal accomplishment subscales of the Maslach Burnout Inventory-Educator Survey (MBI-ES; Maslach et al., 1986, 2001). The depersonalization subscale was not included as it has shown poorer internal consistency when compared to the other two scales (Schaufeli et al., 2001); therefore, it was excluded from the survey to reduce survey length. The emotional exhaustion subscale included nine items (e.g., “I feel emotionally drained from my work”) and the personal accomplishment subscale included eight items (e.g., “I feel exhilarated after working closely with my students”). Teachers were instructed to report the frequency with which they experienced the job-related stressors using a 7-point Likert scale ranging from 0 (“never”) to 6 (“every day”). Emotional exhaustion and personal accomplishment both showed acceptable internal consistency with Cronbach’s alpha values of 0.92 and 0.72, respectively.

##### 2.3.3.2. Depression, anxiety, and stress

The Depression, Anxiety, and Stress Scale—Short Form (DASS-21; Lovibond and Lovibond, 1995), is a self-report measure that assesses symptomatology of depression, anxiety, and stress among adults. Each of the three subscales contains seven items. Teachers rated the degree to which given statements applied to them over the past week on a 4-point Likert-type scale ranging from 1 (Did not apply to me at all) to 4 (Applied to me very much, or most of the time). Sample items include “I feel that I had nothing to look forward to” (depression), “I was worried about situations in which I might panic and make a fool of myself” (anxiety), and “I found it hard to wind down” (stress). In the current study, the three subscales were moderate to strongly correlated (*r* = 0.47–0.67) and there was strong internal consistency among the items (Cronbach’s alpha = 0.90), therefore, the DASS-21 total score was used. Prior to conducting analyses, the DASS-21 total score was transformed, using the natural logarithm to base 10, to reduce the level of skewness and kurtosis. Before transformation, the mean DASS-21 score was 0.24 (*SD* = 0.30) and after transformation the mean DASS-21 score was 0.08 (*SD* = 0.09).

##### 2.3.3.3. Teacher psychological wellbeing

The Psychological Well-Being Scale is a self-report measure that assesses teachers’ autonomy, personal growth, and positive relations with others (Ryff and Keyes, 1995). Each scale contains 7 items. Teachers rated the degree to which they agree with personal statements on a 7-point Likert-type scale ranging from 1 (strongly agree) to 7 (strongly disagree). Sample items included: “I tend to be influenced by people with strong opinions” (autonomy), “I am not interested in activities that will expand my horizons” (personal growth), and “Most people see me as loving and affectionate” (positive relations with others). The internal consistency for the total scale was moderate to high (Cronbach’s alpha = 0.72), therefore the total score based on an average of the 21 items was used for analysis in this study.

##### 2.3.3.4. Positive and negative affect scale

The Positive and Negative Affect Scale (PANAS; Watson et al., 1988) consists of 10 words that describe positive and negative emotions. Teachers read each word and indicated the extent to which they had felt that way during the past few weeks on a 5-point Likert type scale ranging from 1 (Very Slightly or Not at All) to 5 (Extremely). Sample items of the subscales included: “Enthusiastic” (positive affect) and “Irritable” (negative affect). Internal consistencies were high for both positive affect (Cronbach’s alpha = 0.89) and negative affect (Cronbach’s alpha = 0.83).

Additional teacher-reported teacher and classroom demographic characteristics were considered as covariates, including teachers’ race/ethnicity, teachers’ years of experience, classroom type (e.g., special education, ICT/CTT), class size, proportion of students in the classroom with Individualized Education Plans, proportion of students in the classroom with Limited English Proficiency, proportion of female students in the classroom, and proportion of behaviorally at-risk students in classroom (i.e., students above norm cut-off scores on aggression and/or conduct problems were coded as 1). Cohort (Cohort 1 = 0; Cohort 2 = 1) was included as a covariate in all analyses.

## 3. Results

### 3.1. Missing data

Percentage of missing data on implementation variables was low, ranging between 2.6 and 6%. Table 4 shows descriptive statistics of quality of implementation variables and percentage of missing observations per variable. Several child level and teacher-classroom level variables showed more than or close to 10% of missing data. At the child level, Math (56%) and ELA score (57%) at wave 1 showed the highest proportion of missing data, whereas teacher burnout (9.7%) and teacher negative affect (9.4%), showed the highest proportion at the teacher/classroom level. Tables 5, 6 show descriptive information and proportion of missing observations of child outcomes and teacher-classroom variables, respectively. To examine whether data were missing completely at random (MCAR), tests for child level and teacher-classroom level variables at both wave 1 and wave 2 were performed using the function TestMCARNormality from the R package MissMech (Jamshidian et al., 2014). An additional test to detect missing patterns (Little, 1988) was also conducted on both datasets using the function LittleMCAR from the R package BaylorEdPsych (Beaujean and Beaujean, 2012).

**Table 5:** Descriptive statistics of child outcomes.

	*n*	Miss%	Mean	*SD*	*Min*	*Max*	*ICC* teacher	*ICC* school
Hostile attribution bias	4,092	19.5	0.37	0.33	0	1	0.06	0.02
T1 hostile attribution bias	4,435	12.7	0.36	0.31	0	1	0.06	0.02
Aggressive interpersonal strategies	4,089	19.5	0.21	0.32	0	1	0.09	0.03
T1 aggressive interpersonal strategies	4,432	12.8	0.17	0.29	0	1	0.08	0.02
Internalizing symptoms	4,055	20.2	0.42	0.22	0	1	0.06	0.02
T1 internalizing symptoms	4,407	13.3	0.43	0.21	0	1	0.06	0.02
Aggressive behavior child report	4,085	19.6	0.53	0.61	0	3	0.15	0.07
T1 aggressive behavior child report	4,427	12.9	0.47	0.58	0	3.3	0.12	0.05
Aggressive behavior teacher report	4,469	12.0	1.44	0.53	1	4	0.17	0.04
T1 aggressive behavior teacher report	4,718	7.1	1.41	0.53	1	4	0.18	0.06
Social competence	4,469	12.0	2.99	0.75	1	4	0.26	0.05
T1 social competence	4,718	7.1	2.92	0.74	1	4	0.24	0.07
ELA score	4,616	9.2	298.13	34.31	168	408	0.34	0.15
T1 ELA score	2,175	57.2	293.53	35.35	163	398	0.37	0.10
Math score	4,684	7.8	290.85	38.85	165	397	0.34	0.19
T1 math score	2,213	56.4	291.54	36.82	176	401	0.33	0.18
School absences	5,044	0.7	11.53	11.12	0	102	0.07	0.03
T1 school absences	4,854	4.5	12.31	11.63	0	97	0.08	0.05

T1, Time 1-baseline measure; ICC Teacher, Intraclass correlations with teacher and school ID as cluster variables.

**Table 6:** Descriptive statistics of classroom and teacher variables.

	*n*	Missing %	Mean	*SD*	*Min*	*Max*
Emotional support	314	4.8	4.30	0.91	2.0	7.0
T1 emotional support	320	3.0	4.52	0.82	2.2	6.5
Instructional support	314	4.8	3.32	0.87	1.0	5.5
T1 instructional support	320	3.0	3.55	0.80	1.4	6.25
Classroom organization	314	4.8	5.94	0.74	3.3	7.0
T1 classroom organization	320	3.0	5.89	0.70	2.8	7.0
Proportion student at risk	318	3.6	0.20	0.19	0.0	0.9
Number of students	321	2.7	22.36	5.92	6.0	33.0
Year of experience	322	2.4	10.71	7.56	1.0	40.0
Proportion of students with IEP	321	2.7	0.15	0.23	0.0	1.0
Teacher burnout	298	9.7	1.55	0.86	0.0	4.3
Teacher psychological wellbeing	301	8.8	5.98	0.55	3.9	7.0
Teacher positive affect	302	8.5	3.97	0.69	1.6	5.0
Teacher negative affect	299	9.4	1.68	0.62	1.0	4.4
Teacher depression, stress, and anxiety	301	8.8	0.25	0.29	0.0	1.9

T1, Time 1-baseline measure.

Intraclass correlations of student variables ranged between 0.06 and 0.37, and between 0.02 and 0.19 when clustered by teacher ID and by school ID, respectively (see Intraclass Correlations, ICC, Table 5). Since shared variance of children’s outcomes clustered by school were low (McCoach and Adelson, 2010), only teacher identification number was included as a cluster variable in the models for this study. The dataset including the full sample of teachers and students was imputed using the R package multiple imputation with multivariate imputation by chained equation (MICE; Zhang, 2016). Variables at level 2 (teacher/classroom) were imputed using the function 2only.pmm that aggregates level-1 predictors and imputes the level-2 variables using predictive mean matching (pmm; Kleinke, 2017). Variables at level 1 (child level) were imputed using random forest (Shah et al., 2014). Each imputation was performed separately for treatment and control groups and then combined into a single dataset. Twenty imputed datasets were used for analyses. Results of analysis of missing patterns and a description of multiple imputation procedures is presented in Supplementary Data Sheet 1.

### 3.2. Main analyses

#### 3.2.1. Identifying profiles of quality of implementation among teachers

The first aim of this study was to identify teacher compliance propensity by exploring which components of program implementation and implementation supports would discriminate between profiles of quality of implementation for teachers in the treatment group (see Figure 1A). Latent Profile Analyses (LPA) was used to estimate the probability of teachers belonging to different profiles (clusters; Oberski, 2016) of quality of implementation. The LPA was estimated using the array of assessments of program implementation and implementation support (described in section Measures). Implementation variables were standardized. Intraclass correlations (ICC) of implementation variables suggested that part of the variances could be attributed to the nested structured of the data (i.e., teachers nested within coaches; *ICC*, *M =* 0.21, Min *=* 0.54, Max = 0.62; see Table 4). To account for the effect of coaches, dummy codes for coach were included in the subsequent LPA.

**Figure 3:**
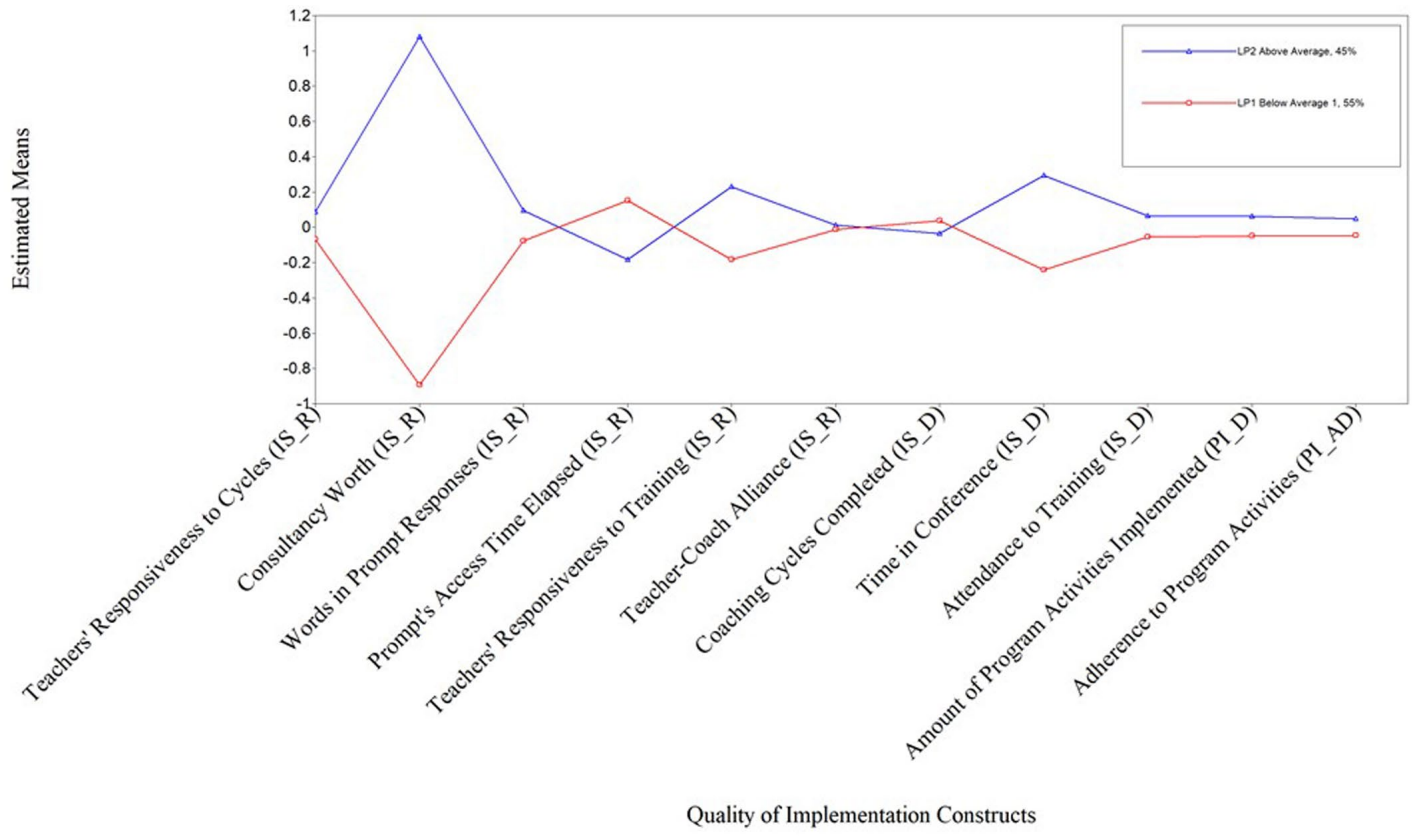
Estimated means of quality of implementation constructs by latent profile IS_R, Implementation Support Responsiveness; IS_D, Implementation Support Dosage; PI_D, Program Implementation Dosage; PI_AD, Program Implementation Adherence.

LPA analysis was performed in Mplus using maximum likelihood estimation to handle missing data (Muthén, 2004). The model of two profiles of implementation showed a good fit (*Loglikelihood* = −2077.53, *AIC* = 4237.07, *BIC* = 4359.67, *Entropy* = 0.99). Of the 147 teachers included in the analysis (i.e., four teachers who dropped out at the beginning of the study were excluded from this analysis), 81(55%) were members of latent profile one (LP1, below average) and 66 (45%) were members of latent profile two (LP2, above average). The probability of being in LP1 was significantly predicted by two implementation variables: consultancy worth (*B* = −0.89, SE = 0.03, *p* < 0.001) and time in conferences (*B* = −0.24, SE = 0.11, *p* < 0.025); whereas four variables significantly predicted the probability of being in LP2: consultancy worth (*B* = 1.08, SE = 0.001, *p* < 0.001), time in conference (*B* = 0.29, SE = 0.120, p= 0.015), prompt access time elapsed (*B* = −0.183, SE = 0.079, *p* = 0.020), and teacher responsiveness to training (*B* = 0.23, SE = 0.11, *p* =. 040). Figure 3 shows the standardized coefficients for each of the implementation constructs for each of the LPs.

Validation analyses of the LPs suggested differences in four of 11 implementation constructs for teachers classified in LP1 and LP2 groups, as indicated by the Welch Two Sample *t*-test (Welch, 1947; see Table 7). Specifically, compared to teachers in the LP2 (above average) group, teachers in LP1 (below average) had significantly lower responsiveness to training and ratings of consultancy worth, spent significantly less time in conferences with their coaches, and higher prompt access time elapsed (i.e., spent more time to access the prompts provided by their coaches). None of the differences between LP1 and LP2 on the other five variables of implementation support and the two variables from program implementation were significant.

**Table 7:** Mean differences of quality of implementation constructs by latent profile.

	LP1 Below average	LP2 above average			
Implementation support	Mean (*SD*)	Mean (*SD*)	*t*	*df*	*p*
Teacher’s responsiveness to cycles	3.27 (0.36)	3.34 (0.41)	−1.13	128.23	0.259
Consultancy worth	3.15 (0.22)	3.82 (0.17)	−20.86	141.66	0.001
Words in prompt responses	62.31 (30.33)	67.33 (29.56)	−1.01	139.61	0.316
Prompt’s access time elapsed	8.24 (8.87)	5.77 (4.79)	2.13	123.82	0.035
Teacher’s responsiveness to training	4.51 (0.35)	4.68 (0.34)	−2.84	140.44	0.005
Teacher alliance	3.34 (0.65)	3.39 (0.49)	−0.52	144.31	0.606
Couching cycles completed	7.47 (1.22)	7.33 (1.40)	0.61	130.10	0.540
Time in conferences	28.87 (7.93)	33.42 (8.07)	−3.41	137.56	0.001
Attendance to training	5.37 (1.03)	5.49 (0.81)	−0.80	143.96	0.425
**Program implementation**					
Amount of program activities implemented in classroom	28.26 (12.52)	29.75 (14.36)	−0.65	126.02	0.514
Adherence to program activities	2.34 (0.44)	2.38 (0.44)	−0.56	136.02	0.574

Results of the LPA, validation analysis, and visual inspection of the graph of standardized coefficients by LPs suggest that LP1 reflects a profile of teachers with below average quality of implementation and LP2 reflects a profile of teachers with above average quality of implementation (see Figure 3). Differences between these two profiles can be grouped as differences in teacher responsiveness to implementation supports and the dosage of implementation support they received, with teachers in the below average profile being significantly less responsive and receiving less support than teachers in the above average profile. None of the program implementation variables significantly characterized the profiles of quality of implementation, but this result should be interpreted with caution as explained later in discussion and limitations. The four teachers who dropped the intervention and therefore did not implement the program or receive implementation support were manually assigned to LP1 (below average profile).

#### 3.2.2. Estimating teacher compliance propensity using teacher and classroom covariates

To further address our first study aim, we then used Follmann’s (2000) propensity score approach to explore teacher and classroom covariates that predicted teacher compliance propensity for teachers in the treatment group (see Figure 1B and the full sample treatment and control groups; see Figure 1C). Compliance propensity for the treated sample, that is, the propensity to be in the above average implementation latent profile (LP1 = 0; LP2 = 1) was estimated using nine baseline measures of classroom and teacher characteristics known to predict quality of implementation. These measures include proportion of students at behavioral risk, number of students in classroom, proportion of students with active IEPs, teachers’ number of years of experience, teacher burnout, teacher psychological wellbeing, teacher positive affect, teacher negative affect, and teacher score of depression, anxiety, and stress (aggregated). Analysis was performed using Random Forest, a machine learning technique (Zhao et al., 2016) robust to non-normal data which performs well with multivariate data of different formats (continuous and categorical). Random Forest uses recursive partitioning, which applied to propensity score estimation in this study consists of recurrently splitting the data into nodes (i.e., groups) based on values of the categories of a categorical covariate or a cutoff applied to a continuous covariate that discriminates between quality of implementation profiles (LP1 and LP2). Propensity scores can be obtained as the proportion of cases in the above average quality of implementation profile at each terminal node. Random Forest was implemented using the cforest unbiased function from the R package “Party” (V1.3-5; Hothorn et al., 2006). Bias was controlled by running a large number of trees with bootstrapped samples of the same size as the original sample and combining results, options were set to 1,000 trees and a random sample of *m* = 3 predictors, chosen from all possible predictors *p* = 9 using the formula *m =* $\sqrt{p}$. Compliance propensity was estimated for each imputed dataset and then aggregated across datasets to create an average compliance propensity score (Mean compliance = 0.43; SD = 0.11; Skewness = 0.24; Kurtosis = −0.64). The distribution of compliance propensities was similar across datasets. Density plots of compliance propensity across 20 imputed datasets can be found in Supplementary Figure 1. Not all nine baseline classroom and teacher characteristics contributed equally to the estimation of compliance propensity as suggested by coefficients of mean decrease difference in accuracy (MDD). Covariate coefficients of mean decrease of accuracy greater than zero indicate that the absence of such covariates have an impact in decreasing the accuracy of the model (Louppe et al., 2013). MDD was computed using the function varimp and allowing association with covariates with a threshold of 0.2, which implies that the resulting predictor importance score is conditional on the importance of other predictors similar to beta coefficients in regression models (Strobl et al., 2008). According to this indicator, number of years of experience as a teacher was the most important covariate predicting compliance propensity, with the highest mean decrease in accuracy coefficient (*MDD* = 0.015), followed by teacher burnout (MDD = 0.052). The other seven covariates have MDD coefficients lower than 0, suggesting low to zero contribution to the model of classification (see Supplementary Table 4A). Further analysis in the treated sample show that teacher compliance propensity significantly predicted three variables related to implementation supports: teacher’s ratings of consultancy worth (*B* = 0.68; *SE* = 0.51), average time in conference (*B* = 0.23; *SE* = 0.70), and teacher’s responsiveness to training (*B* = 0.24; *SE* = 0.69) These findings suggest the resulting propensity scores estimated by years of experience and teacher’s levels of burnout, are significantly associated with variables of implementation supports that characterized the above and below profiles of quality of implementation (see Supplementary Table 4B). Compliance propensity for teachers in the control group was predicted using the weights of the nine covariates based on the propensity estimation conducted for teachers in the treatment group. Visual inspection of box and whisker plots suggested an adequate area of common support, that is, the area of the distribution of compliance propensity includes values for teachers in the treatment and control group. To evaluate covariate balance across treatment and control conditions, analysis of covariance (ANCOVA) was used to examine differences on each covariate between treatment and control groups at above and below average quality of implementation after controlling for compliance propensity. Results showed that interactions between random assignment and levels of above and below average compliance propensity were not significant for any of the covariates used to predict profiles of quality of implementation, suggesting balance was achieved for all covariates across the randomly assigned conditions (see Supplementary Table 5).

#### 3.2.3. Relations among school random assignment to 4Rs + MTP, teacher compliance propensity, quality of classroom interactions, and child outcomes

Our second aim was to examine teacher compliance propensity as a moderator of the relationship between school random assignment to 4Rs + MTP and (a) quality of classroom interaction (see Figure 2, paths P2 and P4), (b) children’s academic and SEL outcomes (see Figure 2, paths P1 and P5), and (c) children’s academic and SEL outcomes as mediated by the quality of classroom interactions (see Figure 2, paths P2, P4, and P3).

Here we used multilevel modeling to test the relations between random assignment (a level-2 predictor) and child academic and SEL outcomes (level-1 outcomes), mediated by quality of classroom interaction (a level 2 variable). This multilevel mediation is examined at different levels of teacher compliance propensity (a level-2 moderator), using an adaptation of the general path analytic framework proposed by Edwards and Lambert (2007) for testing direct, indirect, and total effects on an outcome at different levels of a moderator. Although Edwards and Lambert (2007) do not discuss the moderated mediation in a multilevel path, to account for the nested structure of students within teachers-classrooms in this study, a random intercept is also included in the regression equations (Tingley et al., 2014; Rockwood, 2017; Finch, 2022).

The multilevel moderated mediation model was fit using the SemTools R package (Jorgensen et al., 2019), which allows the estimation of multilevel analyses in multiple imputed datasets using the structural equation models (SEM.mi) function. Inspection of correlations among residuals (higher than 0.1) and modification indices suggest covariation between domains of quality of classroom interaction, therefore covariances among emotional support, classroom organization, and instructional support were included in the model. Teacher ID was used as a cluster variable. The model converged in the 20 imputed datasets and results from the pooled fit measures showed an adequate fit $x^{2}$ (134) = 386.69 *p* < 0.01, CFI = 0.91, TLI = 0.84, RMSEA = 0.02. Rubin’s (1987) rules were used to pool point and *SE* estimates across 20 imputed data sets, and to calculate degrees of freedom for each parameter’s *z-*test and 95% confidence interval (*CI*). Coefficients were tested with an α=0.05, two-tailed level of significance. However, trends in the hypothesized direction are reported using one-tailed tests (90% *CI*). To test direct and indirect effects, robust confidence intervals were estimated using a Monte Carlo test of mediation (MacKinnon et al., 2004) with 1,000 random samples with population values equal to the coefficients and covariance of the sample (Preacher and Selig, 2012). Quality of classroom interaction and child outcomes were regressed on school treatment assignment (0 = control, 1 = 4Rs + MTP), compliance propensity, the interaction of treatment assignment and compliance, the three domains of quality of classroom interaction, cohort (0 = cohort 1; 1 = cohort 2), and values of the target outcome at wave 1 (grand mean centered for classroom outcomes, and group mean centered for child outcomes). Each path of the moderated mediation was tested at two levels of the moderator, teachers’ compliance propensity-mean centered, namely, 1SD below average compliance and 1SD above average compliance. An example equation with a detailed explanation can be found in Supplementary Data Sheet 2.

##### 3.2.3.1. Moderation of compliance propensity on the effects of 4Rs + MTP on quality of classroom interactions

Random assignment to 4Rs + MTP was associated at the trend level (*p* < 0.10) with positive effects on emotional support when moderated by above average compliance (*b* = 0.25, SE = 0.15, 95% CI [−0.035, 0.535]). Since the effect of treatment on emotional support followed the hypothesized direction, this direct path was tested at the 90% CI using the Monte Carlo method for mediation with 1,000 replications. Results show that the conditional effect of treatment on emotional support was significant at the 90% CI [0.0208, 0.4736]. By contrast, when evaluated at below average level of compliance, the effect of treatment on emotional support was not significant and close to zero (*b* = 0.04, SE = 0.12, 95% CI [−0.193, 0.278]). The effects of treatment on instructional support and classroom organization at below average compliance were also not significant. Table 8 shows parameters and test statistics of the direct effects of treatment on all three domains of classroom interactions, conditioned on different levels of the moderator compliance (path “a”). Parameter estimates and test statistics for each predictor in the model can be found in Supplementary Table 6.

**Table 8:** Effects of 4Rs + MTP on quality of classroom interactions conditioned on teacher compliance propensity (Path “a”).

Paths	Teacher compliance propensity											
	Below average						Above average					
	*b*	*SE*	*B*	*Z*	95% CI		*b*	*SE*	*B*	*Z*	95% CI	
					Lower	Upper					Lower	Upper
TX → ES	0.04	0.12	0.01	0.36	−0.193	0.278	0.25	0.15	0.17	0.17*t*	−0.035	0.535
TX → IS	−0.10	0.13	−0.07	−0.77	−0.343	0.150	0.01	0.14	0.02	0.02	−0.273	0.278
TX → CO	−0.14	0.11	−0.11	−1.29	−0.346	0.071	−0.06	0.12	−0.03	−0.51	−0.288	0.170

TX, Random assignment to 4Rs + MTP (1) vs. Control (0); ES, Emotional Support; IS, Instructional Support; CO, Classroom Organization.

##### 3.2.3.2. Moderation of compliance propensity on the effects of 4Rs + MTP on children’s academic and SEL outcomes

Teacher compliance propensity moderated the effect of treatment on children’s school absences. Random assignment to 4Rs + MTP was associated with significantly fewer school absences than random assignment to the control group when moderated by above average teachers’ compliance propensity (*b* = −0.084, SE = 0.04, 95% Monte Carlo CI: [−0.13, −0.012]). Association of random assignment to 4Rs + MTP and school absences was not significant when tested at levels below average compliance (*b* = 0.03, SE = 0.039, 95% CI [−0.045, 0.107]). Compliance propensity did not moderate the effects of treatment on the other seven children’s outcomes. Coefficients and test statistics for each predictor can be found in Supplementary Table 7. See Supplementary Table 8 for parameters and test statistics of the direct effects of treatment on all child outcomes, conditioned on different levels of the moderator compliance (path “c”).

##### 3.2.3.3. Moderation of compliance propensity on the effects of 4Rs + MTP on children’s academic and SEL outcomes, as mediated by quality of classroom interactions

Results of the effects of domains of quality of classroom interactions on children’s academic and SEL outcomes showed a significant positive effect of classroom organization on children’s academic test scores (*b* = 4.61, SE = 1.857, 95% CI: [0.013, 9.64]). In addition, there was also a trend level positive effect of instructional support on academic test scores (*b* = 2.90, SE = 1.69, 95% CI: [0.086, −0.411], *p* = 0.08). Although not significant, there were also trend level effects of quality of classroom interactions on child reported aggressive behavior. Higher emotional support was associated with lower aggressive behavior (*b* = −0.03, SE = 0.02, 95% CI: [−0.063, 0.004], *p* = 0.08). Likewise, higher classroom organization was associated with lower child reported aggressive behavior (*b* = −0.03, SE = 0.02, 95% CI: [−0.065, 0.005], *p* = 0.09). Finally, there were also trend level associations in the expected direction between classroom organization and children’s aggressive interpersonal strategies (*b* = −0.02, SE = 0.01, 95% CI: [−0.032, 0.002], *p* = 0.08) and internalizing symptoms (*b* = −0.01, SE = 0.01, 95% CI: [−0.002, 0.002], *p* = 0.09).

**Figure 4:**
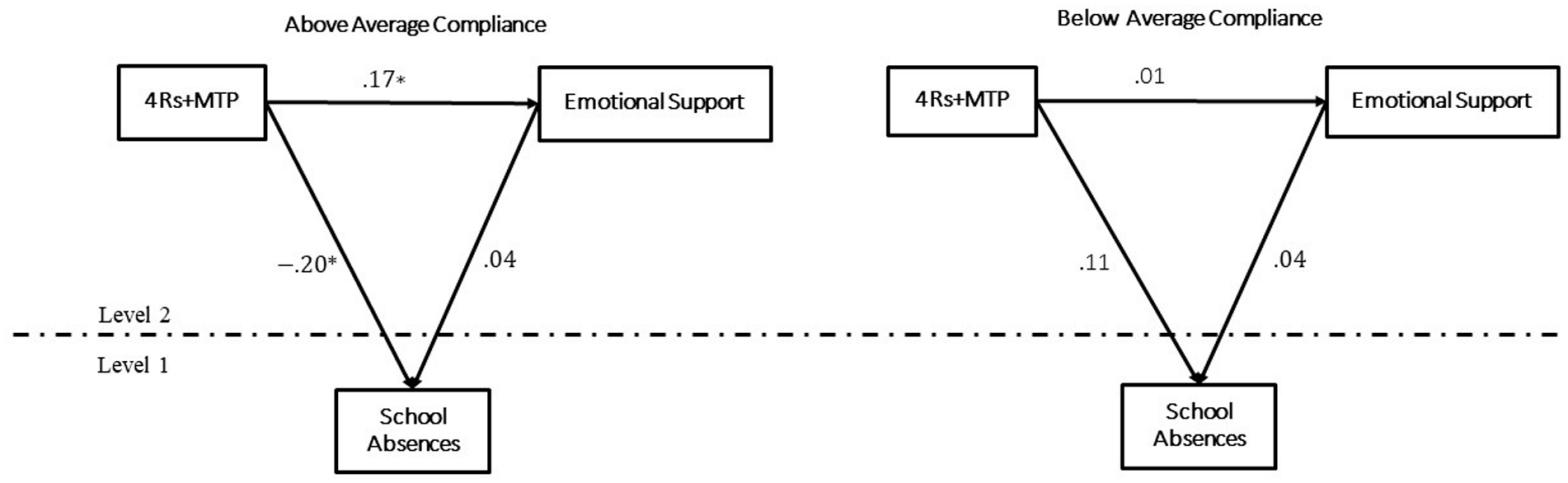
Mediation of quality of classroom interactions in the effect of random assignment to 4Rs + MTP on child school absences at different levels of teacher compliance propensity.

None of the domains of quality of classroom interactions mediated the effect of treatment status on child outcomes. However, the total effect of treatment on school absences was significant when evaluated at levels of above average compliance (*b* = −0.07, SE = 0.036, 95% CI: [−0.1.44, −0.003]; see Figure 4; Supplementary Tables 7, 8).

## 4. Discussion

Evidence for evaluation of SEL school interventions shows that promoting children’s social and emotional skills in schools can have important positive effects on children’s outcomes, including academic achievement, reduced school absences, and reduced problem behaviors (Jones et al., 2019; Weissberg, 2019); and on classroom and school outcomes, such as improvements in school climate and more supportive relationships in classroom interactions (Pianta et al., 2008; Brown et al., 2010; Hamre et al., 2013). However, studies testing the role of classroom interactions as mediators of the effects of SEL programs on children’s outcomes are scarce. In addition, evidence regarding the implementation of SEL programs suggests programs impacts are amplified when programs are implemented with high quality (Dane and Schneider, 1998; Durlak, 2016). Few studies of SEL programs to date include implementation variables as predictors in their program impact models (Derzon et al., 2005; Humphrey et al., 2018) or use holistic measures of quality of implementation instead of relying solely on measures of dosage (Durlak and DuPre, 2008; Domitrovich et al., 2010).

Accordingly, this study had two primary aims. First, we explored several indicators of teachers’ adherence to and dosage of 4Rs + MTP program activities, and responsiveness and exposure to program implementation supports to identify teachers’ profiles of quality of implementation. We then estimated teachers’ propensity to comply with implementation based on teacher personal characteristics and classroom characteristics known to predict quality of implementation. Second, we examined the effects of the 4Rs + MTP program on classroom quality of interactions and children’s academic and SEL outcomes at different levels of teachers’ compliance propensity, including examining whether classroom quality of interactions mediated the effect of the 4Rs + MTP program on children’s SEL and academic outcomes, when moderated by levels of teachers’ compliance propensity.

This is the first study to date to take a compliance propensity approach to understanding whether and how quality of implementation moderates the effects of an SEL program on classroom and child outcomes, and as such it is somewhat exploratory in nature. It is important to state that the compliance propensity approach to examining quality of program implementation may lack precision relative to the use of direct indicators of treatment teachers’ implementation quality. However, the strength of this approach is that it affords inclusion of teachers in the control group for whom there were no direct measures of implementation, thus reducing the corresponding bias when making causal inferences about treatment effects.

### 4.1. Profiles of teachers’ quality of implementation and compliance propensity

Teachers’ quality of implementation of the 4Rs + MTP program was represented by two profiles consisting of teachers with below and above average quality of implementation. Teachers in the above average quality of implementation profile were characterized by their high responsiveness and their high exposure to implementation supports; whereas teachers in the below average quality of implementation profile were characterized by their low responsiveness and low exposure to implementation supports.

Traditionally, research on implementation has distinguished between high and low quality of implementation using measures of program implementation, such as dosage and adherence to program implementation (Dusenbury et al., 2004). Findings in the current study suggest that the distinction between high and low quality of implementation might also be characterized by measures of teachers’ responsiveness and dosage of implementation supports. These findings support claims from researchers in implementation science suggesting the importance of including measures of dosage and responsiveness to implementation supports, such as time spent with coaches, teacher’s ratings of consultancy worth and teacher responsiveness to training, also documented in the literature of quality of implementation as central to program impact (Domitrovich et al., 2010; Wehby et al., 2012; Pas et al., 2015).

Profiles of quality of implementation were established based on distinguishable patterns in the implementation supports teachers received through the 4Rs + MTP program. However, measures of dosage and adherence to program implementation did not play a significant role in the characterization of teacher’s profiles of quality of implementation in this study. These results may be seen as contradictory with past literature about the positive associations between the quality and quantity of coaching and training teachers received and teacher’s dosage of and adherence to program implementation (Downer et al., 2009a,b; Wehby et al., 2012; Patti et al., 2015; Ashworth et al., 2018). In accordance with this literature, it was expected that both measures of implementation supports and program implementation would significantly discriminate between above and below teacher’s profiles of quality of implementation. However, it is worth noting that, as described in our methods, measures of dosage of program implementation were provided by teacher’s self-reports of the number of activities they implemented in their classrooms. Previous findings show that teacher’s self-reports of dosage are frequently high, which compromises the ability of these kind of measures to discriminate between high and low dosage of implementation (Domitrovich et al., 2010). Further research should include observed measures of dosage in program implementation, which have been found to be more reliable than self-reported measures (Durlak and DuPre, 2008; Domitrovich et al., 2010). Regarding adherence to implementation of curricular activities in the classroom, differences on this coach reported measure might have been obscured due to the decision to control for coach assignment during the estimation of profiles, and thus minimize the potential bias of coach’s idiosyncratic judgments. Differences in teacher-coach alliance, also reported by coaches, and found to be a significant predictor of program implementation in previous research (e.g., Wehby et al., 2012) might have also been affected by the decision to control by coach assignment in this study.

### 4.2. Compliance propensity and teacher and classroom characteristics

Years of experience as a teacher and professional burnout were the sole contributors to the estimation of teacher compliance propensity. Findings suggest that more experienced teachers and teachers who reported lower levels of burnout were more likely to be high compliers in implementing the 4Rs + MTP program. Although exploratory, these results are consistent with prior evidence that shows links between variation in teacher professional experience and program implementation (Fixsen et al., 2009; Weiss et al., 2014), specifically findings that early career teachers report low quality of implementation (Domitrovich et al., 2019) and more experienced teachers spend more time in conferences with their coaches (Downer et al., 2009a). In addition, recent evidence from a randomized trial testing the integration of the Good Behavior Game and MTP among early career teachers found significant intervention effects on student behavior and achievement but only among those teachers exhibiting high baseline levels of distress and disruptive behavior in their classrooms (Tolan et al., 2020). Interestingly, in the current study, proportion of children in the classroom considered at behavioral risk was not significantly associated with teacher implementation despite prior evidence to the contrary (Musci et al., 2019).

### 4.3. Compliance propensity and relationships among 4Rs + MTP, classroom quality of interactions and children’s outcomes

There was a trend level effect in the moderation of compliance propensity on the effects of 4Rs + MTP on classroom emotional support. When examined at above average levels of compliance, 4Rs + MTP had a positive effect on classroom emotional support. This effect, however, was negligible for teachers with below average compliance propensity. Previous work has found that teachers receiving support through on-going coaching and web-based material in the MyTeachingPartner (MTP) coaching program were better at proving emotional and instructional support to their students (Pianta et al., 2008). Further, as noted above, preliminary findings from the efficacy trial of the 4Rs + MTP program show positive main effects of overall exposure to 4Rs + MTP implementation on classroom emotional support (Brown et al., 2019). Although the aims of the current study did not include the evaluation of specific components of quality of implementation on classroom quality of interactions, findings suggest the effect of the 4Rs + MTP program on emotional support is amplified among teachers who have a higher propensity to comply with the implementation, including their propensity to benefit from the coaching support provided through MTP.

Students´ social and emotional skills are effectively taught and learned within caring and supportive environments (Collaborative for Academic, Social, and Emotional Learning, 2017), where students feel secure and positively related to others (Roeser et al., 2000; Hamre and Pianta, 2007). However, teachers also need social and emotional skills to build caring and supportive relationship with their students (Kingston and Wilensky, 2018). The support teachers receive from coaches during program implementation might improve the emotional resources teachers need to provide higher quality emotional support in their classrooms. For instance, other research has found that teachers receiving ongoing coaching have reported increased self-awareness, self-management, and improved relationships with students (Patti et al., 2015). Moreover, strong teacher-coach alliance buffered the negative effects of teacher burnout on teacher implementation of SEL activities with their students (Wehby et al., 2012).

By contrast, the effects of 4Rs + MTP on instructional support and classroom organization were not moderated by teachers’ compliance propensity. More research is needed to understand the influence of variations in quality of implementation in the effect of 4Rs + MTP on instructional support and classroom organization.

Compliance propensity also moderated the effects of 4Rs + MTP on child school attendance. Specifically, when examined at above average compliance propensity, 4Rs + MTP was associated with fewer school absences. This effect, however, was negligible for children from teachers with below average compliance propensity. This study provides evidence about the role of SEL programs in improving children’s school attendance when the program is well implemented, and in this case, when teachers have high propensity to implement the program with high quality.

This contribution is relevant in the context of SEL program implementation, considering the limitation of most SEL programs with regard to having significant impacts on academic attainment (less than 10% in the United States; Grant et al., 2017). In a prior quasi-experimental study of the 4Rs + MTP program, the integration of MTP coaching for teachers with the prior 4Rs intervention model also yielded positive effects on children’s school attendance relative to the 4Rs program without MTP coaching (Doyle et al., under review).

Previous literature suggests that highly supportive classrooms are likely to encourage students’ attendance (Barth, 1984; Baker et al., 2001; McCluskey et al., 2004). However, none of the domains of classroom quality of interactions mediated the effects of 4Rs + MTP in reducing school absences, suggesting that the mechanism through which 4Rs + MTP is associated with lowering child absences at the above average levels of compliance propensity is not explained by overall higher quality classroom interactions. An alternative explanation is that teachers with high compliance propensity provide effective support to individual students, perhaps those at higher risk of truancy, without necessarily extending this support (or not extending it to the same degree) to all children in the classroom. Interventions targeting individuals or small groups at risk of truancy, instead of at the classroom as a whole, have been common practice in education (Teasley, 2004; Reid, 2013). Findings might suggest that these teachers in 4Rs + MTP would develop the skills needed to provide effective support to students at risk of truancy, resulting in their higher attendance.

Finally, domains of classroom interaction quality (i.e., emotional support, instructional support, and classroom organization) did not mediate the effects of 4Rs + MTP on any of the other child outcomes at levels of above average compliance propensity. It is possible these nonsignificant effects are a function of the mediating mechanism (i.e., domains of classroom interaction quality) not actually being evident when examined based on different levels of the moderator. Since teacher years of experience and burnout were the main predictors of compliance propensity, examining the effects of treatment at above average compliance propensity is virtually equivalent to examining the effects of treatment at high levels of teaching experience and low levels of burnout. It is possible, that experienced teachers with low levels of burnout might have developed strong skills in promoting effective classroom quality of interactions, such that treatment differences are noticeable only in one specific domain of classroom quality of interactions: emotional support.

Although, in this study there was not a significant mediation effect of emotional support on child outcomes, taken together, the evidence from previous studies and findings from the current study suggests that the quality of supports received by teachers with high compliance propensity in 4Rs + MTP would bolster or improve their emotional skills in ways that may in turn increase their ability to provide effective emotional support during their interactions with students. Thus, teachers would develop emotional skills needed to provide emotional support in their classrooms through highly supportive interactions with their coaches. More research is needed, nevertheless, to understand the effect of high-quality coaching in improving teachers’ emotional skills, and the mediating role of these skills on the relationship between SEL programs such as 4Rs + MTP and the quality of teachers’ emotional support in classrooms.

### 4.4. Limitations

One of the limitations in the current study was its sole reliance on teacher personal characteristics and classroom characteristics to estimate teacher compliance propensity with implementation, while excluding the potential effects of coach assignment to teachers. It is worth noting that coach assignment was controlled for during the identification of profiles of quality of implementation for the treated teachers. Accordingly, teachers’ compliance propensity in this study should be interpreted as teachers’ probability of complying with implementation given personal and classroom characteristics, and regardless of the particular influence of coaches in the implementation process. While the role coaches played in implementation might have influenced teachers´ program implementation, the decision to exclude coach assignment was the trade-off for being able to calculate compliance propensity for teachers in the control group for whom coach assignment was not available.

Some measures of child outcomes in 4Rs + MTP might be limited in terms of evaluating the effects of the program. For instance, academic measures rely only on test scores but are not sensitive to other indicators of academic performance and engagement such as the quality of a child’s academic work and participation in their classroom. Although the CLASS observation measure assessed information on interaction quality at the classroom level, it did not provide information about child level interaction quality, which may be a distinctly sensitive indicator of children’s classroom interaction experience. Including observational measures of child interactions with teacher and peers, such as inCLASS (Booren et al., 2012), might provide valuable information to evaluate the effects of SEL programs on child engagement during academic tasks and classroom interactions. Regarding social and emotional outcomes, while this study included validated scales and questionnaires that provide reliable results that allow comparisons with findings from other studies and that were largely proximal to the 4Rs + MTP program’s theory of change (e.g., measures of social-cognitive processes associated with aggression), other potential child social–emotional outcomes relevant to the program’s goals were not included in the current study (e.g., child empathy).

Finally, domains of classroom interaction quality (i.e., emotional support, instructional support, and classroom organization) did not mediate the effects of 4Rs + MTP on any of the child outcomes at above average levels of compliance. This study relied on data collected between winter and spring. Significant mediation effects may take more than the time elapsed during this period or even one full school year to manifest. Treatment differences in emotional support due to high compliance with treatment assignment, might not be sufficient to significantly mediate the effects of treatment on child outcomes when examined in this group of teachers. The possibilities for expanded effects to other domains of classroom interactions and a significant mediation effect of classroom emotional support on child outcomes will be examined in the subsample of third grade teachers who were followed and assessed in the fall and spring of the subsequent school year along with their new class of 3rd grade students.

### 4.5. Conclusion

Consistent with previous research, findings in this study show that more experienced teachers with low levels of burnout were more likely to comply with high quality implementation of the 4Rs + MTP program (Fixsen et al., 2009; Downer et al., 2009a; Wehby et al., 2012; Domitrovich et al., 2019; Musci et al., 2019; Weiss et al., 2021). These teachers showed better skills in providing emotional support and their students had fewer school absences than students of teachers with similar compliance propensity in the control group.

While this study only found significant moderation of high compliance propensity on the effect of 4Rs + MTP on child school attendance and a trend on teacher emotional support, it provides a steppingstone toward understanding the extent to which teachers’ propensity to comply with implementation influences the effects of SEL programs on classroom and child outcomes. This research provides evidence that implementing SEL programs by teachers with high compliance propensity, may have positive impacts on classroom emotional support, increasing the opportunities for providing nurturing and caring environments that promote children’s development and learning, and increasing school attendance. This is consistent with a developmental cascades approach (Masten and Cicchetti, 2010), prioritizing intervention with teachers as part of a developmental system that can then facilitate positive developmental changes at the child level. This study contributes to the understanding of quality of implementation of SEL programs, particularly, a compliance propensity approach highlights the importance of providing the resources teachers need to increase their propensity to implement an SEL program with high quality, which in turn, increases the likelihood of desired program impacts.

The interest in research on implementation has grown in recent years, in part due to the potential answers such research can provide policymakers in determining adequate or minimum levels of implementation needed for a program to be effective (Meyers et al., 2012; Smith and Hutchinson, 2022). Particularly, findings from the current study suggest that the implementation supports teachers receive during program implementation is a key component in securing high levels of compliance.

*Post hoc* analyses of variance of teachers’ quality of implementation in this study suggests that teacher quality of implementation likely varied systematically by coach assignment. Although measures of coach alliance and coaching worth provide valuable information about the quality of the working relationship between teachers and coaches, these measures rely on *de facto* ratings from the perspective of teachers and/or coaches, and are therefore susceptible to temporal (recall) bias. In this regard, research examining quality of implementation might benefit from an interpersonal perspective, such that the relationship between teachers and coaches becomes the focal unit of analysis. Research on implementation might benefit from observed measures of specific dimensions of teacher-coach quality of interaction that can be linked to improvements in teachers’ practices in classrooms.

Quality of implementation might be considered a moderator of the effects of program on teacher outcomes, classroom quality of interactions, and child outcomes. As discussed above, coaching is pivotal in helping teachers to develop the social and emotional skills needed to provide emotional support in their classrooms, which in turn might contribute to improving children’s academic and SEL outcomes. Path analysis might be a useful alternative for researchers interested in examining multiple mediating mechanisms by which programs affect child outcomes. Such complex analyses may illuminate how programs generate positive changes in teachers social and emotional skills when implemented with high quality, and enable effective and sustainable high quality interactions in classrooms and improvements in child academic and social and emotional functioning.

The compliance propensity approach could be extended to include factors pertaining to program implementation at the school and district level that might influence teachers’ propensity for high quality program implementation. The support schools receive from districts to allocate resources needed to implement the program, the school climate and school level of preparedness to embark on structural changes, are factors that have been examined and shown to contribute to SEL program implementation and child outcomes (Kendziora and Osher, 2016; Oberle et al., 2016; Domitrovich et al., 2019). Using a multilevel propensity approach (Li et al., 2013; Leite et al., 2015; Li and Fraser, 2015; Fuentes et al., 2021) could contribute to the understanding of the effects of teacher propensity for high quality program implementation on classroom and child outcomes, while accounting for their transactions with and within broader levels of the context. This dynamic system perspective of program implementation is consistent with the idea of a holistic comprehension of quality of implementation proposed in this study.

## Data availability statement

The original contributions presented in the study are included in the article/Supplementary material, further inquiries can be directed to the initial study Co-PIs, JB (cjobrown@fordham.edu) and JD (jd2fe@virginia.edu).

## Ethics statement

The studies involving human participants were reviewed and approved by the Institutional Review Board (IRB) at Fordham University. Written informed consent to participate in this study was provided by the participants’ legal guardian/next of kin.

## Author contributions

All authors listed have made a substantial, direct, and intellectual contribution to the work and approved it for publication.

## Conflict of interest

The authors declare that the research was conducted in the absence of any commercial or financial relationships that could be construed as a potential conflict of interest.

## Publisher’s note

All claims expressed in this article are solely those of the authors and do not necessarily represent those of their affiliated organizations, or those of the publisher, the editors and the reviewers. Any product that may be evaluated in this article, or claim that may be made by its manufacturer, is not guaranteed or endorsed by the publisher.

## References

[ref1] Aber L. Brown J. L. Jones S. M. Berg J. Torrente C. (2011). School-based strategies to prevent violence, trauma, and psychopathology: the challenges of going to scale. Dev. Psychopathol. 23, 411–421. doi: 10.1017/S0954579411000149, PMID: 23786686

[ref2] Aber J. L. Jones S. M. Brown J. L. Chaudry N. Samples F. (1998). Resolving conflict creatively: evaluating the developmental effects of a school-based violence prevention program in neighborhood and classroom context. Dev. Psychopathol. 10, 187–213. doi: 10.1017/S0954579498001576 9635221

[ref3] Abry T. Rimm-Kaufman S. E. Larsen R. A. Brewer A. J. (2013). The influence of fidelity of implementation on teacher–student interaction quality in the context of a randomized controlled trial of the responsive classroom approach. J. Sch. Psychol. 51, 437–453. doi: 10.1016/j.jsp.2013.03.001, PMID: 23870440

[ref150] Ashworth E. Demkowicz O. Lendrum A. Frearson K. (2018). Coaching Models of School-Based Prevention and Promotion Programmes: A Qualitative Exploration of UK Teachers’ Perceptions. School Mental Health 10, 287–300. 30147801 10.1007/s12310-018-9282-3 PMC6096953

[ref4] Baird J. R. (1986). Improving learning through enhanced metacognition: a classroom study. Eur. J. Sci. Educ. 8, 263–282. doi: 10.1080/0140528860080303

[ref5] Baker M. L. Sigmon J. N. Nugent E. M. (2001). Truancy reduction: Keeping students in school. Washington, DC: U.S. Department of Justice, Office of Justice Programs, Office of Juvenile Justice and Delinquency Prevention.

[ref6] Barth R. P. (1984). Reducing nonattendance in elementary schools. Soc. Work. Educ. 6, 151–166. doi: 10.1093/cs/6.3.151

[ref7] Beaujean A. A. Beaujean M. A. A. (2012). Package ‘BaylorEdPsych’. R Package Version 0.5. Waco, TX: Baylor University.[Google Scholar].

[ref8] Berg J. K. Bradshaw C. P. Jo B. Ialongo N. S. (2017). Using complier average causal effect estimation to determine the impacts of the good behavior game preventive intervention on teacher implementers. Adm. Policy Ment. Health Ment. Health Serv. Res. 44, 558–571. doi: 10.1007/s10488-016-0738-1, PMID: 27207372

[ref9] Blair C. (2002). School readiness: integrating cognition and emotion in a neurobiological conceptualization of children's functioning at school entry. Am. Psychol. 57, 111–127. doi: 10.1037/0003-066X.57.2.111 11899554

[ref10] Booren L. M. Downer J. T. Vitiello V. E. (2012). Observations of children's interactions with teachers, peers, and tasks across preschool classroom activity settings. Early Educ. Dev. 23, 517–538. doi: 10.1080/10409289.2010.548767, PMID: 25717282 PMC4337404

[ref11] Brackett M. A. Rivers S. E. Reyes M. R. Salovey P. (2012). Enhancing academic performance and social and emotional competence with the RULER feeling words curriculum. Learn. Individ. Differ. 22, 218–224. doi: 10.1016/j.lindif.2010.10.002

[ref12] Bradshaw C. P. Pas E. T. Domitrovich C. E. Reinke W. M. Herman K. Poduska J. M. (2009). Measure of coach and teacher Alliance-coach report. Baltimore: Johns Hopkins University.

[ref160] Bronfenbrenner U. Morris P. A. (2010). The ecology of developmental processes, In Handbook of child psychology: Vol. 1. Theoretical models of human development eds. W. Damon and R. M. Lerner, 5th Ed. (New York: Wiley), 993–1029.

[ref13] Brown J. L. Jones S. M. LaRusso M. D. Aber J. L. (2010). Improving classroom quality: teacher influences and experimental impacts of the 4Rs program. J. Educ. Psychol. 102, 153–167. doi: 10.1037/a001816

[ref14] Brown J. L. Lowenstein A. E. Sutton E. Carlton R. Downer J. T. (2019). Experimental impacts of the 4Rs+MTP program on teachers’ Well-being, classroom interactions, and Children’s social-emotional and academic development. In: *Paper presented at the Society for Research on educational effectiveness*, Washington, DC.

[ref15] Collaborative for Academic, Social, and Emotional Learning. (2017). What is SEL? Collaborative for academic, social, and emotional learning. Available at: http://www.casel.org/what-is-sel/

[ref16] Conduct Problems Prevention Research Group. (1990). Social competence scale—teacher version. Duke University, Fast Track, Durham, NC. Available at: http://www.fasttrackproject.org/techrept/s/sct/sct.pdf (Accessed 22 December 2009).

[ref17] Curby T. W. Rimm-Kaufman S. E. Ponitz C. C. (2009). Teacher–child interactions and children’s achievement trajectories across kindergarten and first grade. J. Educ. Psychol. 101, 912–925. doi: 10.1037/a0016647

[ref18] Dahlberg L. L. Toal S. B. Behrens C. B. (1998). Measuring violence-related attitudes, beliefs, and behaviors among youths: A compendium of assessment tools. Atlanta, GA.: Division of Violence Prevention. National Center for Injury Prevention and Control, Centers for Disease Control and Prevention.

[ref19] Dane A. V. Schneider B. H. (1998). Program integrity in primary and early secondary prevention: are implementation effects out of control? Clin. Psychol. Rev. 18, 23–45. doi: 10.1016/S0272-7358(97)00043-3, PMID: 9455622

[ref20] Derzon J. H. Sale E. Springer J. F. Brounstein P. (2005). Estimating intervention effectiveness: synthetic projection of field evaluation results. J. Prim. Prev. 26, 321–343. doi: 10.1007/s10935-005-5391-5, PMID: 15995802

[ref21] Dix K. Keeves J. P. Slee P. T. Lawson M. J. Russell A. Askell-Williams H.. (2010). KidsMatter primary evaluation: technical report and user guide. Available at: https://research.acer.edu.au/cgi/viewcontent.cgi?article=1024&context=learning_processes (Accessed 26 October 2019)

[ref22] Dix K. L. Slee P. T. Lawson M. J. Keeves J. P. (2012). Implementation quality of whole-school mental health promotion and students’ academic performance. Child Adolesc. Mental Health 17, 45–51. doi: 10.1111/j.1475-3588.2011.00608.x, PMID: 22518095 PMC3320658

[ref23] Dodge K. A. Pettit G. S. McClaskey C. L. Brown J. (1986). Social competence in children. Monogr. Soc. Res. Child Dev. 51:i. doi: 10.2307/11659063807927

[ref24] Domitrovich C. E. Bradshaw C. P. Poduska J. M. Hoagwood K. Buckley J. A. Olin S.. (2008). Maximizing the implementation quality of evidence-based preventive interventions in schools: a conceptual framework. Adv. School Ment. Health Promot. 1, 6–28. doi: 10.1080/1754730X.2008.9715730, PMID: 27182282 PMC4865398

[ref25] Domitrovich C. E. Gest S. D. Jones D. Gill S. DeRousie R. M. S. (2010). Implementation quality: lessons learned in the context of the head start REDI trial. Early Child Res. Q. 25, 284–298. doi: 10.1016/j.ecresq.2010.04.001, PMID: 22844183 PMC3404616

[ref26] Domitrovich C. E. Li Y. Mathis E. T. Greenberg M. T. (2019). Individual and organizational factors associated with teacher self-reported implementation of the PATHS curriculum. J. Sch. Psychol. 76, 168–185. doi: 10.1016/j.jsp.2019.07.015, PMID: 31759464

[ref27] Downer J. T. Kraft-Sayre M. E. Pianta R. C. (2009a). Ongoing, web mediated professional development focused on teacher–child interactions: Early childhood educators’ usage rates and self-reported satisfaction. Early Educ. Dev. 20, 321–345. doi: 10.1080/10409280802595425, PMID: 34483629 PMC8412435

[ref28] Downer J. T. LoCasale-Crouch J. Hamre B. K. Pianta R. C. (2009b). Teacher characteristics associated with responsiveness and exposure to consultation and online professional development resources. Early Educ. Dev. 20, 431–455. doi: 10.1080/10409280802688626, PMID: 25419081 PMC4240009

[ref29] Doyle N. B. Downer J. T. Brown J. L. Lowenstein A. E. (2022). Understanding high quality teacher-student interactions in high needs elementary schools: an exploration of teacher, student, and relational contributors. Sch. Ment. Heal. 14, 997–1010. doi: 10.1007/s12310-022-09519-0

[ref30] Doyle N. B. Gomez Varon J. Downer J. T. Brown J. L. (under review). Testing the integration of a teacher coaching model and a social-emotional learning and literacy intervention in urban elementary schools.

[ref31] Durlak J. A. (2016). Programme implementation in social and emotional learning: basic issues and research findings. Camb. J. Educ. 46, 333–345. doi: 10.1080/0305764X.2016.1142504

[ref32] Durlak J. A. DuPre E. P. (2008). Implementation matters: a review of research on the influence of implementation on program outcomes and the factors affecting implementation. Am. J. Community Psychol. 41, 327–350. doi: 10.1007/s10464-008-9165-0, PMID: 18322790

[ref33] Durlak J. Weissberg R. Dymnicki A. Schellinger K. (2011). The impact of enhancing students’ social and emotional learning: a meta-analysis of school-based universal interventions. Child Dev. 82, 405–432. doi: 10.1111/j.1467-8624.2010.01564.x, PMID: 21291449

[ref34] Dusenbury L. Brannigan R. Hansen W. B. Walsh J. Falco M. (2004). Quality of implementation: developing measures crucial to understanding the diffusion of preventive interventions. Health Educ. Res. 20, 308–313. doi: 10.1093/her/cyg134, PMID: 15522898

[ref35] Early D. M. Maxwell K. L. Ponder B. D. Pan Y. (2017). Improving teacher-child interactions: a randomized controlled trial of making the Most of classroom interactions and my teaching partner professional development models. Early Child Res. Q. 38, 57–70. doi: 10.1016/j.ecresq.2016.08.005

[ref36] Edwards J. R. Lambert L. S. (2007). Methods for integrating moderation and mediation: a general analytical framework using moderated path analysis. Psychol. Methods 12, 1–22. doi: 10.1037/1082-989X.12.1.1, PMID: 17402809

[ref37] Finch W. H. (2022). Multivariate analysis of variance for multilevel data: a simulation study comparing methods. J. Exp. Educ. 90, 173–190. doi: 10.1080/00220973.2020.1718058

[ref38] Fixsen D. L. Blase K. A. Naoom S. F. Wallace F. (2009). Core implementation components. Res. Soc. Work. Pract. 19, 531–540. doi: 10.1177/1049731509335549

[ref39] Follmann D. A. (2000). On the effect of treatment among would-be treatment compliers: an analysis of the multiple risk factor intervention trial. J. Am. Stat. Assoc. 95, 1101–1109. doi: 10.1080/01621459.2000.10474306

[ref40] Fuentes A. Lüdtke O. Robitzsch A. (2021). Causal inference with multilevel data: a comparison of different propensity score weighting approaches. Multivar. Behav. Res., 1–24. 10.1080/00273171.2021.1925521 34128730

[ref41] Grant S. Hamilton L. S. Wrabel S. L. Gomez C. J. Whitaker A. Leschitz J. T.., (2017). Social and emotional learning interventions under the every student succeeds act: Evidence review. RAND Corporation. Santa Monica, CA.

[ref42] Hadden D. S. Pianta R. B. (2006). “Clinical consultation with teachers for improved preschool literacy instruction” in Clinical approaches to emergent literacy intervention, ed. Justice L. M. (San Diego, CA: Plural Publishing) 99–124.

[ref43] Hamre B. K. Pianta R. C. (2005). Can instructional and emotional support in the first grade classroom make a difference for children at risk of school failure? Child Dev. 76, 949–967. doi: 10.1111/j.1467-8624.2005.00889.x, PMID: 16149994

[ref44] Hamre B. K. Pianta R. C. (2007). “Learning opportunities in preschool and early elementary classrooms” in School readiness and the transition to kindergarten in the era of accountability. eds. Pianta R. C. Cox M. J. Snow K. L. (Baltimore, MD, US: Paul H Brookes Publishing), 49–83.

[ref45] Hamre B. K. Pianta R. C. Downer J. T. DeCoster J. Mashburn A. J. Jones S. M.. (2013). Teaching through interactions: testing a developmental framework of teacher effectiveness in over 4,000 classrooms. Elem. Sch. J. 113, 461–487. doi: 10.1086/669616, PMID: 34497425 PMC8423353

[ref47] Hothorn T. Hornik K. Zeileis A. (2006). Unbiased recursive partitioning: a conditional inference framework. J. Comput. Graph. Stat. 15, 651–674. doi: 10.1198/106186006X133933

[ref48] Humphrey N. Barlow A. Lendrum A. (2018). Quality matters: implementation moderates student outcomes in the PATHS curriculum. Prev. Sci. 19, 197–208. doi: 10.1007/s11121-017-0802-4, PMID: 28631234 PMC5801378

[ref49] Humphrey N. Barlow A. Wigelsworth M. Lendrum A. Pert K. Joyce C.. (2016). A cluster randomized controlled trial of the promoting alternative thinking strategies (PATHS) curriculum. J. Sch. Psychol. 58, 73–89. doi: 10.1016/j.jsp.2016.07.002, PMID: 27586071 PMC5019026

[ref50] Imbens G. W. Rubin D. B. (1997). Bayesian inference for causal effects in randomized experiments with noncompliance. Ann. Stat. 25, 305–327. doi: 10.1214/aos/1034276631

[ref51] Jamshidian M. Jalal S. Jansen C. (2014). MissMech: an R package for testing homoscedasticity, multivariate normality, and missing completely at random (MCAR). J. Stat. Softw. 56, 1–31. doi: 10.18637/jss.v056.i06

[ref52] Jones S. Bailey R. Kahn J. (2019). The science and practice of social and emotional learning: Implications for state policymaking State Education Standard, 19, 18–24.

[ref53] Jones S. M. Brown J. L. Aber J. (2011). Two-year impacts of a universal school-based social-emotional and literacy intervention: an experiment in translational developmental research. Child Dev. 82, 533–554. doi: 10.1111/j.1467-8624.2010.01560.x 21410922

[ref54] Jones S. M. Brown J. L. Hoglund W. L. Aber J. L. (2010). A school-randomized clinical trial of an integrated social–emotional learning and literacy intervention: impacts after 1 school year. J. Consult. Clin. Psychol. 78, 829–842. doi: 10.1037/a0021383, PMID: 21114343

[ref55] Jorgensen T. D. Pornprasertmanit S. Schoemann A. M. Rosseel Y. Miller P. Quick C.. (2019). semTools: Useful tools for structural equation modeling. R package version 0.5-2. Retrieved from https://CRAN.R-project.org/package=semTools.

[ref56] Kamphaus R. W. Reynolds C. R. (1998). BASC monitor for ADHD. Circle Pines, MN: AGS Publishing.

[ref57] Kendziora K. Osher D. (2016). Promoting children’s and adolescents’ social and emotional development: district adaptations of a theory of action. J. Clin. Child Adolesc. Psychol. 45, 797–811. doi: 10.1080/15374416.2016.1197834 27611060

[ref58] Kingston B. Wilensky R. (2018). Building adult social and emotional capacity: a key ingredient for unleashing the power of prevention. J. Soc. Soc. Work Res. 9, 783–797. doi: 10.1086/700655

[ref59] Kleinke K. (2017). Multiple imputation under violated distributional assumptions: a systematic evaluation of the assumed robustness of predictive mean matching. J. Educ. Behav. Stat. 42, 371–404. doi: 10.3102/1076998616687084

[ref60] Koo T. K. Li M. Y. (2016). A guideline of selecting and reporting intraclass correlation coefficients for reliability research. J. Chiropr. Med. 15, 155–163. doi: 10.1016/j.jcm.2016.02.012 27330520 PMC4913118

[ref61] Kraft M. A. Blazar D. Hogan D. (2018). The effect of teacher coaching on instruction and achievement: a meta-analysis of the causal evidence. Rev. Educ. Res. 88, 547–588. doi: 10.3102/0034654318759268

[ref62] Leite W. L. Jimenez F. Kaya Y. Stapleton L. M. MacInnes J. W. Sandbach R. (2015). An evaluation of weighting methods based on propensity scores to reduce selection bias in multilevel observational studies. Multivar. Behav. Res. 50, 265–284. doi: 10.1080/00273171.2014.991018, PMID: 26610029

[ref63] Lendrum A. Humphrey N. (2012). The importance of studying the implementation of interventions in school settings. Oxf. Rev. Educ. 38, 635–652. doi: 10.1080/03054985.2012.734800

[ref64] Lendrum A. Humphrey N. Greenberg M. (2016). “Implementing for success in school-based mental health promotion: the role of quality in resolving the tension between fidelity and adaptation” in Mental health and wellbeing through schools: The way forward. eds. Shute R. Slee P. (Hove: Routledge), 53–63.

[ref65] Li J. Fraser M. W. (2015). Evaluating dosage effects in a social-emotional skills training program for children: an application of generalized propensity scores. J. Soc. Serv. Res. 41, 345–364. doi: 10.1080/01488376.2014.994797

[ref66] Li F. Zaslavsky A. M. Landrum M. B. (2013). Propensity score weighting with multilevel data. Stat. Med. 32, 3373–3387. doi: 10.1002/sim.5786, PMID: 23526267 PMC3710526

[ref67] Little R. J. A. (1988). A test of missing completely at random for multivariate data with missing values. J. Am. Stat. Assoc. 83, 1198–1202. doi: 10.1080/01621459.1988.10478722

[ref68] Louppe G. Wehenkel L. Sutera A. Geurts P. (2013). “Understanding variable importances in forests of randomized trees” in Advances in neural information processing systems eds. C. J. Burges and L. Bottou and M. Welling and Z. Ghahramani and K.Q. Weinberger (Red Hood, NY: Curran Associates), 431–439.

[ref69] Lovibond P. F. Lovibond S. H. (1995). The structure of negative emotional states: comparison of the depression anxiety stress scales (DASS) with the Beck depression and anxiety inventories. Behav. Res. Ther. 33, 335–343. doi: 10.1016/0005-7967(94)00075-U, PMID: 7726811

[ref70] MacKinnon D. P. Lockwood C. M. Williams J. (2004). Confidence limits for the indirect effect: distribution of the product and resampling methods. Multivar. Behav. Res. 39, 99–128. doi: 10.1207/s15327906mbr3901_4, PMID: 20157642 PMC2821115

[ref71] Maslach C. Jackson S. E. Leiter M. P. Schaufeli W. B. Schwab R. L. (1986). Maslach burnout inventory (Vol. 21, pp. 3463–3464). Palo Alto, CA: Consulting Psychologists Press.

[ref72] Maslach C. Schaufeli W. B. Leiter M. P. (2001). Job burnout. Annu. Rev. Psychol. 52, 397–422. doi: 10.1146/annurev.psych.52.1.397 11148311

[ref73] Masten A. S. Cicchetti D. (2010). Developmental cascades. Dev. Psychopathol. 22, 491–495. doi: 10.1017/S0954579410000222 20576173

[ref74] McCluskey C. P. Bynum T. S. Patchin J. W. (2004). Reducing chronic absenteeism: an assessment of an early truancy initiative. Crime Delinq. 50, 214–234. doi: 10.1177/0011128703258942

[ref75] McCoach D. B. Adelson J. L. (2010). Dealing with dependence (part I): understanding the effects of clustered data. Gift. Child Q. 54, 152–155. doi: 10.1177/0016986210363076

[ref76] Meyers D. C. Durlak J. A. Wandersman A. (2012). The quality implementation framework: a synthesis of critical steps in the implementation process. Am. J. Community Psychol. 50, 462–480. doi: 10.1007/s10464-012-9522-x, PMID: 22644083

[ref77] Musci R. J. Pas E. T. Bettencourt A. F. Masyn K. E. Ialongo N. S. Bradshaw C. P. (2019). How do collective student behavior and other classroom contextual factors relate to teachers’ implementation of an evidence-based intervention? A multilevel structural equation model. Dev. Psychopathol. 31, 1827–1835. doi: 10.1017/S095457941900097X 31439069

[ref78] Muthén B. (2004). “Latent variable analysis: Growth mixture modeling and related techniques for longitudinal data” in Handbook of quantitative methodology for the social sciences, ed. D. Kaplan (Newbury Park, CA, US: Sage Publications), 345–368.

[ref79] Neace W. P. Munoz M. A. (2012). Pushing the boundaries of education: evaluating the impact of second step®: a violence prevention curriculum with psychosocial and non-cognitive measures. Child Youth Serv. 33, 46–69. doi: 10.1080/0145935X.2012.665324

[ref80] Oberle E. Domitrovich C. E. Meyers D. C. Weissberg R. P. (2016). Establishing systemic social and emotional learning approaches in schools: a framework for schoolwide implementation. Camb. J. Educ. 46, 277–297. doi: 10.1080/0305764X.2015.1125450

[ref81] Oberski D. (2016). “Mixture models: latent profile and latent class analysis” in Modern statistical methods for HCI Human–Computer Interaction Series, eds. J. Robertson, M. Kaptein, (Cham: Springer), 275–287. doi: 10.1007/978-3-319-26633-6_12

[ref82] Orpinas P. Frankowski R. (2001). The aggression scale: a self-report measure of aggressive behavior for young adolescents. J. Early Adolesc. 21, 50–67. doi: 10.1177/0272431601021001003

[ref83] Panayiotou M. Humphrey N. Hennessey A. (2019). Implementation matters: using complier average causal effect estimation to determine the impact of the promoting alternative thinking strategies (PATHS) curriculum on children’s quality of life. J. Educ. Psychol. 112, 236–253. doi: 10.1037/edu0000360

[ref84] Pas E. T. Bradshaw C. P. Becker K. D. Domitrovich C. Berg J. Musci R.. (2015). Identifying patterns of coaching to support the implementation of the good behavior game: the role of teacher characteristics. Sch. Ment. Heal. 7, 61–73. doi: 10.1007/s12310-015-9145-0

[ref85] Patti J. Holzer A. A. Brackett M. A. Stern R. (2015). Twenty-first-century professional development for educators: a coaching approach grounded in emotional intelligence. Coaching 8, 96–119. doi: 10.1080/17521882.2015.1061031

[ref86] Pianta R. C. Hamre B. K. (2009). Conceptualization, measurement, and improvement of classroom processes: standardized observation can leverage capacity. Educ. Res. 38, 109–119. doi: 10.3102/0013189X09332374

[ref87] Pianta R. C. Hamre B. K. Mintz S. L. (2012). Classroom assessment scoring system (CLASS) upper elementary manual, Charlottesville, VA: Teachstone.

[ref88] Pianta R. C. Mashburn A. J. Downer J. T. Hamre B. K. Justice L. (2008). Effects of web-mediated professional development resources on teacher–child interactions in pre-kindergarten classrooms. Early Child Res. Q. 23, 431–451. doi: 10.1016/j.ecresq.2008.02.001, PMID: 25717217 PMC4337032

[ref89] Portnow S. Downer J. T. Brown J. (2018). Reductions in aggressive behavior within the context of a universal, social emotional learning program: classroom-and student-level mechanisms. J. Sch. Psychol. 68, 38–52. doi: 10.1016/j.jsp.2017.12.004, PMID: 29861030

[ref90] Preacher K. J. Selig J. P. (2012). Advantages of Monte Carlo confidence intervals for indirect effects. Commun. Methods Meas. 6, 77–98. doi: 10.1080/19312458.2012.679848

[ref91] Reid K. (2013). Managing school attendance: Successful intervention strategies for reducing truancy, London: Routledge.

[ref92] Reyes M. R. Brackett M. A. Rivers S. E. White M. Salovey P. (2012). Classroom emotional climate, student engagement, and academic achievement. J. Educ. Psychol. 104, 700–712. doi: 10.1037/a0027268

[ref93] Rimm-Kaufman S. E. Fan X. Chiu Y. J. You W. (2007). The contribution of the responsive classroom approach on children's academic achievement: results from a three year longitudinal study. J. Sch. Psychol. 45, 401–421. doi: 10.1016/j.jsp.2006.10.003

[ref94] Rockwood N. J. (2017). Advancing the formulation and testing of multilevel mediation and moderated mediation models. Doctoral dissertation, The Ohio State University.

[ref95] Roeser R. W. Eccles J. S. Sameroff A. J. (2000). School as a context of early adolescents' academic and social-emotional development: a summary of research findings. Elem. Sch. J. 100, 443–471. doi: 10.1086/499650

[ref96] Rosenblatt J. L. Elias M. J. (2008). Dosage effects of a preventive social-emotional learning intervention on achievement loss associated with middle school transition. J. Prim. Prev. 29, 535–555. doi: 10.1007/s10935-008-0153-9, PMID: 19015991

[ref97] Rubin D. B. (1987). Multiple imputation for nonresponse in surveys. New York (NY): J. Wiley & Sons.

[ref98] Rucinski C. L. Brown J. L. Downer J. T. (2018). Teacher–child relationships, classroom climate, and children’s social-emotional and academic development. J. Educ. Psychol. 110, 992–1004. doi: 10.1037/edu0000240

[ref99] Ryff C. D. Keyes C. L. M. (1995). The structure of psychological well-being revisited. J. Pers. Soc. Psychol. 69, 719–727. doi: 10.1037/0022-3514.69.4.719 7473027

[ref100] Sagarin B. J. West S. G. Ratnikov A. Homan W. K. Ritchie T. D. Hansen E. J. (2014). Treatment noncompliance in randomized experiments: statistical approaches and design issues. Psychol. Methods 19, 317–333. doi: 10.1037/met0000013, PMID: 24773358

[ref180] Schaufeli W. B. Bakker A. B. Hoogduin K. Schaap C. Kladler A. (2001). On the clinical validity of the Maslach Burnout Inventory and the Burnout Measure. Psychology & health 16, 565–582. 22804499 10.1080/08870440108405527

[ref101] Shah A. D. Bartlett J. W. Carpenter J. Nicholas O. Hemingway H. (2014). Comparison of random forest and parametric imputation models for imputing missing data using MICE: a CALIBER study. Am. J. Epidemiol. 179, 764–774. doi: 10.1093/aje/kwt312, PMID: 24589914 PMC3939843

[ref102] Shrout P. E. Fleiss J. L. (1979). Intraclass correlation; uses in assessing rater reliability. Psychol. Bull. 86, 420–428. doi: 10.1037/0033-2909.86.2.420 18839484

[ref103] Slee P. T. Lawson M. J. Russell A. Askell-Williams H. Dix K. L. Owens L.. (2009). KidsMatter primary evaluation final report. Melbourne: beyond blue. Available at: https://dspace2.flinders.edu.au/xmlui/bitstream/handle/2328/26832/Slee20KidsMatter20Evaluation20final20report.pdf?sequence=1&isAllowed=y

[ref104] Smith A. Hutchinson K. (2022). “Planning for implementation: why, who, and how” in Implementation Science, eds. F. Rapport, R. Clay-Williams, and J. Braithwait (London, England: Routledge), 130–135.

[ref105] Strobl C. Boulesteix A. L. Kneib T. Augustin T. Zeileis A. (2008). Conditional variable importance for random forests. BMC Bioinformatics 9:307. doi: 10.1186/1471-2105-9-307 18620558 PMC2491635

[ref106] Taylor R. D. Oberle E. Durlak J. A. Weissberg R. P. (2017). Promoting positive youth development through school-based social and emotional learning interventions: a meta-analysis of follow-up effects. Child Dev. 88, 1156–1171. doi: 10.1111/cdev.12864, PMID: 28685826

[ref107] Teasley M. L. (2004). Absenteeism and truancy: risk, protection, and best practice implications for school social workers. Child. Sch. 26, 117–128. doi: 10.1093/cs/26.2.117

[ref108] Tingley D. Yamamoto T. Hirose K. Keele L. Imai K. (2014). Mediation: R package for causal mediation analysis. Journal of Statistical Software 59, 1–38. doi: 10.1111/cdev.12864

[ref109] Tolan P. Elreda L. M. Bradshaw C. P. Downer J. T. Ialongo N. (2020). Randomized trial testing the integration of the good behavior game and MyTeachingPartner™: the moderating role of distress among new teachers on student outcomes. J. Sch. Psychol. 78, 75–95. doi: 10.1016/j.jsp.2019.12.002, PMID: 32178813

[ref110] Watson D. Clark L. A. Tellegen A. (1988). Development and validation of brief measures of positive and negative affect: the PANAS. J. Pers. Soc. Psychol. 54, 1063–1070. doi: 10.1037/0022-3514.54.6.1063, PMID: 3397865

[ref111] Wehby J. H. Maggin D. M. Partin T. C. M. Robertson R. (2012). The impact of working alliance, social validity, and teacher burnout on implementation fidelity of the good behavior game. Sch. Ment. Heal. 4, 22–33. doi: 10.1007/s12310-011-9067-4

[ref190] Weiss M.J. Bloom H. S. Brock T. (2014). A conceptual framework for studying the sources of variation in program effects Journal of Policy Analysis and Management 33, 778–808.

[ref112] Weiss S. Muckenthaler M. Heimlich U. Kuechler A. Kiel E. (2021). Teaching in inclusive schools. Do the demands of inclusive schools cause stress? Int. J. Incl. Educ. 25, 588–604. doi: 10.1080/13603116.2018.1563834

[ref113] Weissberg R. P. (2019). Promoting the social and emotional learning of millions of school children. Perspect. Psychol. Sci. 14, 65–69. doi: 10.1177/1745691618817756, PMID: 30799753

[ref114] Welch B. L. (1947). The generalization of Student’s problem when several different population variances are involved. Biometrika 34, 28–35. doi: 10.2307/2332510, PMID: 20287819

[ref115] Zhang Z. (2016). Multiple imputation with multivariate imputation by chained equation (MICE) package. Annal Transl Med 4:30. doi: 10.3978/j.issn.2305-5839.2015.12.63, PMID: 26889483 PMC4731595

[ref116] Zhao P. Su X. Ge T. Fan J. (2016). Propensity score and proximity matching using random forest. Contemp. Clin. Trials 47, 85–92. doi: 10.1016/j.cct.2015.12.012, PMID: 26706666 PMC4818178

