# Supplementary material for: High quality implementation of 4Rs + MTP increases classroom emotional support and reduces absenteeism
Source: Front Psychol. 2023 Apr 27;14:1065749. doi: 10.3389/fpsyg.2023.1065749 (PMC10172679; doi:10.3389/fpsyg.2023.1065749)
Supplement: Supplementary file 1 [file Data_Sheet_1.DOCX]

Multilevel moderated mediation. This study used multilevel modeling to test the relations between a level-2 predictor (i.e., random assignment) and a level-1 outcome (i.e., child outcome), mediated by a level 2 variable. This multilevel mediation is examined at different levels of the level-2 moderator (i.e., teacher compliance propensity). A series of regressions were used to estimate the moderation of teacher compliance propensity on the relationship between random assignment and child outcomes mediated by the quality of classroom interaction. This method results from and adaptation of the general path analytic framework proposed by Edward & Lambert (2007) for testing the direct, indirect, and total effects at different levels of the moderator. This framework first depicts the mediation as a path model, expressing the relationships among variables with regression equations, and then, introduces the moderator variable and its product with the independent variable and the mediator variable into the equations. Although Edwards and Lambert (2007) do not discuss the moderated mediation in a multilevel path, to account for the nested structure of students within teachers/classrooms in this study, a random intercept is also included in the regression equations (Finch, Bolin, & Kelley, 2016; Tingley et al., 2014; Rockwood, 2017).

The moderator, teacher compliance propensity, differs in how it affects different paths of the mediation model. Teacher compliance propensity, defined as the propensity to implement program activities in classrooms and receive support for the implementation*,* is thought to affect both classroom interactions and the behaviors of children in these classrooms. Therefore, the model includes the moderation of Teacher Compliance Propensity (*Compliance*, from now on) in the effects of random assignment to program (*Program*, from now on) on *Classroom Interaction*, and *Child Outcomes* (see Figure 1 below).

The regression equations of the multilevel moderated mediation model are integrated into a reduced form equation, resulted from substituting the regression equation for the mediator variable *Classroom Interaction* (Equation 1) into the equation for the dependent variable *Child Outcome* (Equation 2).

$Classroom Interaction=y_{00}+$ $Classroom Interaction=y_{m0}+$ $y_{m1}Program+y_{m2}Compliance+y_{m12}Program*Compliance$ $y_{m1}DosageT+y_{m2}Adherence+y_{m12}DosageT*Adherence+y_{m3}Coaching+y_{m13}DosageT*Coaching+e_{m}$ $+e_{m}$ (1)

Figure 1.

Moderation of Compliance on the relations among Program, Classroom Interactions and Child Outcome.

Equation (1) represents the regression of the mediator *Classroom Interaction* on *Program.* This effect is moderated by the propensity of compliance represented with the inclusion of *Compliance* and its interaction term: *Program*Compliance.* The subscript “*m”* instead of the conventional “0” is preferred to later distinguish between coefficients associated with the indirect effects through the mediator from coefficients associated with the direct effects of the predictor and moderators, once this equation is integrated into equation (2):

$Child Outcome=y_{00}+u_{0j}+$ $y_{02}Program+y_{03}Compliance+y_{023}Program*Compliance$ $+$ $y_{04}Classroom Interaction$ $+e_{ij}$ (2)

Equation (2) shows the regression of the dependent variable *Child Outcome* on the main predictor *Program*. This equation also includes the mediator *Classroom Interaction* and the moderator *Compliance* with its interaction term:  *Program* Compliance.* To reflect the nested structure of the outcome, a random term $u_{0j}$ is added to the intercept. In the following, the mediator *Classroom Interaction* in equation (2) is replaced by the term from equation (1), and the equation is rearranged to clearly depict the terms for the moderation of the direct and indirect effects (Edwards and Lambert, 2007)

$=y_{00}+u_{0j}+$ $y_{02}Program+y_{03}Compliance+y_{023}Program*Compliance+$

$y_{04} (y_{m0}+ y_{m1}Program+y_{m2}Compliance+y_{m12}Program*Compliance+e_{m}$*)* $+e_{ij}$

$=y_{00}+u_{0j}+ y_{02}Program +y_{03}Compliance+y_{023}w_{ij}Program*Compliance+ y_{m0}y_{04}+$ $y_{m1}y_{04}Program+y_{m2}y_{04}Compliance+y_{m12}y_{04}Program* Compliance$ $+{y_{04}e}_{m}$ $+e_{ij}$

$=y_{00}+u_{0j}+\text{ }y_{m0}y_{04}+$ ${(y}_{02}+\text{ }y_{m1}y_{04})Program+{(y}_{03}+y_{m2}y_{04})Compliance+$

${(y}_{023}+y_{m12}y_{04})Program*Compliance+{y_{04}e}_{m}$ $+e_{ij}$

$=y_{00}+u_{0j}+\text{ }y_{m0}y_{04}+{(y}_{03}+y_{m2}y_{04})Compliance+$

$[{(y}_{02}+\text{ }y_{m1}y_{04}) +{(y}_{023}+y_{m12}y_{04})Compliance]Program+{y_{04}e}_{m}$ $+e_{ij}$

$={[y}_{00}+u_{0j}+y_{03}Compliance+(y_{m0}+y_{m2}Compliance)y_{04}]+$

$\left[ {(y}_{02}+y_{023}Compliance \right)+(y_{m1}+y_{m12}Compliance)y_{04}]Program$

$+e_{ij}+{y_{04}e}_{m}$ (2a)

The rearranged equation (2a) indicates that the first stage of the indirect effect of *Program* on *Child Outcome* varies as a function of the moderator: *Compliance*, as captured by the term:

$\left( y_{m1}+y_{m12}Adherence+y_{m13}\mathrm{Coaching} \right)y_{04}$ $\left( Y_{m1}+y_{m12}\mathrm{Compliance} \right)y_{04}$ .

Additionally, equation 2a shows that the direct effect of the Program on *Child Outcome* depends on the moderator, due to the term:

${(y}_{02}+y_{023}Compliance)$

The intercept of *Child outcome* is affected directly by *Compliance*, as reflected by the term:

$y_{03}Compliance$,

and indirectly by the effects of the moderator on the mediator *Classroom Interaction*, as reflected by the term:

$\left( y_{m0}+y_{m2}Compliance \right)y_{04}$

The random intercept accounting for the variability of *Child Outcome* across classroom is represented by the expression:

($y_{00}+u_{0j}$).

Finally, the expression

($e_{\mathrm{ij}}+{y_{04}e}_{m}$ ),

represents the error terms associated with the direct and indirect effect of *Program*, respectively.

The moderated path analysis framework implemented in this study uses equations containing products of regression coefficients, for instance the product term depicting the effects of the moderator on the mediator $\left( y_{m0}+y_{m2}Compliance \right)y_{04}$ in equation 2a. Since the sampling distribution of the product of random variables is nonnormal, even if the variables are normally distributed (Anderson, 1984), the effect of this product tested with regular procedures is biased (Edwards & Lambert, 2007; Zhang, 2014). In this case, it is recommended to test the effect of the product of the two regression coefficients using replication methods such as Monte Carlo replications with a minimum *N* = 1000. The Monte Carlo method generates a pseudo normal sampling distribution of the product of two regression coefficients by repeatedly estimating coefficients with the N number of replications (Zhang, 2014). Analysis of multilevel moderated mediation was conducted using the R package SemTools package (Jorgensen et al., 2019). Multilevel analysis was performed in multiple imputed datasets using structural equation models (SEM.mi) function. Monte Carlo test of mediation (MacKinnon, Lockwood, & Williams, 2004) was implemented to estimate intervals for the coefficients using 1000 replications, with a bias-corrected 90% confidence interval for results in the hypothesized direction. Simple effects were computed by substituting values of teacher compliance propensity into the equation 2a. Since teacher compliance propensity is a continuous moderator, and cut points are not clearly defined in the SEL implementation literature (Humphrey et al, 2018), representative scores from the distribution of teacher compliance propensity were used to depict above and below average compliance propensity (i.e., one standard deviation above and below the mean).
