# Supplementary material for: High quality implementation of 4Rs + MTP increases classroom emotional support and reduces absenteeism
Source: Front Psychol. 2023 Apr 27;14:1065749. doi: 10.3389/fpsyg.2023.1065749 (PMC10172679; doi:10.3389/fpsyg.2023.1065749)
Supplement: Supplementary file 2 [file Data_Sheet_2.DOCX]

**Distributional Properties and Missing Patterns in Implementation Data**

Implementation data was analyzed for patterns of missingness. In order to select the test to diagnose missing patterns (parametric vs non-parametric), a multivariate normality of implementation variables was examined using Shapiro-Wilk Multivariate Normality Test (Royston, 1982). Results showed that variables were non-normally distributed *W* = 0.669, *p* < .0001. It should be noted that the non-normal distribution of implementation variables does not affect analysis of latent profiles of teacher quality of implementation. Indeed, the non-normal distribution of a variable in latent profile analysis is assumed to be produced by mixing together two or more normal distributions of hidden (latent) groups that are not observed (Oberski, 2016).

Regarding the analysis of missing patterns in implementation variables, Jamshidian and Jalal (2010) recommend the Anderson-Darling *k*-sample test of homoscedasticity (Scholz & Stephens, 1987) for testing MCAR in non-normally distributed data. Results showed no identifiable missing patterns to be tested. Four teachers who attrited from the study before implementation measures were collected showed missing values in all variables. The other six teachers with missing values did not constitute a pattern since each of them had missing values in different variables. Therefore, there were no patterns to test whether missingness was completely at random (Jamshidian & Jalal, 2010). An additional test of equality of means to detect missing patterns was conducted on all implementation variables. Little’s test of equality of means (Little, 1988) yielded 7 missing patterns, with $x^{2}$ (47) = 127.642, p<0.001 although significant, it showed that one pattern corresponded to the four teachers who dropped the study before implementation and the other six patterns corresponded to missing values from six teachers, namely, one pattern for each teacher.

Inspection of outliers using Mahalanobis distance suggested six multivariate outliers in the implementation sample. Further inspection of these data points shows that the extreme values corresponded to the variable “Time spent visiting the website”, a variable representing the mean time spent by teachers in the 4Rs+MTP website. Close examination of the raw data for this variable showed anomalous patterns, such as unusual time logs and unidentified fields for the data recorded that prevented the distinction between someone who spent time visiting a given web resource from someone who just forgot to log out of the website. After deliberation with the website engineer, it was decided that this variable did not reliably measure the time teachers spent actually visiting the website, therefore it was excluded from further analyses of implementation data. Table 6 shows descriptive statistics of quality of implementation variables and percentage of missing observations per variable.

**Distributional Properties and Missing Data Patterns among Teacher-Classroom Variables and Student Outcomes**

Distribution of student level outcomes were non-normally distributed at time 1, W = 0.023, p < 0001, and time 2, W = 0.522, p < .0001. Similarly, teacher-classroom level variables were non-normally distributed at time 1, W = 0.114, p <.0001; and time 2, W = 0.138, p < .0001. Moderated mediation in the context of path analysis relies on the assumption of OLS regressions (Edwards & Lambert, 2007) and since normal distribution of the variables may influence test of significance, especially in the presence of highly skewed distribution, a cutoff of ± 2 values of skewness and ± 7 values of kurtosis was stablished for each variable.

MCAR tests were performed for both student and teacher-classroom variables at both time 1 and time 2. The MCAR test showed 12 missing data patterns for student-reported variables at time 1, (ELA and MATH scores were excluded since 3rd graders did not have data on these variables at time 1). At time 2, there were 19 missing data patterns for student-reported variables. A non-parametric test of homoscedasticity rejected the null hypothesis of MCAR at a .05 significance level, with a p <.0001 for student-reported variables at both time 1 and time 2. The test of MCAR for teacher-classroom variables showed three patterns of missing data for time 1, and two patterns at time 2. Results from the Anderson-Darling k-sample test of homoscedasticity suggested insufficient evidence to reject MCAR at 0.05 significance, with a p = 0.142 for time 1 and p = 0.771 for time 2.

Results of a test of equality of means to detect missing patterns in students’ variables showed 49 missing patterns at time 1 and, 48 at 2, with x^2 (463) = 3309.78, p < 0.001 and x^2 (461) = 1182.72, p < 0.001 for time 1 and time 2 respectively, suggesting that missing data were not MCAR at both time 1 and 2. For teacher-classroom variables, Little’s test of equality of means yields 34 missing patterns at both time 1 and time 2, with x^2 (609)= 712.92, p = 0.002 and x^2(570) = 2510.85, p<0.001 for time 1 and time 2, respectively. These results suggest missing data are not MCAR.

The dataset including the full sample of teachers and students was imputed using multiple imputation with multivariate imputation by chained equation (MICE) package (Zhang, 2016). Intraclass correlation coefficients were used as indicators of the amount of variance accounted by the clustering of students within teachers and schools, with high ICCs indicating high amount of shared variance. Failing to control for the common variance results in underestimation of standard errors, which in turn increases the chance of type I error. In education, a typical ICC for school effects is .22 (McCoac, & Adelson 2010). Intraclass correlations of student variables ranged between .06 and .37, and between .02 and .19 when clustered by teacher ID and by school ID, respectively (see Intraclass Correlations, ICC, Table 7). In order to account for the shared variance during the imputation process, random intercepts of student outcomes were included in the model and teacher ID was included as a cluster variable. Variables at level 2 (teacher-classroom) were imputed using the function 2only.pmm that aggregates level-1 predictors and imputes the level-2 variables using predictive mean matching (pmm; Kleinke, 2017). Variables at level 1 (student level) were imputed using random forest (Shah, Bartlett, Carpenter, Nicholas & Hemingway, 2014). Each imputation was performed separately for treatment and control groups and then combined into a single dataset. Twenty imputed datasets were used for analyses. Table 7 and 8 show descriptive information and proportion of missing observations of child outcomes and teacher-classroom variables, respectively. One child outcome, number of school absences, exceeded the cutoff of ± 2 skewness. This variable was transformed to 4 levels on each of the imputed datasets using intervals of 5-days absences for the first three levels (80% of the sample); 1 = 0-4 absences (29%); 2 = 5-9 absences (42%); 3 = 10-14 (10%); 4 = more than 15 absences (9%). Plots of the mean and variance of variable imputations against iteration number show a freely intermingled set of imputation streams, suggesting a healthy convergence as the variation between variable imputations is not high (Buuren & Groothuis-Oudshoorn, 2010; see Figure ).

*Figure 2A.*


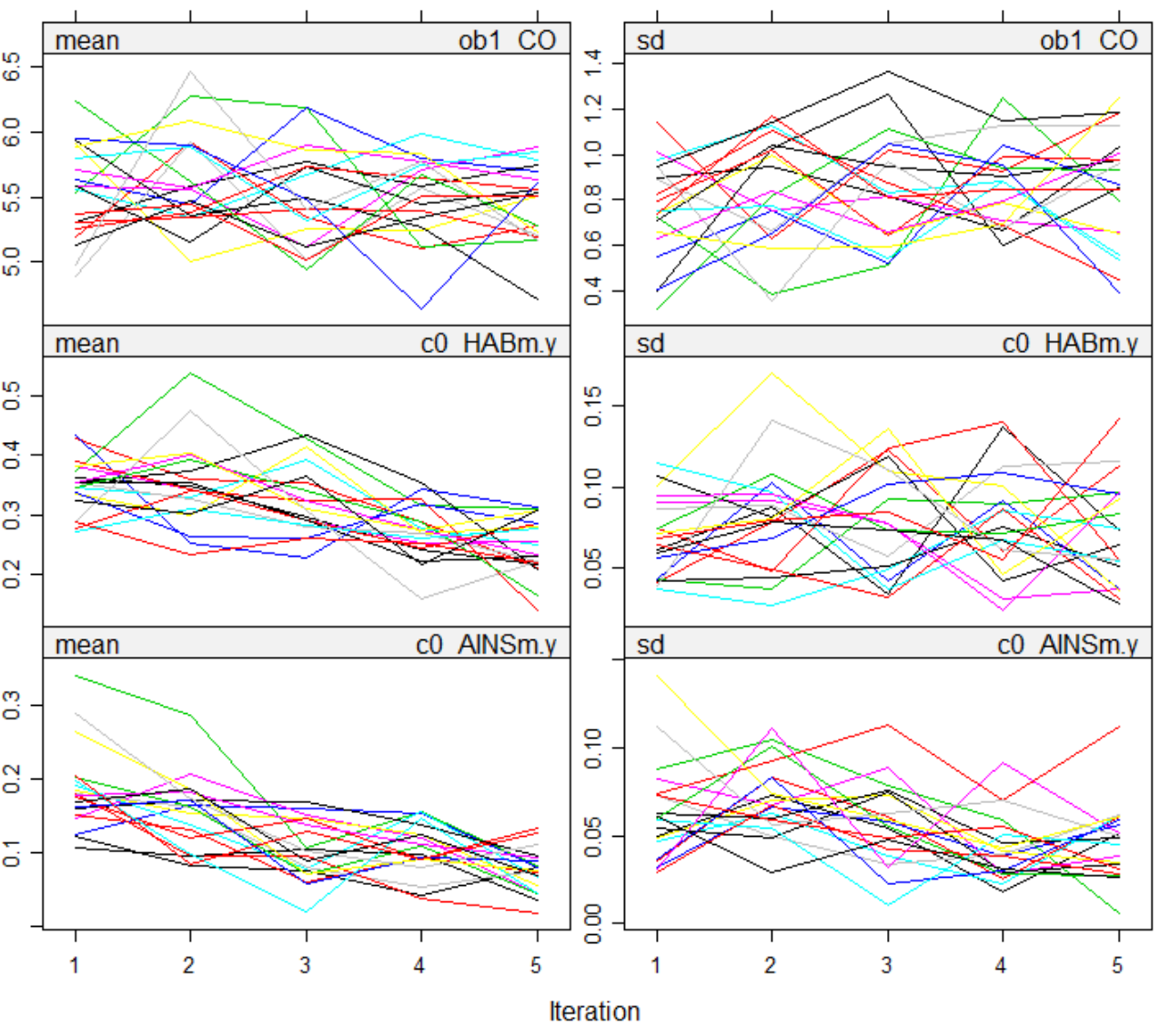

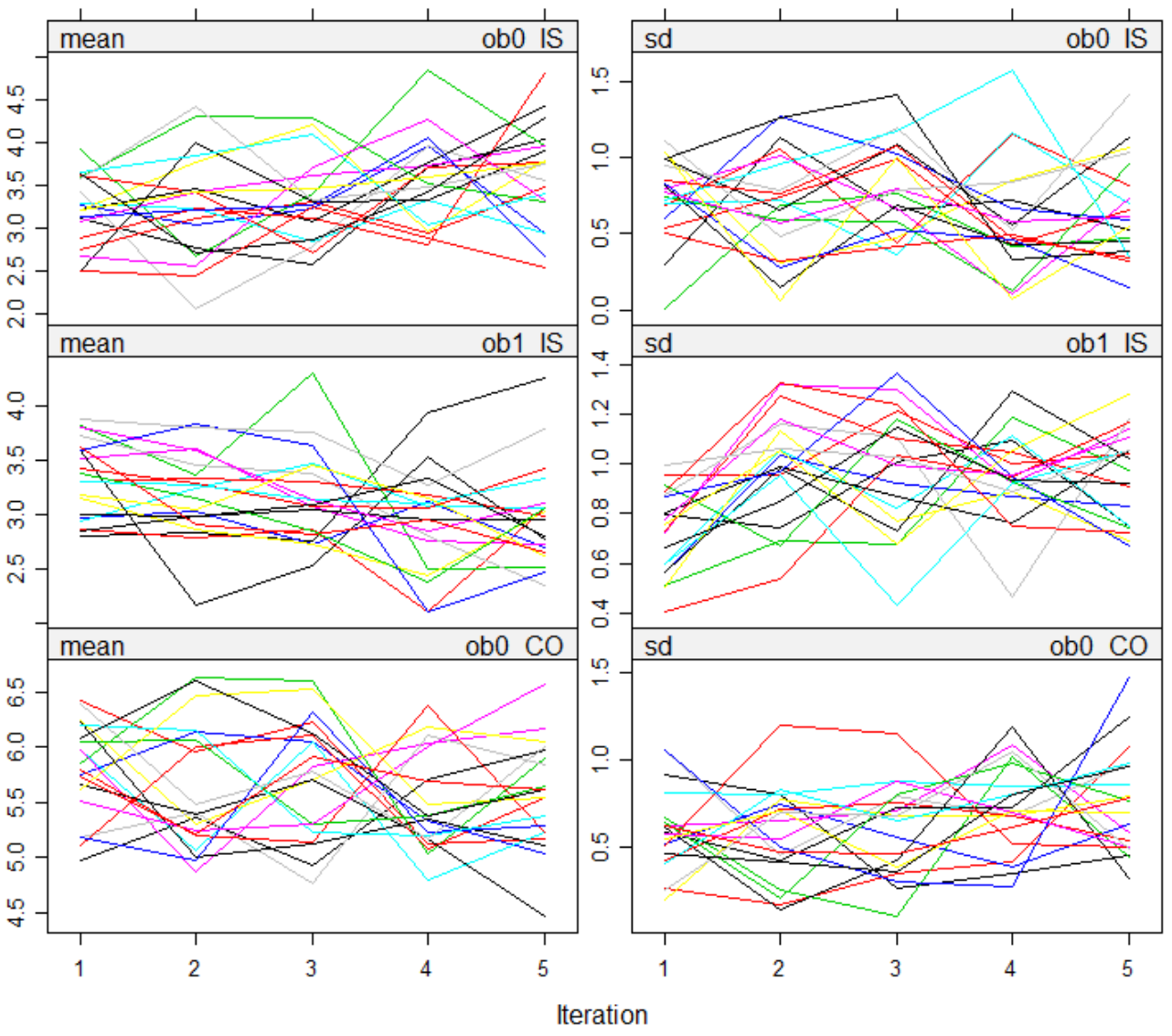

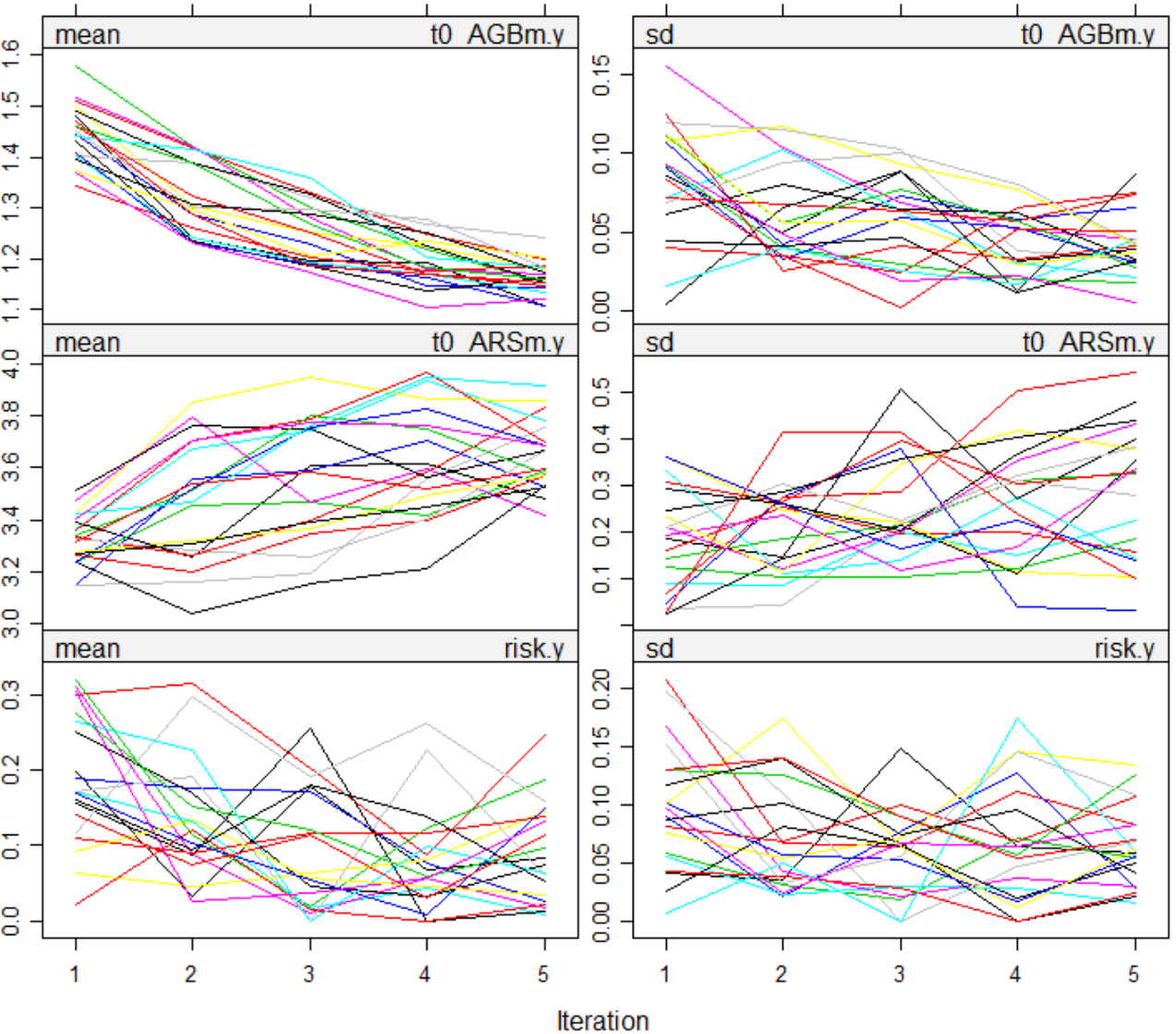

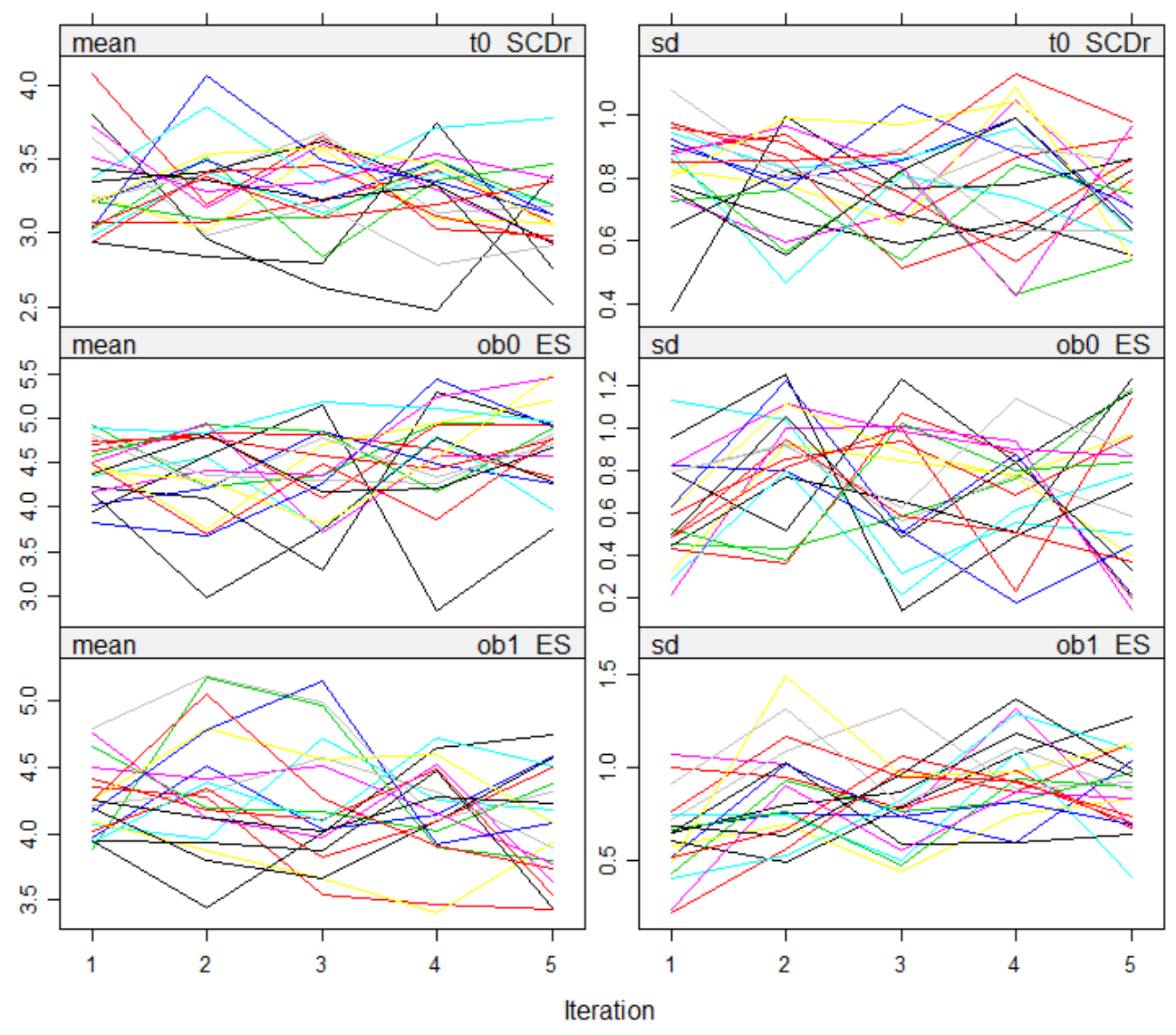

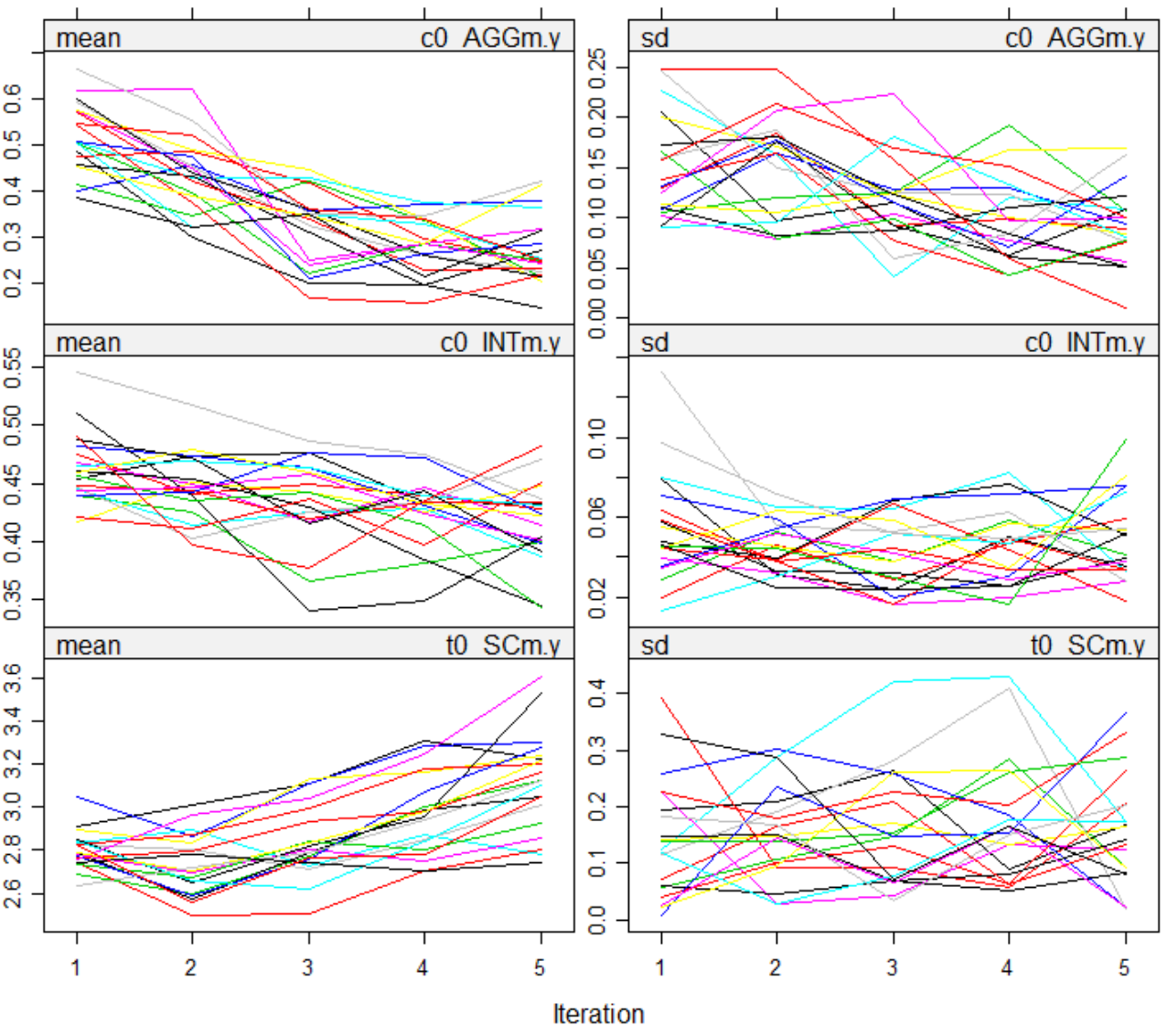

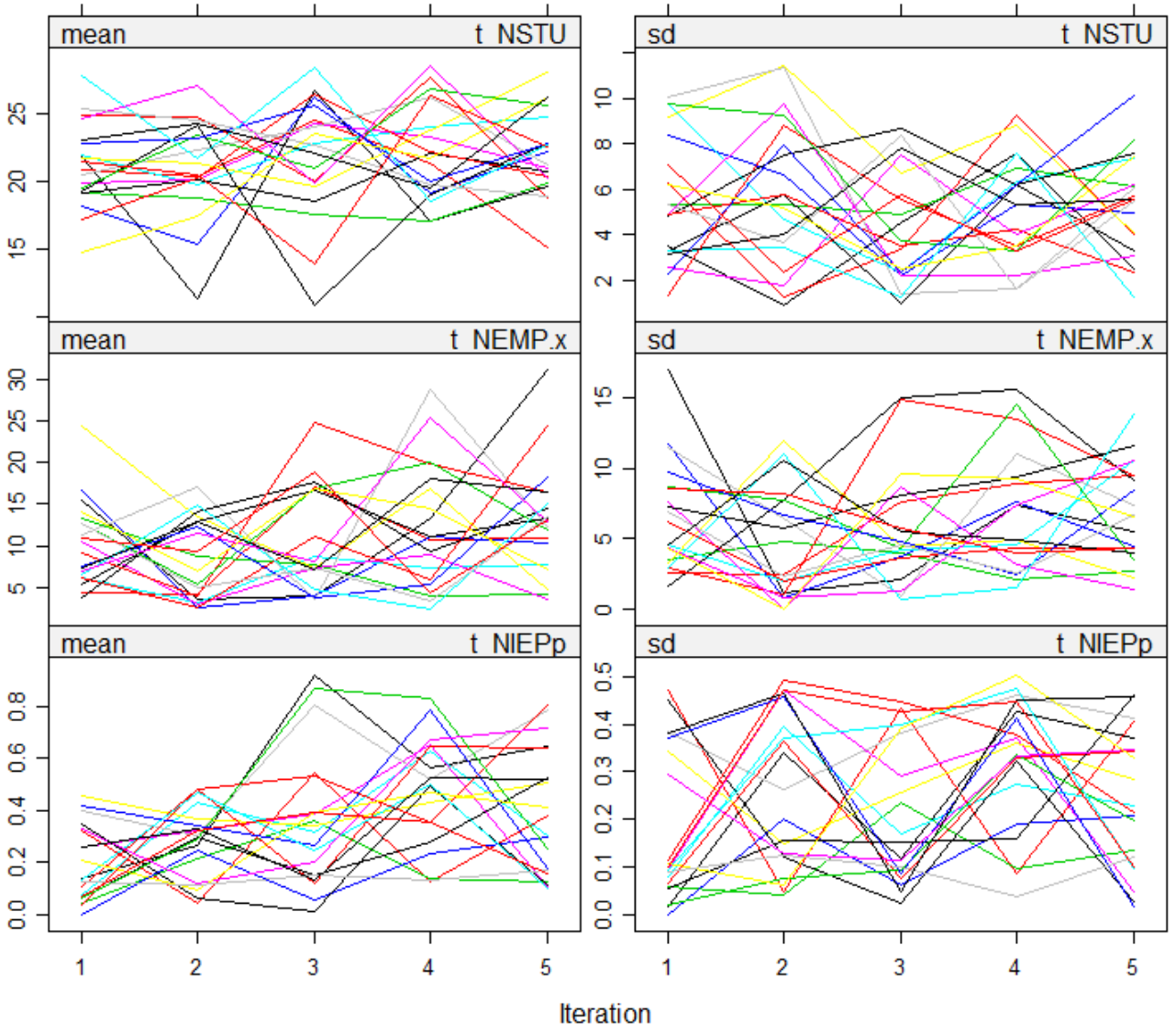


Plots of Means and Standard deviations against iteration number for each imputed variable

*Figure* *2A.*


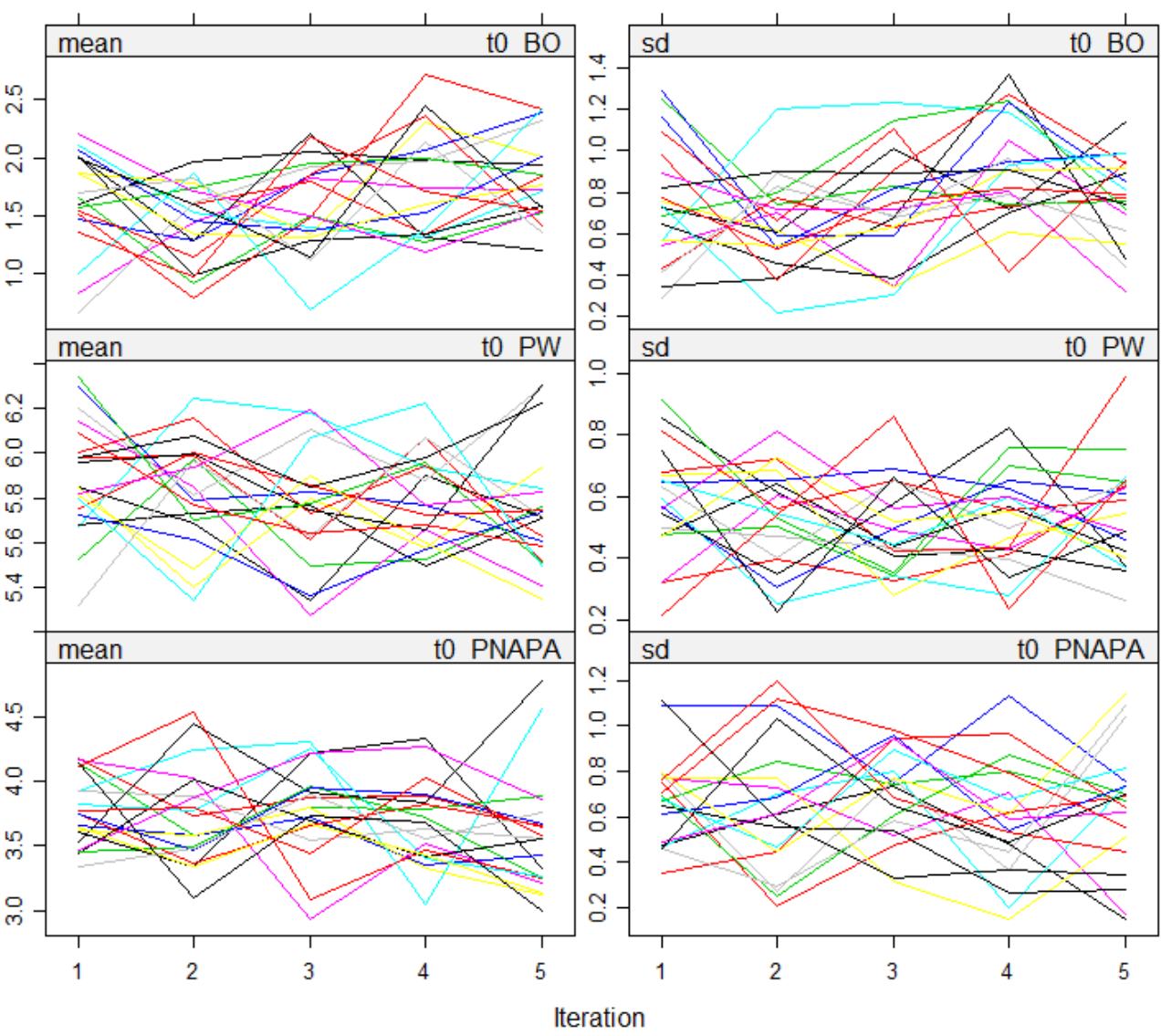

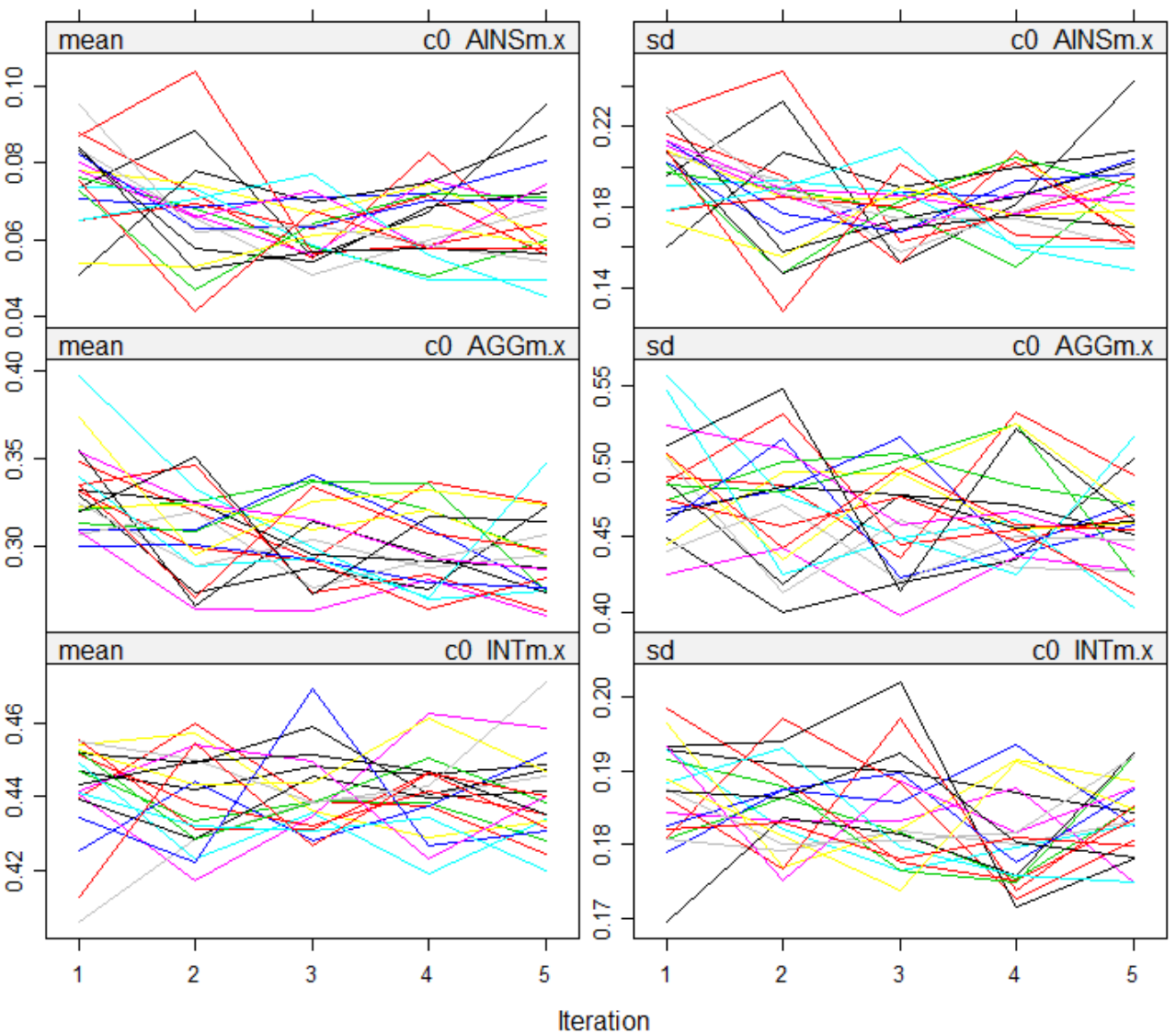

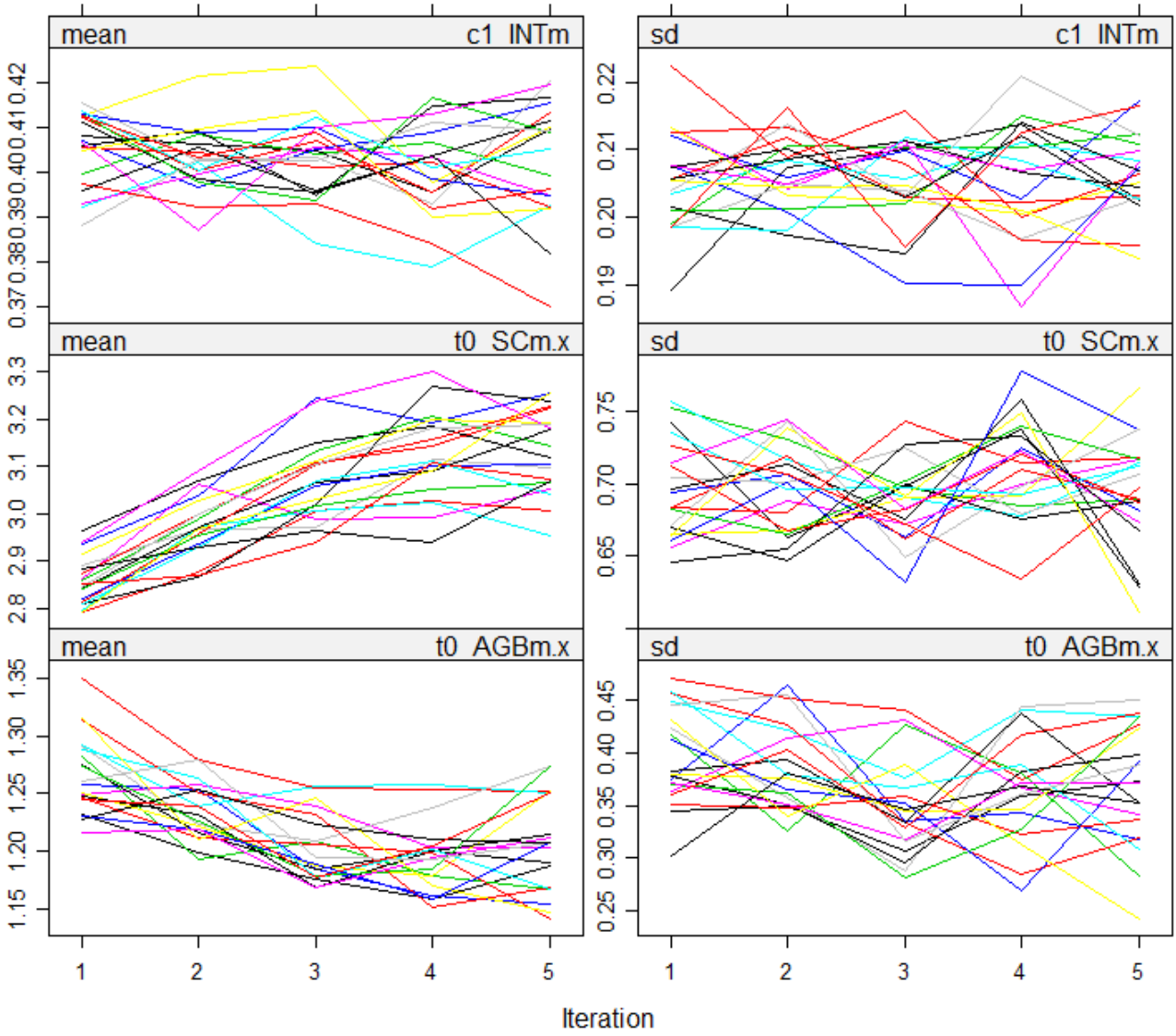

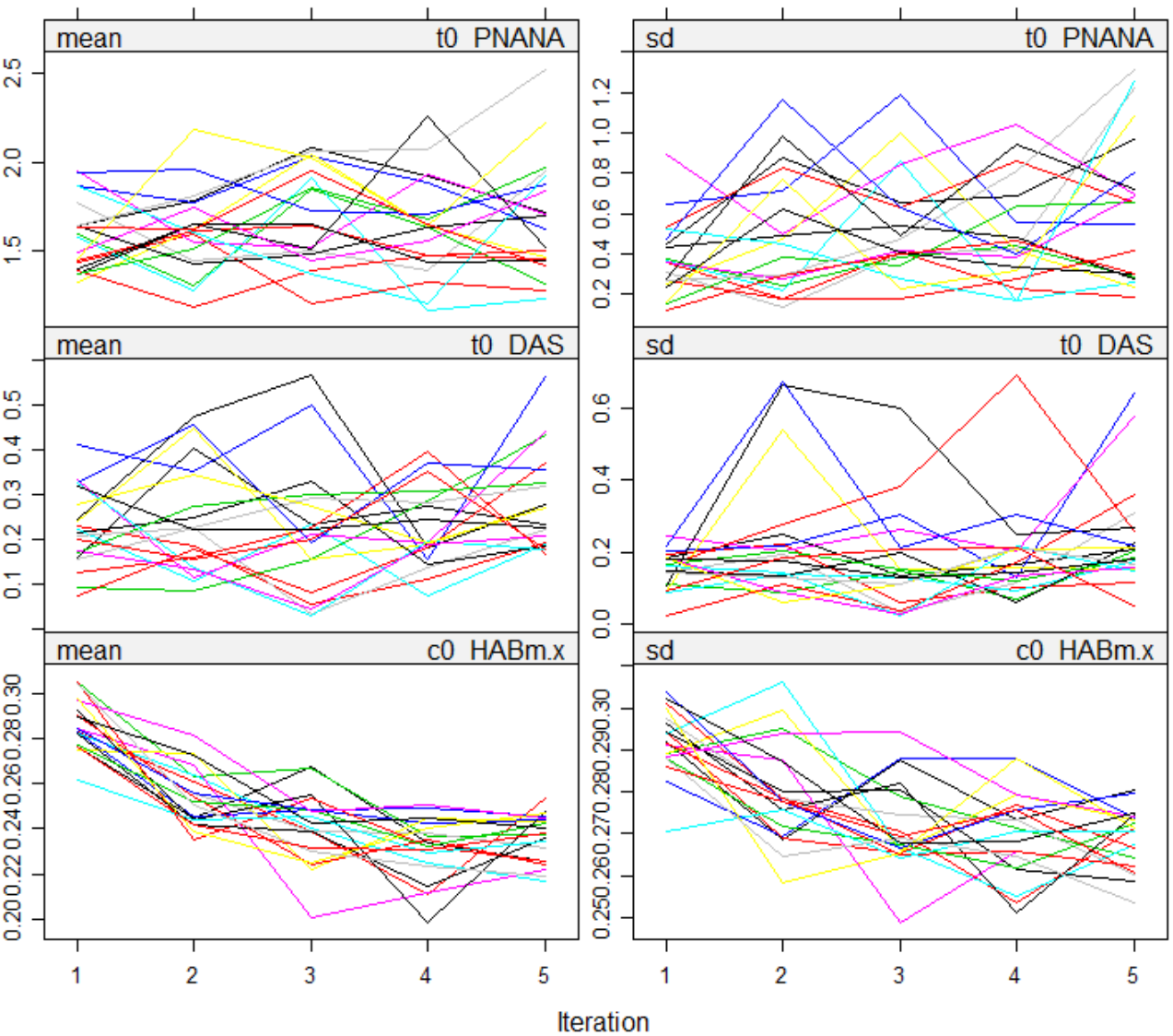

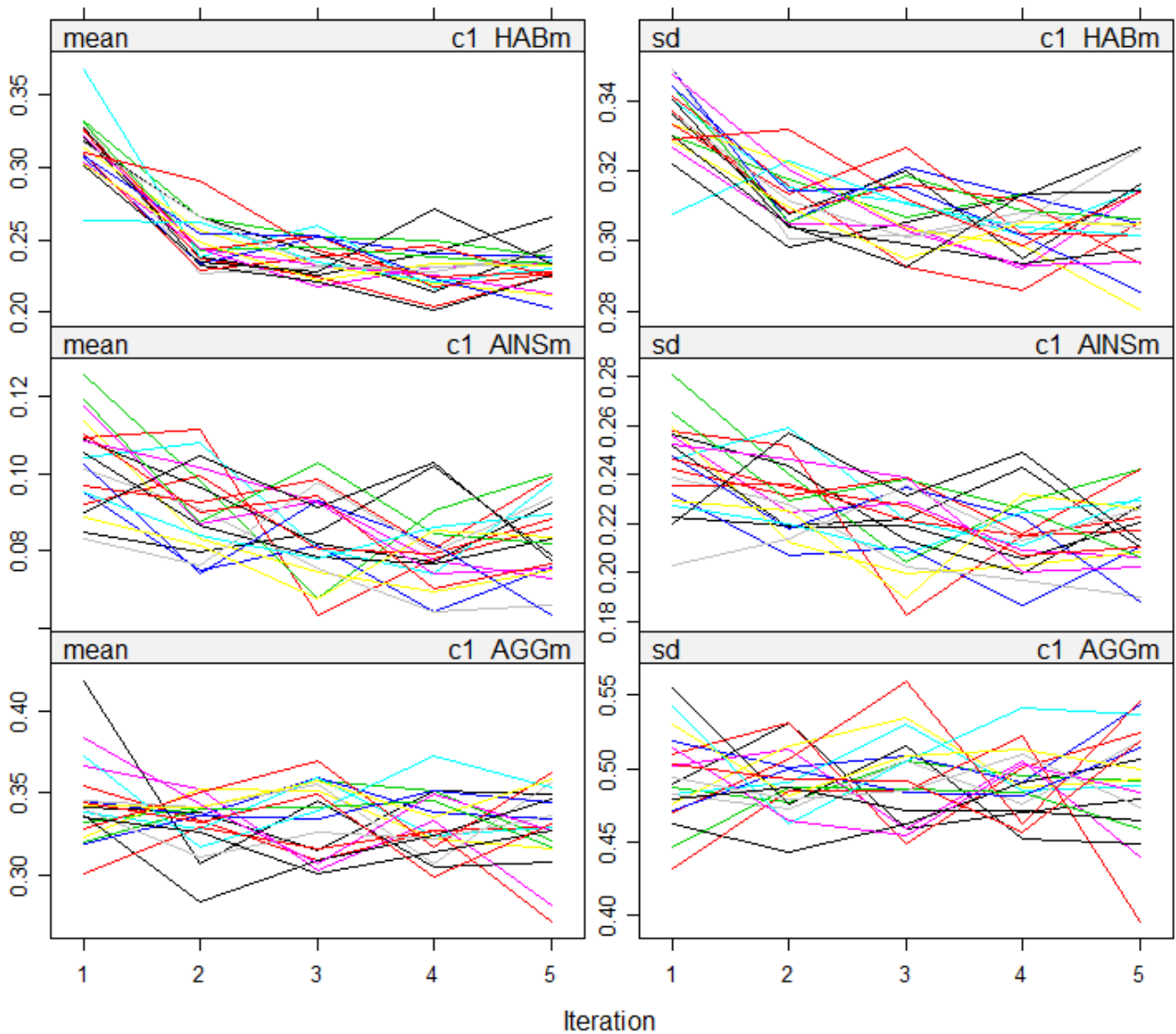

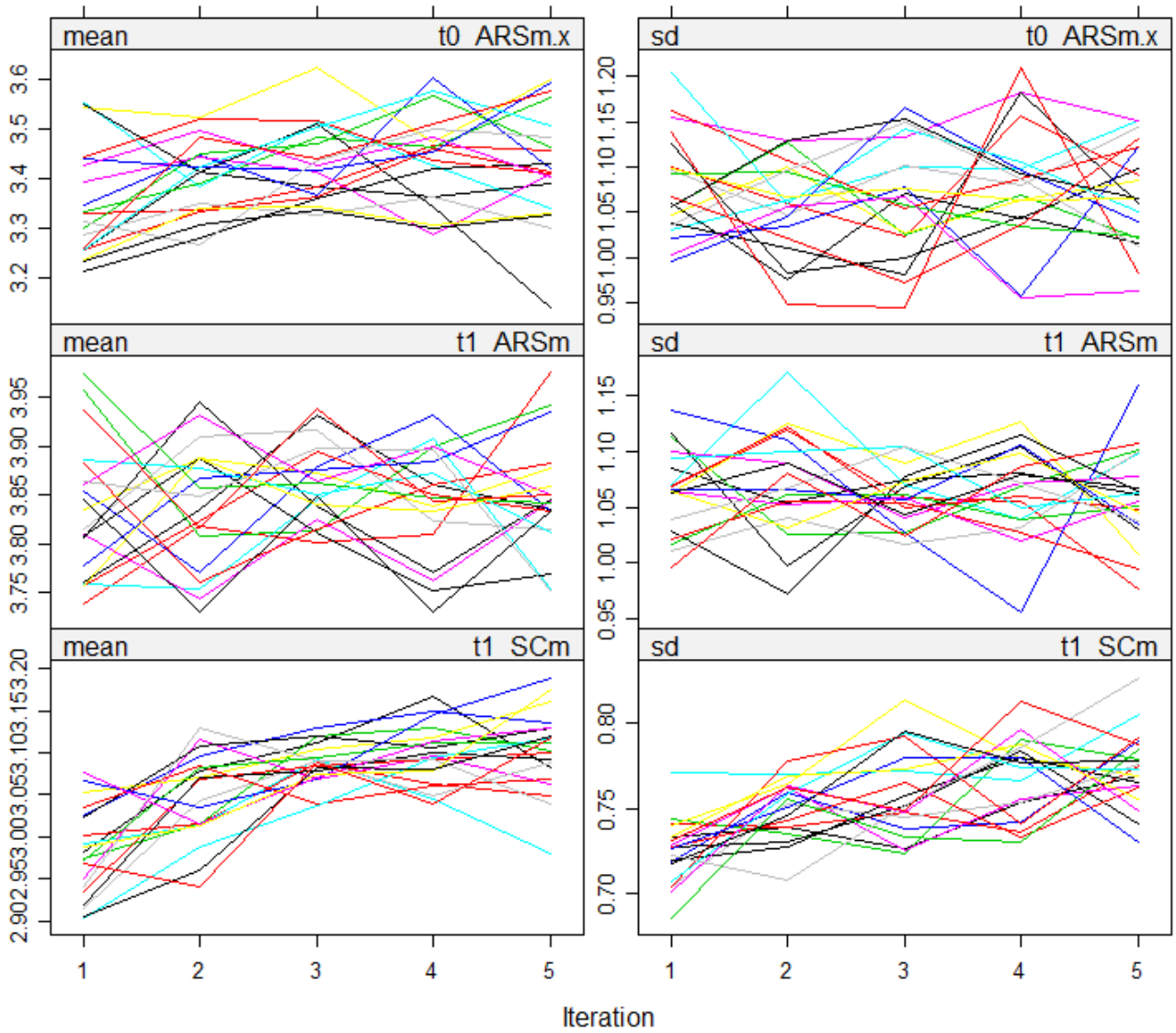


Plots of Means and Standard deviations against iteration number for each imputed variable

*Figure 2A.*


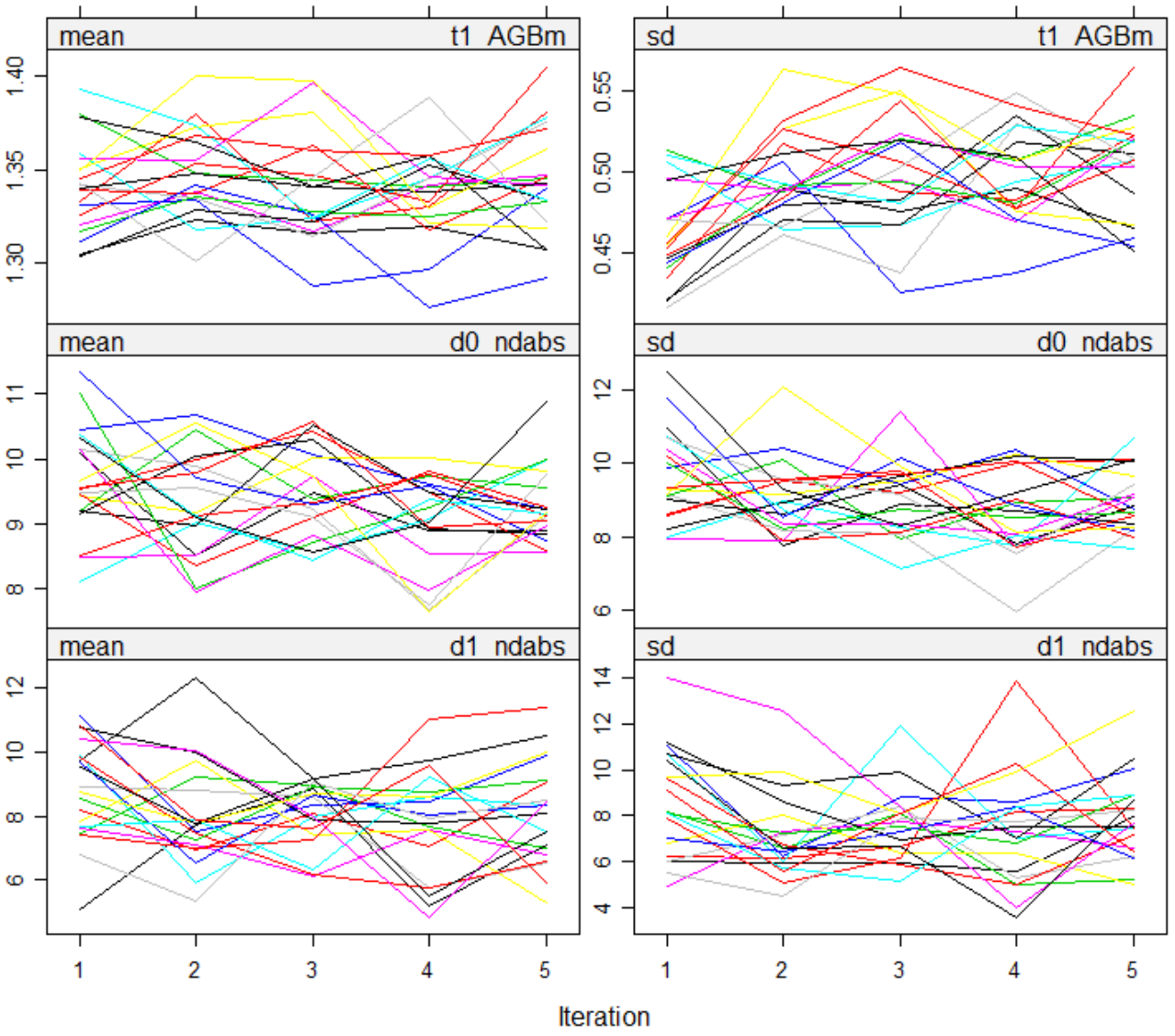

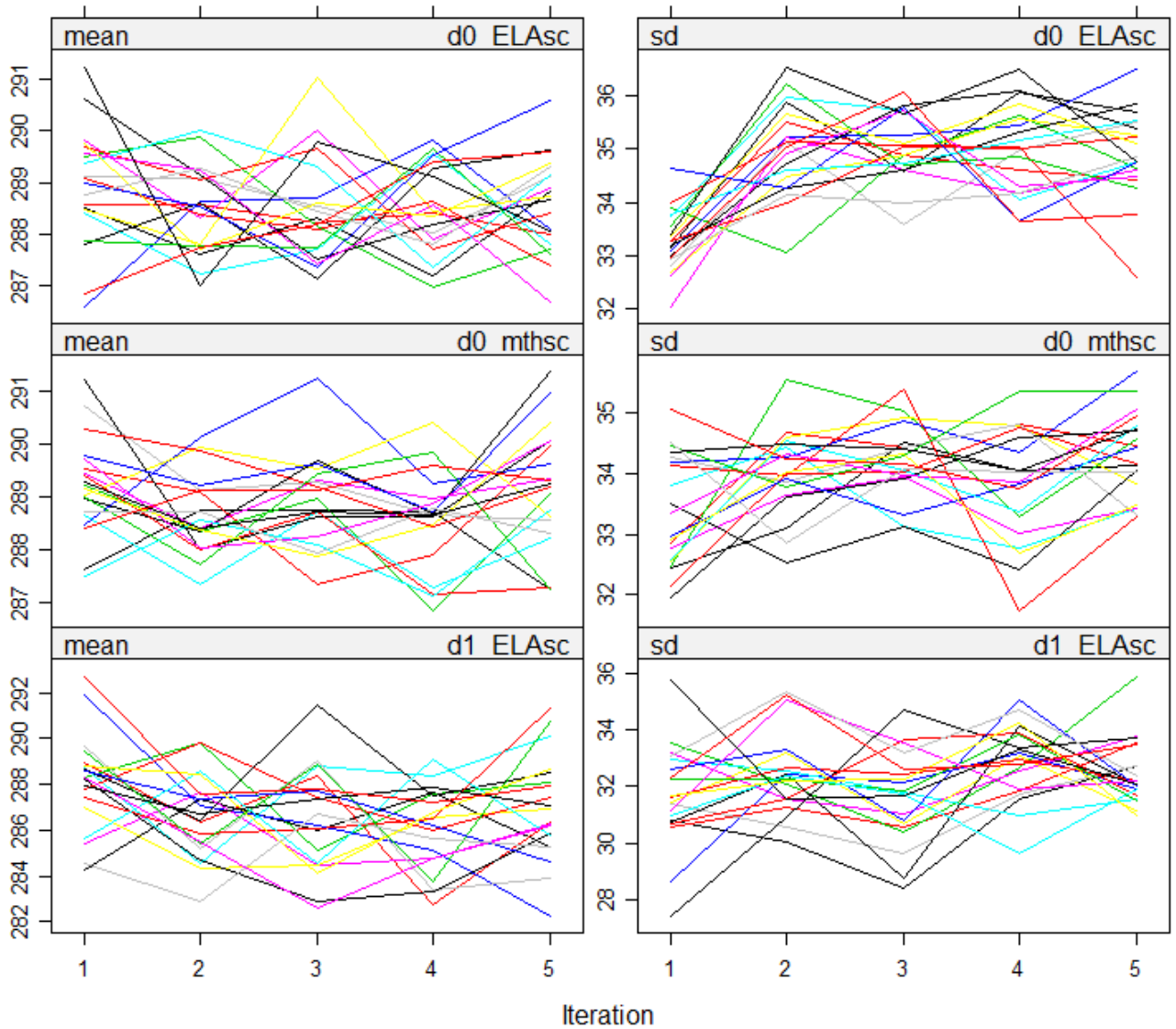

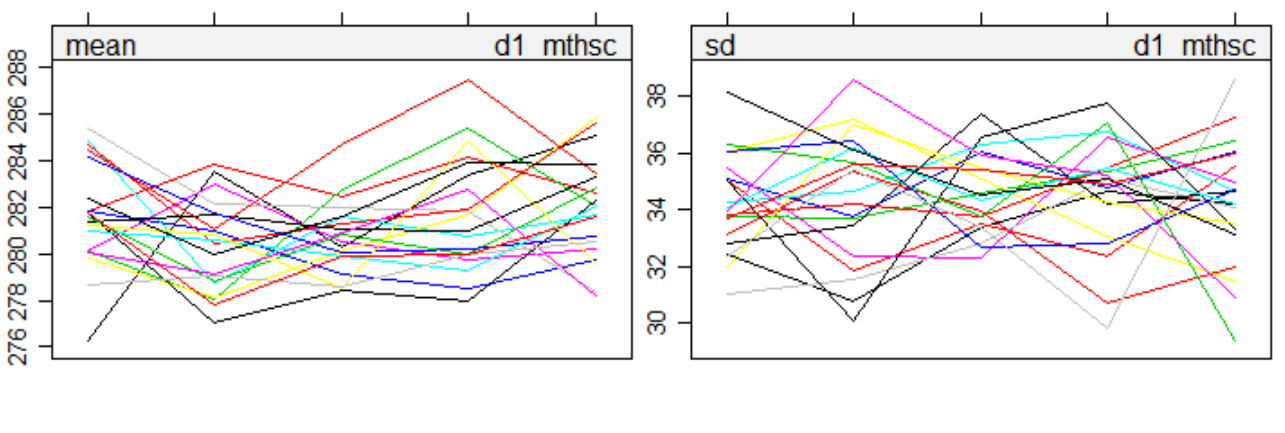


Plots of Means and Standard deviations against iteration number for each imputed variable
