# Supplementary material for: High quality implementation of 4Rs + MTP increases classroom emotional support and reduces absenteeism
Source: Front Psychol. 2023 Apr 27;14:1065749. doi: 10.3389/fpsyg.2023.1065749 (PMC10172679; doi:10.3389/fpsyg.2023.1065749)
Supplement: Supplementary file 4 [file Table_1.DOCX]

| Supplemental Table 1  *Correlation matrix of implementation variables* | | | | | | | | | | | | |
| --- | --- | --- | --- | --- | --- | --- | --- | --- | --- | --- | --- | --- |
|  | **1** | **2** | **3** | **4** | **5** | **6** | **7** | **8** | **9** | **10** | **11** | **12** |
| 1. Consultancy worth | 1.00 | .13 | .12 | -.19** | .24** | .05 | .05 | .22** | .07 | .07 | -.02 | .03 |
| 2. Teacher’s responsiveness to cycles |  | 1.00 | .40** | -.14 | .11 | .43** | .06 | .11 | .14 | .06 | .21** | -.11 |
| 3. Words in prompt responses |  |  | 1.00 | -.14 | -.03 | .19** | .04 | .09 | .32** | .23** | .21** | .17* |
| 4. Prompt’s access time elapsed |  |  |  | 1.00 | -.09 | -.12 | -.57** | -.28** | -.28** | -.41** | -.32** | -.09 |
| 5. Teacher’s responsiveness to training |  |  |  |  | 1.00 | .03 | .10 | .08 | -.02 | .01 | .07 | .03 |
| 6. Teacher-Coach alliance |  |  |  |  |  | 1.00 | .29** | -.29** | .19** | .12 | .30** | .27** |
| 7. Couching cycles completed |  |  |  |  |  |  | 1.00 | .24** | .26 | .30** | .30 | .06 |
| 8. Time in conferences |  |  |  |  |  |  |  | 1.00 | .15 | -.01 | .12 | -.27** |
| 9. Time spent visiting the website |  |  |  |  |  |  |  |  | 1.00 | .22** | .23** | .22** |
| 1. Attendance to training |  |  |  |  |  |  |  |  |  | 1.00 | .12 | .15* |
| 11. Adherence to program |  |  |  |  |  |  |  |  |  |  | 1.00 | .07 |
| 12. Exposure to program units |  |  |  |  |  |  |  |  |  |  |  | 1.00 |
| Signif. codes: ‘***’ 0.001 ‘**’ 0.01 ‘*’ 0.05 | | | | | | | | | | | | |
