# Supplementary material for: High quality implementation of 4Rs + MTP increases classroom emotional support and reduces absenteeism
Source: Front Psychol. 2023 Apr 27;14:1065749. doi: 10.3389/fpsyg.2023.1065749 (PMC10172679; doi:10.3389/fpsyg.2023.1065749)
Supplement: Supplementary file 5 [file Table_2.DOCX]

**Supplementary Table 2.** Frequency of Planned Program Activities Implemented by Teachers in Third Grade Classroom

| Grade 3 | Planned Classroom  Activities | Averaged amount of Classroom Activities Implemented | Teachers Implementing Classroom Activities | |
| --- | --- | --- | --- | --- |
|  | *N* | *M(Range)* | *N* | *%* |
| **Unit 1 Community** | **8** | **5.24 (1 -12)** | **75** | **90.4** |
| Book Talk | 2 | 2.00 | 71 | 85.5 |
| Lessons | 3 | 2.88 | 75 | 90.4 |
| Additional Activities | 3 | 1.32 | 25 | 30.1 |
| Your Own Activity | 0 | 1.00 | 2 | 2.4 |
| **Unit 2 Feelings** | **12** | **7.75 (2-16)** | **77** | **92.8** |
| Book Talk | 2 | 2.03 | 74 | 89.2 |
| Lessons | 5 | 4.47 | 77 | 92.8 |
| Additional Activities | 5 | 2.66 | 38 | 45.8 |
| Your Own Activity | 0 | 1.00 | 2 | 2.4 |
| **Unit 3 Listening** | **10** | **5.65 (1-11)** | **77** | **92.8** |
| Book Talk | 2 | 1.95 | 64 | 77.1 |
| Lessons | 4 | 3.56 | 68 | 81.9 |
| Additional Activities | 4 | 1.55 | 44 | 53.0 |
| Your Own Activity | 0 | 0.00 | 0 | 0.0 |
| **Unit 4 Assertiveness** | **10** | **5.7 (1-13)** | **63** | **76.0** |
| Book Talk | 2 | 1.96 | 57 | 68.7 |
| Lessons | 4 | 3.22 | 58 | 69.9 |
| Additional Activities | 4 | 1.67 | 36 | 43.4 |
| Your Own Activity | 0 | 0.00 | 0 | 0.0 |
| **Unit 5 Problem Solving** | **8** | **4.8 (1-11)** | **45** | **54.2** |
| Book Talk | 2 | 1.93 | 40 | 48.2 |
| Lessons | 3 | 2.63 | 40 | 48.2 |
| Additional Activities | 3 | 1.88 | 17 | 20.5 |
| Your Own Activity | 0 | 1.00 | 2 | 2.4 |
| **Unit 6 Diversity** | **10** | **6.24(1-45)** | **33** | **39.8** |
| Book Talk | 2 | 2.23 | 31 | 37.3 |
| Lessons | 5 | 4.03 | 29 | 34.9 |
| Additional Activities | 3 | 2.50 | 8 | 9.6 |
| Your Own Activity | 0 | 0.00 | 0 | 0.0 |
| **Unit 7 Making a Difference** | **8** | **4.58 (1-9)** | **19** | **22.9** |
| Book Talk | 2 | 1.81 | 16 | 19.3 |
| Lessons | 3 | 2.56 | 16 | 19.3 |
| Additional Activities | 3 | 1.70 | 10 | 12.0 |
| Your Own Activity | 0 | 0.00 | 0 | 0.0 |
| **Total** | **66** | **27.63 (1 -87)** | **83** | **100.0** |
