# Supplementary material for: High quality implementation of 4Rs + MTP increases classroom emotional support and reduces absenteeism
Source: Front Psychol. 2023 Apr 27;14:1065749. doi: 10.3389/fpsyg.2023.1065749 (PMC10172679; doi:10.3389/fpsyg.2023.1065749)
Supplement: Supplementary file 6 [file Table_3.DOCX]

**Supplementary Table 3.** Frequency of Planned Program Activities Implemented by Teachers in Fourth Grade Classroom

| Grade 4 | Planned Classroom  Activities | Averaged amount of Classroom Activities Implemented | Teachers Implementing Classroom Activities | |
| --- | --- | --- | --- | --- |
|  | *N* | *M* (Range) | *N* | *%* |
| **Unit 1 Community** | **10** | **5.23 (3-10)** | **60** | **95.2** |
| Book Talk | 3 | 1.97 | 58 | 92.1 |
| Lessons | 3 | 2.83 | 60 | 95.2 |
| Additional Activities | 4 | 1.86 | 14 | 22.2 |
| Your Own Activity | 0 | 1.00 | 4 | 6.3 |
| **Unit 2 Feelings** | **12** | **6.93(1-12)** | **61** | **96.8** |
| Book Talk | 2 | 1.87 | 55 | 87.3 |
| Lessons | 5 | 4.38 | 61 | 96.8 |
| Additional Activities | 5 | 1.56 | 27 | 42.9 |
| Your Own Activity | 0 | 1.00 | 11 | 17.5 |
| **Unit 3 Listening** | **10** | **5.95 (2-11)** | **56** | **88.9** |
| Book Talk | 2 | 1.90 | 50 | 79.4 |
| Lessons | 4 | 3.29 | 55 | 87.3 |
| Additional Activities | 4 | 1.69 | 29 | 46.0 |
| Your Own Activity | 0 | 1.00 | 8 | 12.7 |
| **Unit 4 Assertiveness** | **9** | **5.44 (1-12)** | **50** | **79.4** |
| Book Talk | 2 | 1.93 | 40 | 63.5 |
| Lessons | 4 | 3.08 | 50 | 79.4 |
| Additional Activities | 3 | 1.73 | 22 | 34.9 |
| Your Own Activity | 0 | 1.00 | 3 | 4.8 |
| **Unit 5 Problem Solving** | **10** | **5.97 (1-11)** | **39** | **61.9** |
| Book Talk | 2 | 1.97 | 30 | 47.6 |
| Lessons | 5 (+1 opt) | 4.05 | 37 | 58.7 |
| Additional Activities | 3 | 2.00 | 11 | 17.5 |
| Your Own Activity | 0 | 1.00 | 2 | 3.2 |
| **Unit 6 Diversity** | **11** | **5.63 (1-11)** | **30** | **47.6** |
| Book Talk | 2 | 1.86 | 22 | 34.9 |
| Lessons | 5 | 3.57 | 28 | 44.4 |
| Additional Activities | 4 | 2.17 | 12 | 19.0 |
| Your Own Activity | 0 | 1.00 | 2 | 3.2 |
| **Unit 7 Making a Difference** | **8** | **4.18 (1-8)** | **17** | **27.0** |
| Book Talk | 2 | 1.83 | 12 | 19.0 |
| Lessons | 3 | 2.19 | 16 | 25.4 |
| Additional Activities | 3 | 1.86 | 7 | 11.1 |
| Your Own Activity | 0 | 1.00 | 1 | 1.6 |
| **Total** | **70** | **28.81 (4-59)** | **63** | **100.0** |
