# Supplementary material for: High quality implementation of 4Rs + MTP increases classroom emotional support and reduces absenteeism
Source: Front Psychol. 2023 Apr 27;14:1065749. doi: 10.3389/fpsyg.2023.1065749 (PMC10172679; doi:10.3389/fpsyg.2023.1065749)
Supplement: Supplementary file 7 [file Table_4.docx]

Supplemental Table 4A.

*Main Predictors of Compliance by Random Forest Method*

|  | Random Forest |
| --- | --- |
| Predictors | Mean Decrease Difference (MDD) |
| Number of years employed as a teacher | .0151 |
| Professional Burnout | .0052 |
| Teacher negative affect | -.0003 |
| Teacher depression, anxiety and stress (aggregated) | -.0019 |
| Teacher positive affect | -.0022 |
| Psychological Well-Being | -.0006 |
| Number of students | -.0032 |
| Proportion of students with active IEP | -.0056 |
| Proportion of student considered at Risk | -.0073 |

Supplemental Table 4B.

*Regression coefficients and test statistics of Compliance predicting Quality of Implementation Variables*

|  | *b* | *SE* | $B$ | *t* | $R^{2}$ |
| --- | --- | --- | --- | --- | --- |
| **Teacher’s responsiveness to cycles** |  |  |  |  | .002 |
| Intercept | -.151 | .325 |  | -.466 |  |
| Compliance | .339 | .713 | .044 | .475 |  |
| **Consultancy worth** |  |  |  |  | .481 |
| Intercept | -2.582 | .234 |  | $-11.020***$ |  |
| Compliance | 5.861 | .515 | .689 | $11.390***$ |  |
| **Words in prompt responses** |  |  |  |  | .001 |
| Intercept | .139 | .326 |  | .426 |  |
| Compliance | -.278 | .716 | -.032 | -.382 |  |
| **Prompt access time elapsed** |  |  |  |  | .000 |
| Intercept | -.036 | .327 |  | -.110 |  |
| Compliance | .110 | .719 | .013 | .153 |  |
| **Teacher’s responsiveness to training** |  |  |  |  | .058 |
| Intercept | -.894 | .316 |  | $-2.826**$ |  |
| Compliance | 2.048 | .694 | .242 | $2.948**$ |  |
| **Teacher alliance** |  |  |  |  | .000 |
| Intercept | .026 | .295 |  | .089 |  |
| Compliance | .075 | .648 | .009 | .116 |  |
| **Couching cycles completed** |  |  |  |  | .000 |
| Intercept | .012 | .276 |  | .042 |  |
| Compliance | .119 | .607 | .014 | .197 |  |
| **Time in conferences** |  |  |  |  | .052 |
| Intercept | -.864 | .318 |  | $-2.718**$ |  |
| Compliance | 1.947 | .698 | .230 | $2.789**$ |  |
| **Attendance to training** |  |  |  |  | .001 |
| Intercept | .144 | .305 |  | .471 |  |
| Compliance | -.2156 | .670 | -.030 | -.322 |  |
| **Amount of program activities implemented in classroom** |  |  |  |  | .018 |
| Intercept | -.501 | .322 |  | -1.555 |  |
| Compliance | 1.140 | .708 | .144 | 1.610 |  |
| **Adherence to program activities** |  |  |  |  | .000 |
| Intercept | -.030 | .326 |  | -.093 |  |
| Compliance | .090 | .716 | .011 | .125 |  |

Signif. codes: 0 ‘***’ 0.001 ‘**’ 0.01

Degrees of Freedom=140
