# Supplementary material for: High quality implementation of 4Rs + MTP increases classroom emotional support and reduces absenteeism
Source: Front Psychol. 2023 Apr 27;14:1065749. doi: 10.3389/fpsyg.2023.1065749 (PMC10172679; doi:10.3389/fpsyg.2023.1065749)
Supplement: Supplementary file 8 [file Table_5.docx]

Supplementary Table 5.

*Covariate Balance*

| Number of students | | |  |  |
| --- | --- | --- | --- | --- |
|  | B | Std. Error | t | Sig. |
| Intercept | 17.74 | 2.05 | 8.64 | 0.00 |
| Above Average Compliance | -0.34 | 1.20 | -0.29 | 0.77 |
| Compliance Propensity | 13.48 | 5.67 | 2.38 | 0.02 |
| Random Assignment (Treatment) | -0.79 | 0.90 | -0.89 | 0.38 |
| Above Average X Random Assignment | -2.26 | 1.32 | -1.72 | 0.09 |
|  | | | | |
| Number of years employed as a teacher | | | | |
|  | B | Std. Error | t | Sig. |
| Intercept | -5.01 | 2.49 | -2.01 | 0.04 |
| Above Average Compliance | -3.47 | 1.45 | -2.39 | 0.02 |
| Compliance Propensity | 38.83 | 6.86 | 5.66 | 0.00 |
| Random Assignment (Treatment) | 1.22 | 1.09 | 1.12 | 0.26 |
| Above Average X Random Assignment | 1.89 | 1.60 | 1.18 | 0.24 |
|  |  |  |  |  |
| Proportion of students with active IEP | | | |  |
|  | B | Std. Error | t | Sig. |
| Intercept | 0.04 | 0.08 | 0.55 | 0.58 |
| Above Average Compliance | 0.01 | 0.05 | 0.30 | 0.76 |
| Compliance Propensity | 0.28 | 0.22 | 1.26 | 0.21 |
| Random Assignment (Treatment) | -0.01 | 0.04 | -0.29 | 0.77 |
| Above Average X Random Assignment | -0.05 | 0.05 | -0.90 | 0.37 |
|  |  |  |  |  |
| Professional Burnout | | |  |  |
|  | B | Std. Error | t | Sig. |
| Intercept | 2.88 | 0.28 | 10.29 | 0.00 |
| Above Average Compliance | -0.34 | 0.16 | -2.07 | 0.04 |
| Compliance Propensity | -2.87 | 0.77 | -3.71 | 0.00 |
| Random Assignment (Treatment) | 0.01 | 0.12 | 0.04 | 0.96 |
| Above Average X Random Assignment | 0.24 | 0.18 | 1.31 | 0.19 |
|  |  |  |  |  |
| Psychological Well-Being | | |  |  |
|  | B | Std. Error | t | Sig. |
| Intercept | 5.54 | 0.19 | 29.07 | 0.00 |
| Above Average Compliance | 0.28 | 0.11 | 2.49 | 0.01 |
| Compliance Propensity | 0.69 | 0.53 | 1.32 | 0.19 |
| Random Assignment (Treatment) | 0.11 | 0.08 | 1.31 | 0.19 |
| Above Average X Random Assignment | -0.19 | 0.12 | -1.59 | 0.11 |
|  |  |  |  |  |
| Teacher positive affect | | |  |  |
|  | B | Std. Error | t | Sig. |
| Intercept | 3.33 | 0.23 | 14.73 | 0.00 |
| Above Average Compliance | 0.45 | 0.13 | 3.37 | 0.00 |
| Compliance Propensity | 0.95 | 0.62 | 1.52 | 0.13 |
| Random Assignment (Treatment) | 0.07 | 0.10 | 0.74 | 0.46 |
| Above Average X Random Assignment | -0.14 | 0.15 | -0.99 | 0.32 |
|  |  |  |  |  |
| Teacher negative affect | | |  |  |
|  | B | Std. Error | t | Sig. |
| Intercept | 2.38 | 0.22 | 10.75 | 0.00 |
| Above Average Compliance | -0.18 | 0.13 | -1.37 | 0.17 |
| Compliance Propensity | -1.33 | 0.61 | -2.19 | 0.03 |
| Random Assignment (Treatment) | -0.09 | 0.10 | -0.98 | 0.33 |
| Above Average X Random Assignment | 0.14 | 0.14 | 0.97 | 0.33 |
|  |  |  |  |  |
| Teacher depression, anxiety and stress | | | |  |
|  | B | Std. Error | t | Sig. |
| Intercept | 0.52 | 0.11 | 4.72 | 0.00 |
| Above Average Compliance | -0.06 | 0.06 | -0.95 | 0.34 |
| Compliance Propensity | -0.47 | 0.31 | -1.54 | 0.13 |
| Random Assignment (Treatment) | -0.09 | 0.05 | -1.94 | 0.05 |
| Above Average X Random Assignment | 0.06 | 0.07 | 0.89 | 0.38 |
|  |  |  |  |  |
| Proportion of student considered at Risk | | | | |
|  | B | Std. Error | t | Sig. |
| Intercept | 0.12 | 0.07 | 1.80 | 0.07 |
| Above Average Compliance | 0.01 | 0.04 | 0.30 | 0.77 |
| Compliance Propensity | 0.12 | 0.19 | 0.63 | 0.53 |
| Random Assignment (Treatment) | 0.05 | 0.03 | 1.61 | 0.11 |
| Above Average X Random Assignment | 0.00 | 0.04 | -0.10 | 0.92 |
