# Supplementary material for: High quality implementation of 4Rs + MTP increases classroom emotional support and reduces absenteeism
Source: Front Psychol. 2023 Apr 27;14:1065749. doi: 10.3389/fpsyg.2023.1065749 (PMC10172679; doi:10.3389/fpsyg.2023.1065749)
Supplement: Supplementary file 9 [file Table_6.docx]

Supplementary Table 6

*Regression coefficients and test statistics for Quality of Classroom Interactions*

|  | $b$ | $SE$ | $B$ | $T$ | $ⅆf$ | $R^{2}$ |
| --- | --- | --- | --- | --- | --- | --- |
| **Emotional Support** | | |  |  |  | .23 |
| Intercept | 4.48 | .08 | 5.01 | ${53.73}^{***}$ | 4409.95 |  |
| TX | .15 | .09 | .08 | 1.56 | 1611.68 |  |
| Compliance | -1.10 | .72 | -.13 | -1.54 | 685.89 |  |
| TX*Compliance | .99 | .90 | .09 | 1.10 | 1415.79 |  |
| Cohort | -.45 | .10 | -.25 | ${-4.74}^{***}$ | 3305.51 |  |
| Time 1 Emotional Support | .33 | .05 | .31 | ${7.13}^{***}$ | 603.26 |  |
| **Instructional Support** | | |  |  |  | .16 |
| Intercept | 3.53 | .09 | 4.06 | ${40.98}^{***}$ | 4441.80 |  |
| TX | -.05 | .10 | -.03 | -.49 | 2438.37 |  |
| Compliance | .01 | .70 | .00 | .01 | 1512.70 |  |
| TX*Compliance | .48 | .88 | .04 | .54 | 4385.81 |  |
| Cohort | -.36 | .10 | -.20 | ${-3.66}^{***}$ | 3756.20 |  |
| Time 1 Instructional Support | .26 | .05 | .24 | ${4.92}^{***}$ | 940.18 |  |
| **Classroom Organization** | | |  |  |  | .27 |
| Intercept | 6.02 | .07 | 8.23 | ${90.95}^{***}$ | 2720.23 |  |
| TX | -.10 | .08 | -.07 | -1.26 | 5146.18 |  |
| Compliance | -.85 | .55 | -.12 | -1.55 | 3598.66 |  |
| TX*Compliance | .38 | .77 | .04 | .49 | 3641.16 |  |
| Cohort | -.07 | .08 | -.05 | -.87 | 6131.88 |  |
| Time 1 Classroom Organization | .33 | .06 | .32 | ${5.76}^{***}$ | 1148.63 |  |

Note: TX: Random assignment to 4Rs+MTP (1) versus Control (0).
