# Supplementary material for: High quality implementation of 4Rs + MTP increases classroom emotional support and reduces absenteeism
Source: Front Psychol. 2023 Apr 27;14:1065749. doi: 10.3389/fpsyg.2023.1065749 (PMC10172679; doi:10.3389/fpsyg.2023.1065749)
Supplement: Supplementary file 10 [file Table_7.docx]

Supplementary Table 7

Regression Coefficients and Test Statistics for Children’s Academic and SEL Outcomes.

|  | $b$ | | $SE$ | $B$ | $Z$ | $R^{2}$ |
| --- | --- | --- | --- | --- | --- | --- |
| **Hostile Attribution Bias** | | | |  |  | .84 |
| Intercept | | .40 | .04 | 5.44 | ${9.34}^{***}$ |  |
| TX | | .00 | .01 | .00 | -.04 |  |
| Compliance | | -.05 | .07 | -.07 | -.75 |  |
| TX*Compliance | | .01 | .10 | .01 | .08 |  |
| Emotional Support | | .00 | .01 | -.02 | -.18 |  |
| Instructional Support | | .00 | .01 | -.02 | -.15 |  |
| Classroom Organization | | -.01 | .01 | -.07 | -.84 |  |
| Cohort | | .00 | .01 | .02 | .33 |  |
| T 1 Hostile Attribution Bias | | .55 | .04 | .90 | ${13.19}^{***}$ |  |
| **Aggressive Interpersonal Strategies** | | | | |  | .63 |
| Intercept | .28 | | .04 | 3.48 | ${6.64}^{***}$ |  |
| TX | .01 | | .01 | .05 | .75 |  |
| Compliance | .00 | | .08 | .00 | -.03 |  |
| TX*Compliance | -.03 | | .10 | -.03 | -.29 |  |
| Emotional Support | -.01 | | .01 | -.08 | -.78 |  |
| Instructional Support | .01 | | .01 | .06 | .71 |  |
| Classroom Organization | -.02 | | .01 | -.14 | ${-1.77}^{t}$ |  |
| Cohort | .01 | | .01 | .08 | 1.22 |  |
| T1 Aggressive Interpersonal Strategies | .50 | | .04 | .73 | ${12.31}^{***}$ |  |
| **Internalizing Symptoms** | | | |  |  | .88 |
| Intercept | .46 | | .03 | 9.19 | ${15.68}^{***}$ |  |
| TX | .00 | | .01 | -.02 | -.32 |  |
| Compliance | -.04 | | .05 | -.08 | -.79 |  |
| TX*Compliance | .05 | | .06 | .08 | .84 |  |
| Emotional Support | .00 | | .01 | -.04 | -.33 |  |
| Instructional Support | .01 | | .01 | .10 | 1.02 |  |
| Classroom Organization | -.01 | | .01 | -.15 | ${-1.65}^{t}$ |  |
| Cohort | .01 | | .01 | .09 | 1.21 |  |
| Time 1 Internalizing | .60 | | .05 | .92 | ${12.09}^{***}$ |  |

Note: TX= Random assignment to 4Rs+MTP (1) versus Control (0); T1=Time 1
