# Supplementary material for: High quality implementation of 4Rs + MTP increases classroom emotional support and reduces absenteeism
Source: Front Psychol. 2023 Apr 27;14:1065749. doi: 10.3389/fpsyg.2023.1065749 (PMC10172679; doi:10.3389/fpsyg.2023.1065749)
Supplement: Supplementary file 12 [file Table_9.DOCX]

Supplementary Table 8

Conditional Direct (Path “c”), Mediated (Path “a” X “b”) and Total Effects

| **Paths** | **Compliance Propensity** | | | | | | | | | | | |
| --- | --- | --- | --- | --- | --- | --- | --- | --- | --- | --- | --- | --- |
|  | **Below Average** | | | | | | **Above Average** | | | | | |
|  | $b$ | $SE$ | $B$ | $Z$ | 95% CI | | $b$ | $SE$ | $B$ | $Z$ | 95% CI | |
|  |  |  |  |  | Lower | Upper |  |  |  |  | Lower | Upper |
| **HAB** |  |  |  |  |  |  |  |  |  |  |  |  |
| Direct effect | .00 | .016 | -.01 | -.07 | -.032 | .030 | .00 | .014 | .01 | .03 | -.027 | .028 |
| Indirect ES | .00 | .001 | .00 | -.19 | -.001 | .001 | .00 | .003 | .00 | -.14 | -.006 | .005 |
| Indirect IS | .00 | .000 | .00 | .47 | .000 | .001 | .00 | .001 | .00 | -.08 | -.001 | .001 |
| Indirect CO | .00 | .001 | .01 | .88 | -.001 | .003 | .00 | .001 | .00 | .36 | -.002 | .003 |
| Total effect | .00 | .016 | .00 | -.01 | -.031 | .031 | .00 | .014 | .00 | .03 | -.028 | .028 |
| **AINS** |  |  |  |  |  |  |  |  |  |  |  |  |
| Direct Effect | .01 | .015 | 0.08 | .77 | -.017 | .000 | .00 | .015 | .00 | .40 | -.024 | .035 |
| Indirect ES | .00 | .001 | 0.00 | -.30 | -.002 | .000 | .00 | .003 | .00 | -.60 | -.008 | .004 |
| Indirect IS | .00 | .000 | 0.00 | -1.44 | -.001 | .000 | .00 | .001 | .00 | .00 | -.001 | .001 |
| Indirect CO | .00 | .001 | 0.02 | 1.53 | -.001 | .000 | .00 | .001 | .00 | .60 | -.002 | .004 |
| Total effect | .01 | .015 | 0.09 | .85 | -.016 | .000 | .00 | .015 | .00 | .30 | -.025 | .034 |
| **INT** |  |  |  |  |  |  |  |  |  |  |  |  |
| Direct Effect | -.01 | .009 | -.11 | -.85 | -.026 | .010 | .00 | .011 | .06 | .29 | -.018 | .024 |
| Indirect ES | .00 | .000 | .00 | -.26 | -.001 | .000 | .00 | .002 | -.01 | -.25 | -.004 | .003 |
| Indirect IS | .00 | .001 | -.01 | -.98 | -.002 | .001 | .00 | .001 | .00 | .05 | -.001 | .001 |
| Indirect CO | .00 | .002 | .02 | .90 | -.002 | .004 | .00 | .002 | .00 | .33 | -.003 | .004 |
| Total effect | -.01 | .009 | -.10 | -.76 | -.026 | .011 | .00 | .011 | .06 | .30 | -.018 | .024 |
| **ABC** |  |  |  |  |  |  |  |  |  |  |  |  |
| Direct Effect | .03 | .031 | .11 | 1.00 | -.030 | .092 | -.01 | .029 | -.04 | -.18 | -.062 | .051 |
| Indirect ES | .00 | .003 | .00 | -.50 | -.007 | .004 | -.01 | .006 | -.02 | -1.12 | -.020 | .005 |
| Indirect IS | .00 | .003 | .00 | .43 | -.004 | .007 | .00 | .003 | .00 | .00 | -.006 | .006 |
| Indirect CO | .00 | .004 | .01 | 1.18 | -.003 | .011 | .00 | .003 | .00 | .48 | -.005 | .009 |
| Total effect | .03 | .031 | .11 | 1.04 | -.029 | .094 | -.01 | .029 | -.06 | -.36 | -.068 | .047 |
| **ABT** |  |  |  |  |  |  |  |  |  |  |  |  |
| Direct Effect | -.02 | .021 | -.07 | -1.10 | -.065 | .018 | .01 | .022 | .04 | .33 | -.036 | .051 |
| Indirect ES | .00 | .002 | .00 | -.48 | -.004 | .002 | .00 | .004 | -.01 | -.88 | -.011 | .004 |
| Indirect IS | .00 | .001 | .00 | -1.36 | -.002 | .000 | .00 | .001 | .00 | .04 | -.002 | .002 |
| Indirect CO | .00 | .002 | .01 | 1.04 | -.002 | .007 | .00 | .003 | .00 | .40 | -.004 | .006 |
| Total effect | -.02 | .021 | -.07 | -1.04 | -.064 | .020 | .01 | .023 | .03 | .21 | -.040 | .050 |

Note: **Direct Effect**: effect of treatment on child outcome, **Mediation ES**: Effect of treatment on child outcome as mediated by emotional support, **Mediation IS**: Effect of treatment on child outcome as mediated by instructional support, **Mediation CO**: Effect of treatment on child outcome as mediated by classroom organization.

Supplementary Table 7

Conditional Direct (Path “c”), Mediated (Path “a” X “b”) and Total Effects

|  | **Compliance Propensity** | | | | | | | | | | | | |
| --- | --- | --- | --- | --- | --- | --- | --- | --- | --- | --- | --- | --- | --- |
|  | **Below Average** | | | | | | **Below Average** | | | | | | |
| **Paths** | $b$ | $SE$ | $B$ | $Z$ | 95% CI | | $b$ | $SE$ | $B$ | $Z$ | 95% CI | | |
|  |  |  |  |  | Lower | Upper |  |  |  |  | Lower | | Upper |
| **SC** |  |  |  |  |  |  |  |  |  |  |  |  | |
| Direct Effect | .06 | .041 | .09 | 1.37 | -.024 | .137 | .02 | .043 | .01 | .42 | -.066 | .101 | |
| Indirect ES | .00 | .002 | .00 | .55 | -.003 | .006 | .01 | .007 | .01 | .93 | -.007 | .021 | |
| Indirect IS | .00 | .002 | .00 | -.35 | -.005 | .004 | .00 | .003 | .00 | -.01 | -.006 | .006 | |
| Indirect CO | .00 | .002 | .00 | -.05 | -.004 | .004 | .00 | .002 | .00 | .01 | -.004 | .004 | |
| Total effect | .06 | .041 | .09 | 1.39 | -.023 | .137 | .03 | .043 | .02 | .58 | -.059 | .108 | |
| **ACAD** |  |  |  |  |  |  |  |  |  |  |  |  | |
| Direct Effect | -.20 | 3.624 | .00 | -.05 | -7.299 | 6.908 | -.31 | 3.298 | -.01 | -.09 | -6.772 | 6.157 | |
| Indirect ES | -.11 | .522 | .00 | -.21 | -1.133 | .914 | -.78 | .936 | -.02 | -.84 | -2.621 | 1.055 | |
| Indirect IS | -.28 | .383 | -.01 | -.74 | -1.033 | .469 | .01 | .444 | .00 | .02 | -.864 | .878 | |
| Indirect CO | -.64 | .649 | -.02 | -.98 | -1.906 | .637 | -.27 | .706 | .00 | -.38 | -1.652 | 1.118 | |
| Total effect | -1.22 | 3.598 | -.03 | -.34 | -8.274 | 5.83 | -1.35 | 3.307 | -.03 | -.41 | -7.832 | 5.131 | |
| **ABS** |  |  |  |  |  |  |  |  |  |  |  |  | |
| Direct Effect | .03 | .039 | .11 | .80 | -.045 | .107 | -.08 | .038 | -.20 | ${-2.05}^{*}$ | -.154 | -.003 | |
| Indirect ES | .00 | .001 | .00 | .33 | -.002 | .003 | .00 | .007 | .01 | .46 | -.010 | .016 | |
| Indirect IS | .00 | .002 | .00 | 1.03 | -.002 | .005 | .00 | .002 | .00 | -.03 | -.005 | .005 | |
| Indirect CO | .00 | .004 | .01 | .93 | -.004 | .012 | .00 | .004 | .00 | .39 | -.007 | .010 | |
| Total effect | .04 | .039 | .12 | .96 | -.039 | .113 | -.07 | .036 | -.19 | -${2.05}^{*}$ | -.144 | -.003 | |

Note: **Direct Effect**: effect of treatment on child outcome, **Mediation ES**: Effect of treatment on child outcome as mediated by emotional support, **Mediation IS**: Effect of treatment on child outcome as mediated by instructional support, **Mediation CO**: Effect of treatment on child outcome as mediated by classroom organization.
